# Development of G Protein-Coupled Receptor 183 Benzimidazole Antagonists Utilizing a Core-Hopping Approach

**DOI:** 10.3390/molecules31193469

**Published:** 2026-09-29

**Authors:** Angelina Ellis, Israel Olayide, Vanja Kalajdzic, Daniela Salvemini, Christopher Arnatt

**Affiliations:** 1 Department of Chemistry, Saint Louis University, St. Louis, MO 63103, USA; 2 Department of Pharmacology and Physiology, Saint Louis University, St. Louis, MO 63103, USA; 3 Institute for Drug and Biotherapeutic Innovation, Saint Louis University, St. Louis, MO 63103, USA; 4 Institute for Translational Neuroscience, Saint Louis University, St. Louis, MO 63103, USA

**Keywords:** G protein-coupled receptor, core-hopping, heterocyclic compounds, small molecule, drug discovery, neuropathic pain

## Abstract

G protein-coupled receptor 183 (GPR183, EBI2) has emerged as a promising therapeutic target for neuropathic pain due to its role in neuroinflammatory signaling. Previously, our group identified SAE-14 as a potent GPR183 antagonist and lead scaffold for analgesic development. To further explore the structural requirements for receptor antagonism, a ligand-based core-hopping strategy was employed to identify novel chemotypes derived from SAE-14. Virtual screening identified a trisubstituted benzimidazole scaffold as a promising candidate for further investigation. A focused series of benzimidazole analogs was synthesized and evaluated for inhibition of 7α,25-dihydroxycholesterol (7α,25-OHC)-induced GPR183 activation using calcium mobilization assays. Several compounds exhibited potent antagonistic activity, with four analogs demonstrating comparable potency relative to SAE-14. The most active compounds displayed IC_50_ values of 15.7, 15.8, 18.0, and 19.3 nM. Structure–activity relationship studies revealed that both electron-rich aromatic substituents and heteroaromatic moieties were well tolerated within the benzimidazole scaffold. These findings identify benzimidazole as a novel scaffold for GPR183 antagonism, expand the chemical space surrounding GPR183 inhibitors, and provide a foundation for future lead optimization efforts.

## 1. Introduction

Neuropathic pain is a chronic and debilitating condition affecting approximately 7–10% of the population and is characterized by hyperalgesia, allodynia, paresthesia, and spontaneous pain resulting from injury or dysfunction of the somatosensory nervous system. The prevalence of neuropathic pain continues to increase due to rising rates of diabetes, improved survival of cancer patients receiving neurotoxic chemotherapies, and an aging population [1,2]. Despite the availability of opioids, anticonvulsants, and antidepressants, current treatment options often provide inadequate relief and are associated with significant adverse effects [3]. Consequently, there remains a substantial unmet need for the development of novel non-opioid therapies for chronic pain [4,5].

Neuroinflammation is recognized as a major contributor to the development and maintenance of neuropathic pain, with persistent activation of inflammatory signaling pathways leading to nociceptor sensitization and sustained pain transmission [6]. G protein-coupled receptor 183 (GPR183), also known as Epstein–Barr virus-induced gene 2 (EBI2), has emerged as a promising target within these pathways [7,8]. GPR183 is a Class A G protein-coupled receptor expressed predominantly in lymphoid tissues but also present throughout the central nervous system. The receptor primarily couples to Gα_i_ proteins and regulates several signaling cascades involved in immune function and inflammation, including extracellular signal-regulated kinase (ERK), p38 mitogen-activated protein kinase (MAPK), and nuclear factor-κB (NF-κB) pathways [7,8,9].

**Figure 1:**
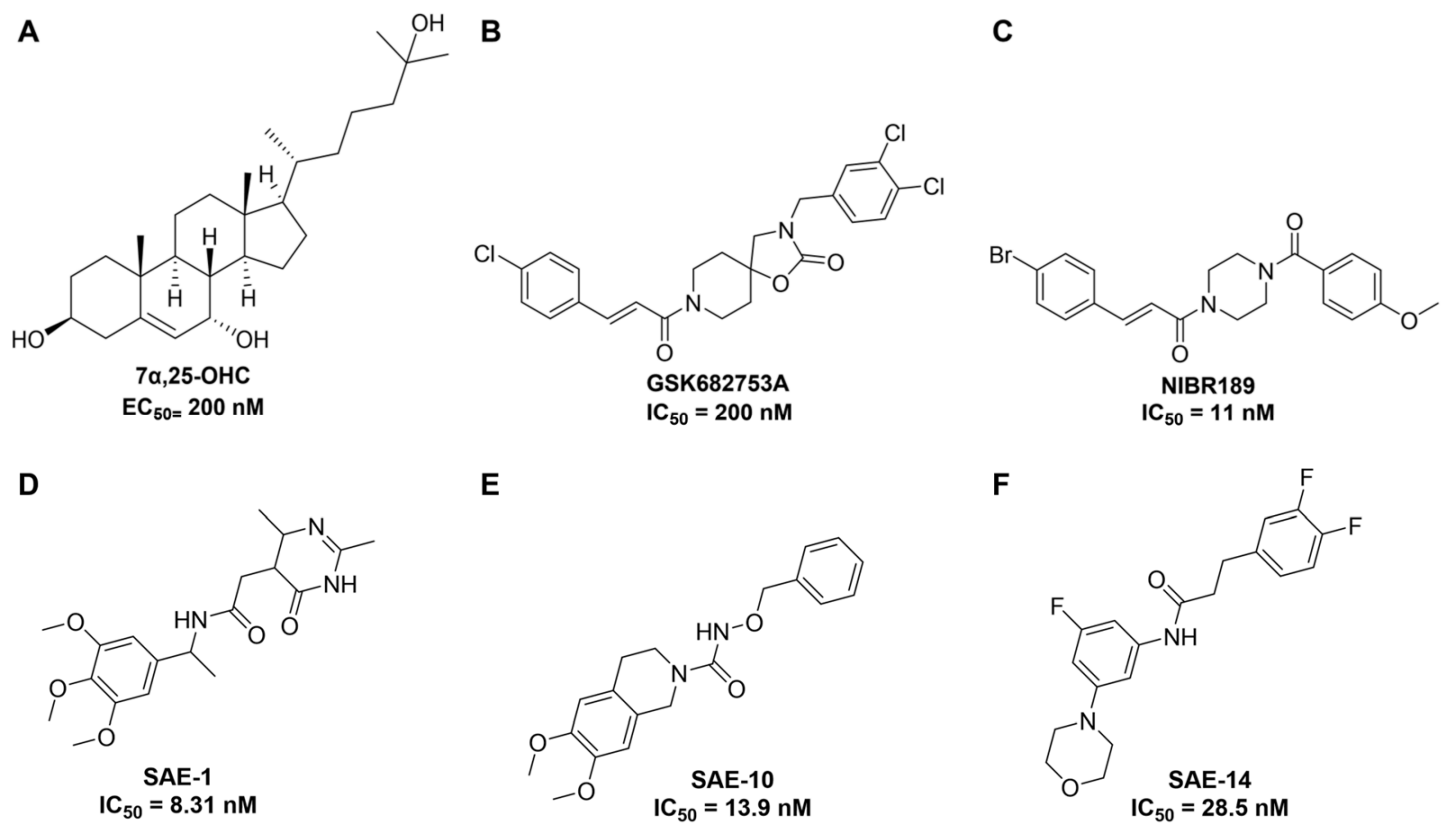
Structures of GPR183 ligands and EC_50_ (**A**) and IC_50_ (**B**–**F**) values.

Activation of these pathways promotes the production of pro-inflammatory mediators associated with neuropathic pain [9,10,11,12,13,14]. Previous studies from our laboratory identified GPR183 as a contributor to neuropathic pain through transcriptomic analysis of chronic constriction injury (CCI) models and demonstrated that activation of the receptor by its endogenous ligand, 7α,25-dihydroxycholesterol (7α,25-OHC, Figure 1A), promotes behavioral hypersensitivity through MAPK- and NF-κB-dependent mechanisms [7,14].

Relatively few small-molecule antagonists of GPR183 have been reported. Early studies identified GSK682753A (Figure 1B) as a potent antagonist [12,13]; however, poor metabolic stability limited its utility for in vivo applications [15]. NIBR189 (Figure 1C) was subsequently reported as a highly potent antagonist with nanomolar activity at both human and mouse GPR183 [16]. Although these compounds established the tractability of GPR183 as a drug target, they also highlighted the limited chemical diversity currently available for receptor modulation. Therefore, there is a need to develop new orally bioavailable small-molecule GPR183 antagonists. To address this limitation, our laboratory previously conducted a structure-based virtual screening campaign that identified three novel antagonists, SAE-1, SAE-10, and SAE-14 (Figure 1D–F). These compounds inhibited 7α,25-OHC-induced receptor activation in vitro, reversed chronic constriction injury (CCI)-induced mechanical allodynia and inhibited 7α,25-OHC-induced mechanical allodynia in vivo [7]. Although SAE-1 exhibited greater in vitro potency (IC_50_ = 8.31 nM), SAE-14 was selected as the template for scaffold hopping based on its overall pharmacological profile and the favorable in vivo efficacy profile that led to its advancement as a lead scaffold in our previous study, rather than on in vitro potency alone [7].

**Figure 2:**
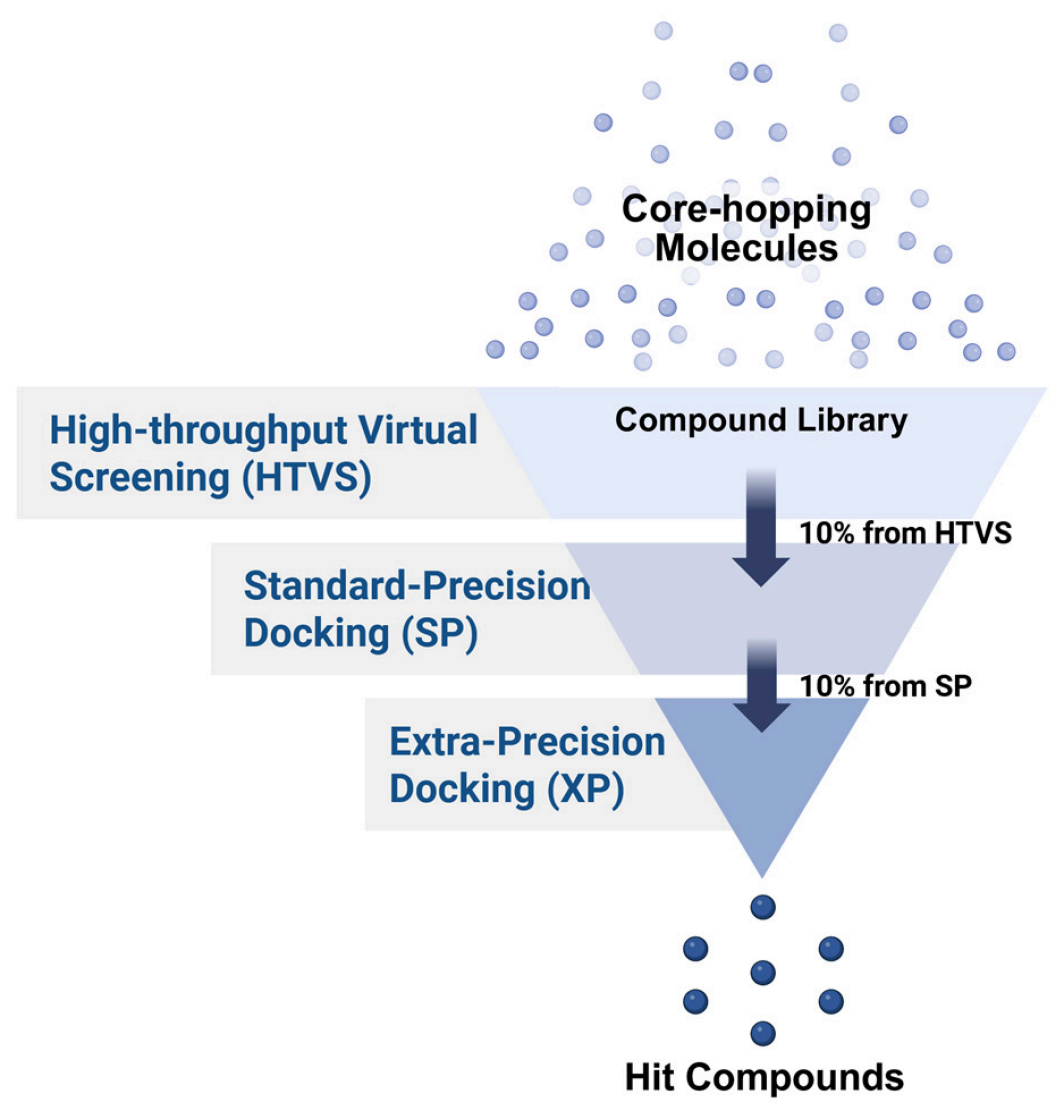
Core-hopping HTVS workflow.

Scaffold hopping is a widely used medicinal chemistry strategy that enables the replacement of a central molecular framework while preserving the key pharmacophoric elements required for biological activity [17,18]. This approach facilitates exploration of novel chemical space, provides opportunities for lead optimization, and can generate structurally differentiated compounds with improved drug-like properties. In the present study, a ligand-based core-hopping workflow was performed using SAE-14 as the template structure to identify novel GPR183 antagonist chemotypes (Figure 2). Virtual screening of the resulting analog library against a cryo-electron microscopy structure of GPR183 [19] identified a trisubstituted benzimidazole scaffold as a promising candidate for further investigation. Herein, we report the design, synthesis, and biological evaluation of a series of benzimidazole-based GPR183 antagonists and describe the resulting structure–activity relationships for this novel chemotype.

## 2. Results and Discussion

### 2.1. Ligand Core-Hopping and Virtual Screening

**Figure 3:**
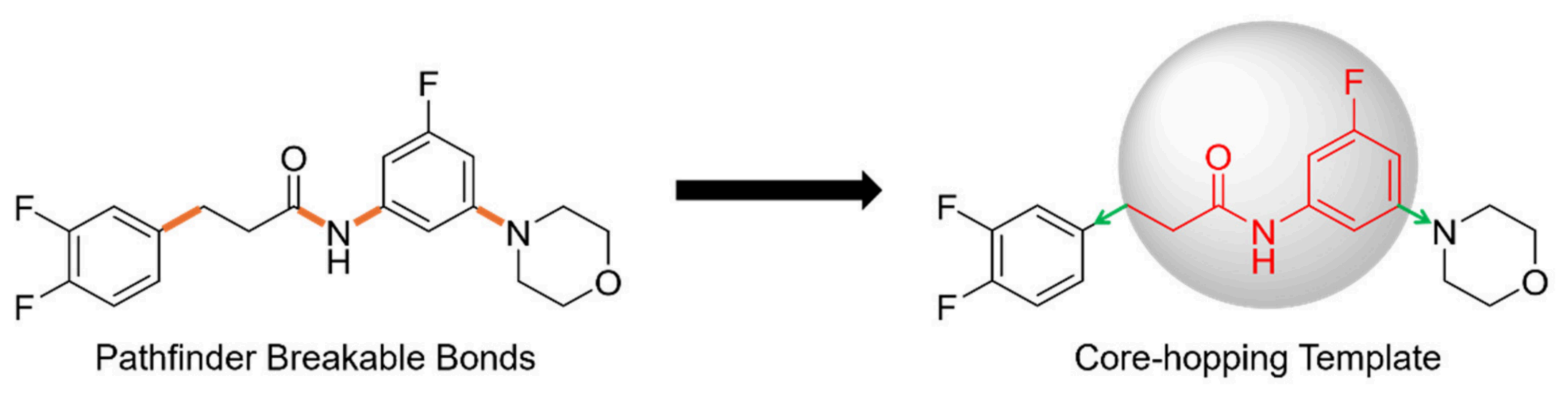
Pathfinder breakable bonds on SAE-14 ((**left**); visualized in orange). SAE-14 growth points used for ligand core-hopping (**right**). Growth points are visualized by green arrows on the beta carbon of the amide linker and the 3 position of the core phenyl ring.

To identify novel GPR183 antagonist chemotypes, a ligand-based core-hopping workflow was performed using SAE-14 as the template structure. Growth points were defined at the β-carbon on the amide linker 1- and 3-positions of the central aniline ring to facilitate replacement of the core while preserving the distal pharmacophoric elements previously shown to be important for receptor antagonism (Figure 3). The resulting core-hopping library was subsequently evaluated using a structure-based virtual screening workflow against a cryo-EM structure of GPR183 [19].

**Figure 4:**
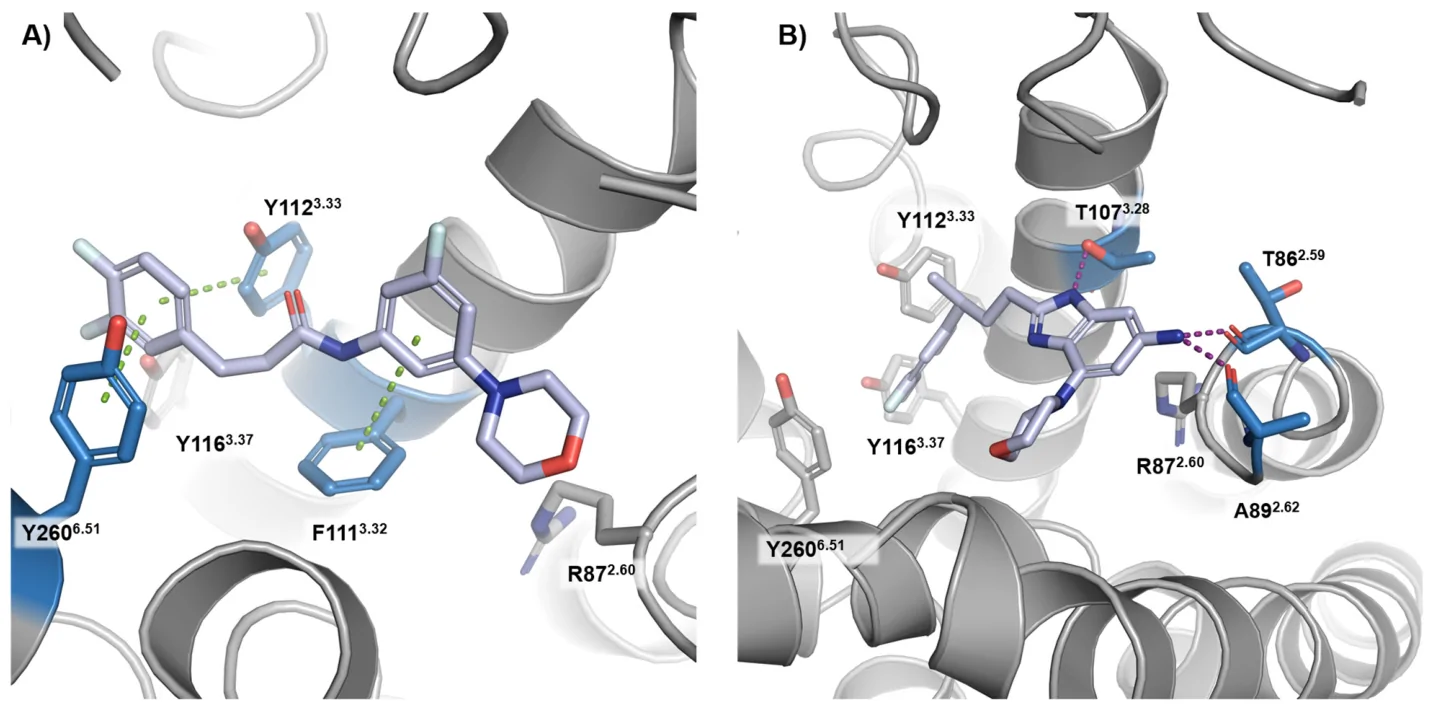
(**A**) SAE-14; (**B**) benzimidazole prototype scaffold molecular interactions. Green = CH-π displaced stacking, pink = hydrogen bond, blue = GPR183 and light purple = the respective ligands. Residues are shown with Ballesteros-Weinstein numbering. Results were visualized in PyMOL 3.1.4.

Candidate scaffolds were prioritized based on predicted binding affinity, maintenance of key receptor interactions, and synthetic accessibility. Among the top-ranked chemotypes, a trisubstituted benzimidazole scaffold emerged as a particularly attractive candidate due to its favorable predicted binding interactions and synthetic tractability. Docking studies suggested that the benzimidazole scaffold occupied a binding region similar to that of SAE-14 while providing additional opportunities to explore receptor interactions through substitution at the C2 position (Figure 4).

**Figure 5:**
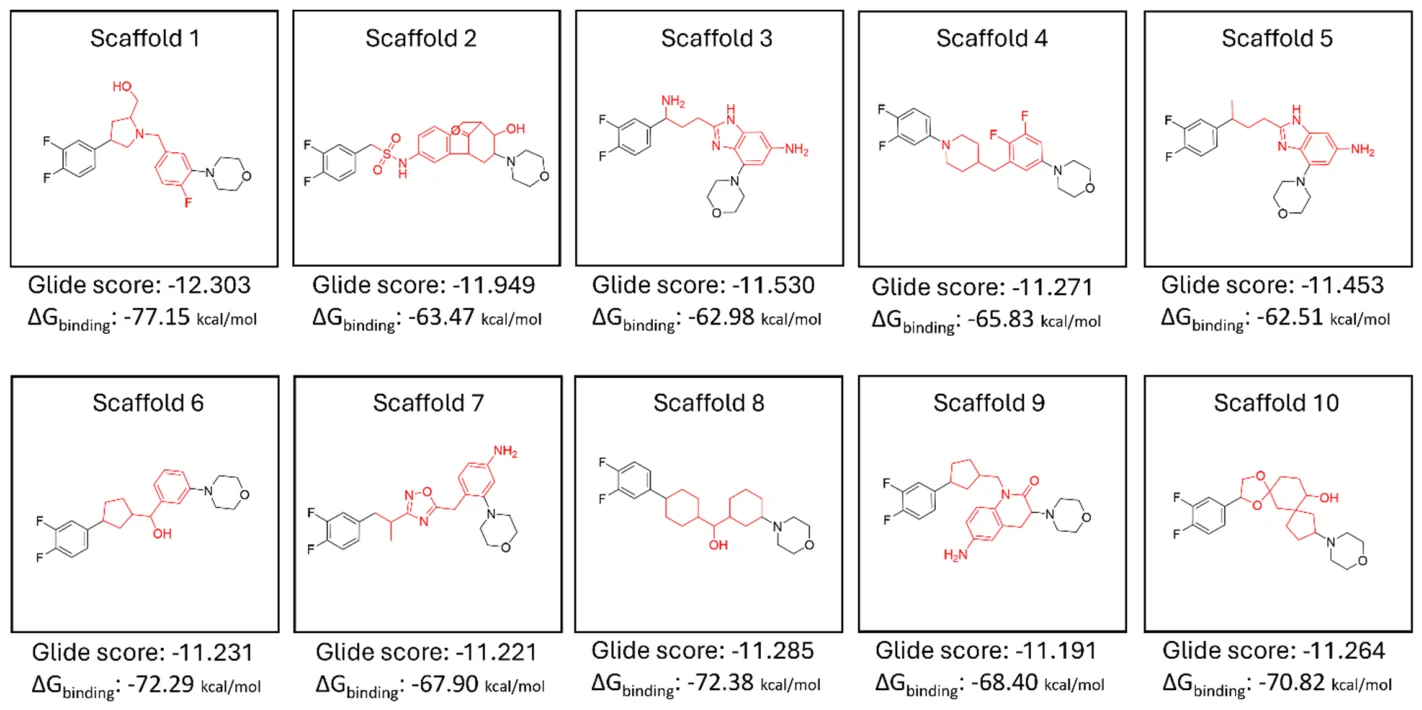
Top ten hit scaffolds produced from the core-hopping VSW along with respective Glide and Prime MM-GBSA scores (ΔG_binding_) expressed in kcal/mol.

Figure 5 shows the top 10 scaffolds resulting from the core-hopping virtual screening workflow (VSW) study. All the scaffold hits proved to be strong candidates based on binding affinity and binding energies. Upon assessing the synthesizability and pharmacokinetic challenges posed by each scaffold, scaffold 2 and scaffold 10 were eliminated from further consideration. The common “drug-like” cores diagnostic of scaffolds 3, 4, 5, and 7 made for attractive lead scaffolds, but ultimately scaffolds 3 and 5 were combined due to their similarity to give a prototype scaffold for the resulting benzimidazole compounds.

**Scheme 1:**
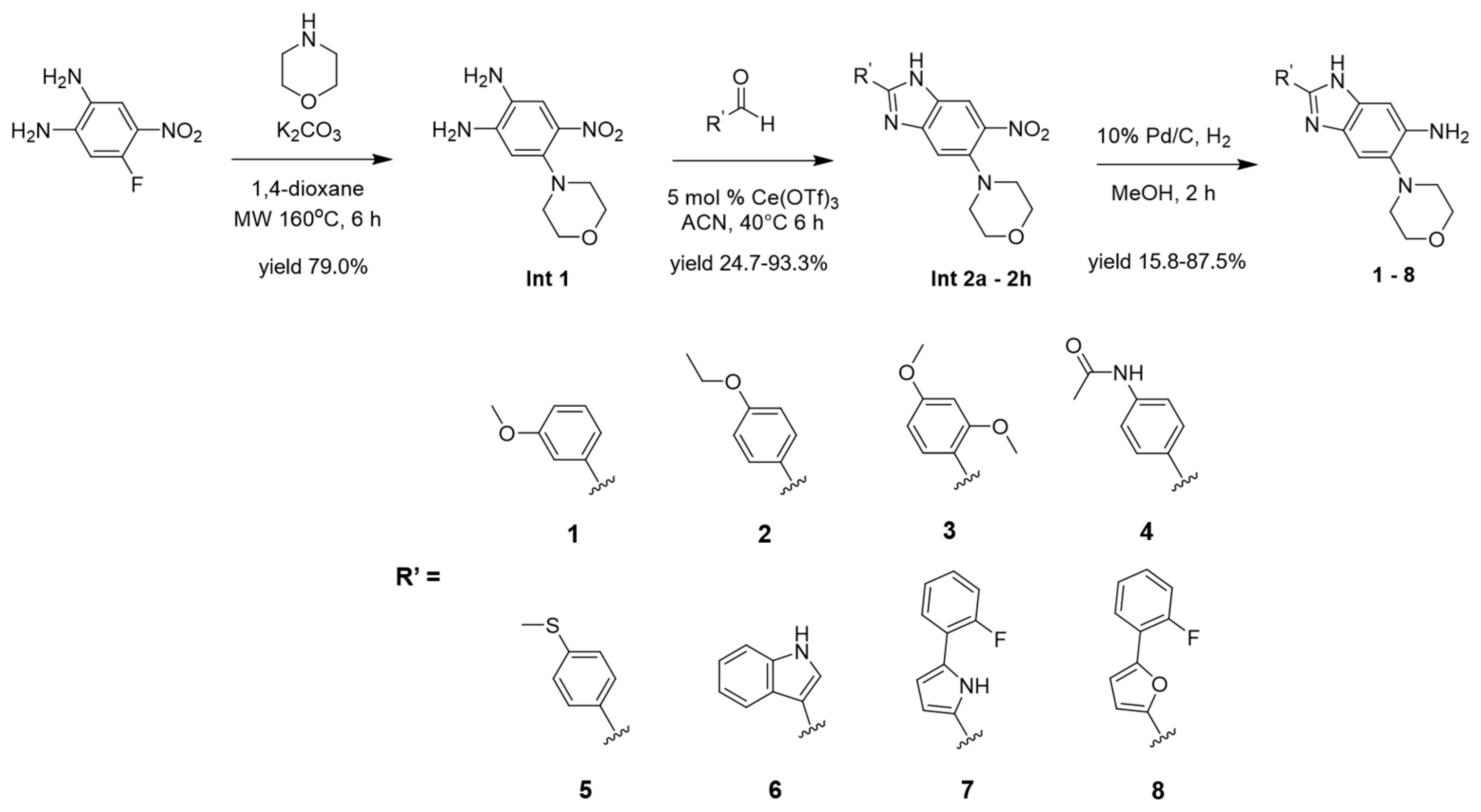
Synthesis pathway for benzimidazole analogs.

The prototype benzimidazole scaffold was optimized by adjusting the morpholine ring to the 2-position rather than the 3-position due to starting material availability and for easier formation of Intermediate 1 (Scheme 1). During synthetic development, the 2-position encountered far fewer synthetic challenges with the nucleophilic aromatic substitution reaction when compared to the 3-position. The scaffold was further modified by shortening the chain linker in favor of aromatic and heterocyclic structures. This shortened length and specific moieties were chosen with consideration of the orthosteric region of the binding pocket. Previous structural studies have identified residues R87^2.60^, Y112^3.33^, Y116^3.37^, and Y260^6.51^ as critical determinants of 7α,25-OHC recognition and receptor activation [20]. The original scaffold hits 3 and 5, while having a favorable docking score and binding energy, did not retain any of these residue interactions essential to binding (Figure 4).

**Figure 6:**
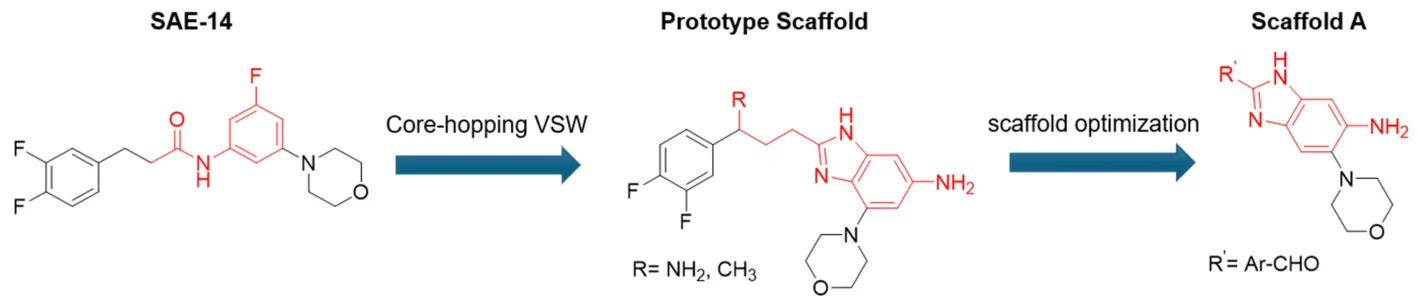
Scaffold creation network from SAE-14 to the prototype scaffold produced by combining scaffolds 3 and 5, and finally Scaffold A, which afforded compounds **1**–**8**.

Consequently, analogue design focused on modifying the substituent projecting into this region of the receptor to explore the structural requirements for antagonism. A series of aromatic and heteroaromatic substituents was manually selected to investigate the influence of steric and electronic effects on receptor activity. The final benzimidazole scaffold, Scaffold A, is shown in Figure 6.

### 2.2. Synthesis

The target benzimidazole analogues were synthesized according to Scheme 1. Nucleophilic aromatic substitution of commercially available 4-fluoro-5-nitrobenzene-1,2-diamine with morpholine afforded Intermediate 1. Condensation of Intermediate 1 with the appropriate aldehydes in the presence of catalytic cerium triflate produced a series of substituted nitrobenzimidazole intermediates. Subsequent catalytic hydrogenation using palladium on carbon afforded the desired amino-benzimidazole derivatives (Compounds **1**–**8**). This synthetic route provided a convenient platform for evaluating structure–activity relationships surrounding the newly identified benzimidazole scaffold.

### 2.3. Biological Evaluation and Structure–Activity Relationships

**Figure 7:**
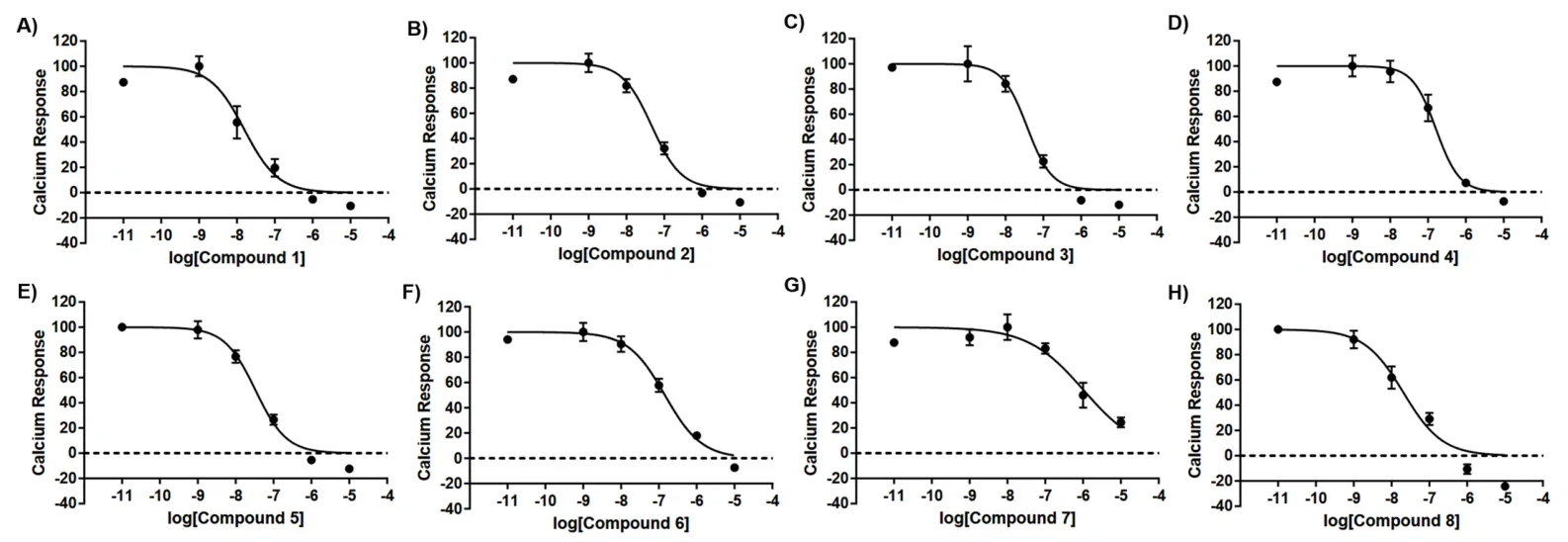
Calcium-mobilization assay dose–response curves for (**A**) Compound **1** (R^2^ = 0.95), (**B**) Compound **2** (R^2^ = 0.97), (**C**) Compound **3** (R^2^ = 0.97), (**D**) Compound **4** (R^2^ = 0.97), (**E**) Compound **5** (R^2^ = 0.97), (**F**) Compound **6** (R^2^ = 0.98), and (**G**) Compound **7** (R^2^ = 0.92), (**H**) Compound **8** (R^2^ = 0.97). Data are presented as mean ± SEM from three independent experiments (n = 3).

For assays, U-937 cells (pro-monocytic, human myeloid leukemia cell line) were used since they endogenously express GPR183 and are well established in the field as a model system to observe GPR183 functional activity [16,21,22,23,24,25,26,27]. Because GPR183 primarily couples to Gαi rather than Gαq [28], U-937 cells were transiently transfected with the previously described chimeric G protein, GαΔ6qi4myr, which redirects Gαi-coupled receptor signaling to a measurable intracellular Ca^2+^ response through phospholipase C-β (PLCβ) [29]. Our previous studies using GPR183-specific siRNA demonstrated that 7α,25-OHC-induced activation and antagonist-mediated inhibition in this assay are dependent on GPR183 expression [7]. Compounds **1**–**8** were therefore evaluated for their ability to inhibit 7α,25-dihydroxycholesterol-induced GPR183 activation using this calcium mobilization assay. Antagonist activity was quantified by determination of IC_50_ values for inhibition of 7α,25-OHC-mediated signaling (Table 1). The benzimidazole scaffold was generally well tolerated, with all analogues exhibiting measurable antagonistic activity (Figure 7A–H). Importantly, several compounds (**1**, **3**, **5**, **8**) demonstrated potency comparable to that of the parent lead SAE-14 (IC_50_ = 28.5 nM) [7], validating the scaffold-hopping approach and demonstrating that replacement of the central aniline core can be achieved without compromising biological activity.

Among the analogues evaluated, the 3-methoxy derivative, compound **1** (Figure 7A), was one of the two most potent compounds in the series, displaying an IC_50_ value of 15.8 nM. The favorable activity observed for this analogue suggests that electron-rich aromatic substituents are well accommodated within the receptor binding pocket. Compound **3** (Figure 7C, IC_50_ = 19.3 nM), containing a 2,4-dimethoxy substitution pattern, also retained potent activity with an IC_50_ value comparable to SAE-14, indicating that multiple methoxy substituents are likely tolerated and may contribute favorably to receptor binding through electronic effects and enhanced aromatic interactions.

Replacement of the methoxy substituent with a 4-methylthio group produced compound **5** (Figure 7E), which afforded the most potent compound in the series (IC_50_ = 15.7 nM). The comparable activity of compounds **1** and **5** suggests that the receptor can accommodate substituents with differing electronic properties, provided that steric requirements are maintained. The sulfur-containing analogue may additionally benefit from increased hydrophobic interactions within the binding pocket.

Compounds **2** and **4**, with 4-ethoxy and 4-acetamido substituents respectively, both maintained nanomolar potency (IC_50_ = 45.7 nM, IC_50_ = 137.3 nM). Compared to more active compounds (**1**, **3**, and **5**), the ethoxy and acetamido moieties are consistent with greater steric strain, suggesting that smaller electron-rich groups in this region of the binding pocket are preferred (Figure 7B,D).

**Figure 8:**
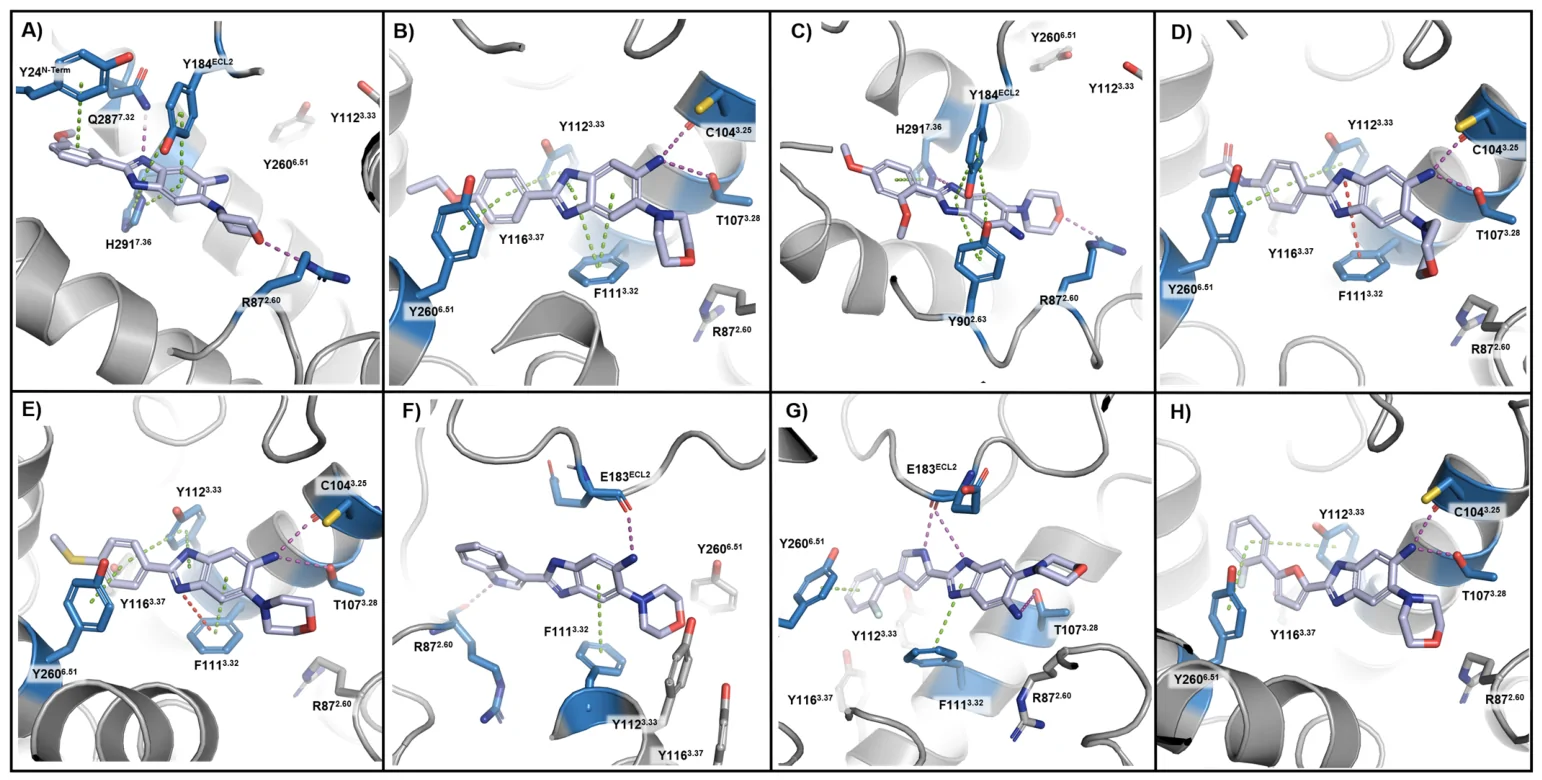
Molecular interactions of compounds **1**–**8** (**A**–**H**) within the receptor. Green = CH-π displaced stacking; Pink = hydrogen bond; Red = π-cation, blue = GPR183 and light purple = the respective ligands. Static poses were chosen based on the lowest docking score, and interactions are represented as an average of interactions over all poses. Residues are shown with Ballesteros-Weinstein numbering. Residues that interact with the ligand are highlighted in blue. Results were visualized in PyMOL 3.1.4.

The heteroaromatic analogues also demonstrated promising activity. Compound **8** (Figure 7H), containing a fluorophenyl-furan substituent, exhibited an IC_50_ value of 18.0 nM. The retention of activity following incorporation of a rigid heteroaromatic system suggests that conformational restriction of the distal aromatic region is well tolerated and may assist in positioning the substituent for productive receptor interactions. However, other heteroaromatic analogues, such as compounds **6** and **7**, displayed reduced activity (IC_50_ = 237.0 nM, IC_50_ = 1211.0 nM), indicating that subtle changes in heteroatom placement and aromatic geometry can significantly influence receptor recognition. It is important to note that the shallow Hill slope for **7** indicates an atypical concentration-response profile and warrants cautious interpretation. Compounds **6** and **7** both have nitrogen-containing substituents in contrast to the oxygen-containing heteroaromatic ring found in compound **8**. The binding modes of these two compounds also differ from receptor interaction trends seen across the compound class (Figure 8F,G). This suggests that proton donors on the C2 position of the benzimidazole scaffold could disrupt residue interactions that are necessary for potency.

The identification of multiple analogues with activity comparable to SAE-14 demonstrates that the benzimidazole scaffold successfully recapitulates the pharmacophoric features required for GPR183 antagonism while providing access to a structurally distinct chemotype. Given the favorable potency observed across several substitution patterns, this scaffold represents a promising platform for future lead optimization efforts directed toward the development of novel GPR183-targeted therapeutics.

### 2.4. Molecular Modeling

To rationalize the observed biological activity, representative compounds were examined using molecular docking studies within the GPR183 binding pocket. The benzimidazole scaffold adopted a binding orientation similar to that predicted for SAE-14 (Figure 5), supporting the hypothesis that scaffold replacement preserved the key pharmacophoric features required for receptor antagonism. Across the series, active compounds were predicted to occupy the orthosteric binding pocket and engage receptor regions previously implicated in recognition of the endogenous ligand 7α,25-OHC.

The trisubstituted benzimidazole scaffold was predicted to support a network of CH-π displaced stacking, π-cation, and hydrogen-bonding interactions that may contribute to receptor recognition. Aromatic interactions involving residues such as Y112^3.33^, Y184^ECL2^, H291^7.36^, and Y260^6.51^ were observed among the most active compounds. Additionally, hydrogen-bonding interactions involving R87^2.60^ and neighboring polar residues, such as C104^3.25^ and T107^3.28^, were also prevalent. (Figure 8). Compound **1** exemplified this interaction pattern (Figure 8A). The benzimidazole core and pendant aromatic substituent on compound **1** were predicted to participate in multiple CH-π interactions with Y184^ECL2^ and H291^7.36^, while the morpholine oxygen maintained a hydrogen bond with R87^2.60^ (Figure 8A). Additional interactions involving the benzimidazole nitrogen and Q287^7.32^ further supported ligand binding.

Compound **3** displayed a similar interaction profile and retained many of the contacts observed for compound **1**, though with some notable differences (Figure 8C). Compound **3** shared most of the same residue contacts predicted for compound **1**, such as Y184^ECL2^, H291^7.36^, and R87^2.60^. However, compound **3** was predicted to participate in multiple CH-π interactions with Y90^2.63^ on the benzimidazole core, whereas compound **1** was predicted to interact with Q287^7.32^ and Y24^N-term^ on the core and aromatic substituent, respectively.

Compounds **2**, **4**, **5**, and **8** shared many of the same predicted interaction motifs, and an interaction pattern can be seen among these ligands (Figure 8B,D,E,H). Compounds **2**, **4**, **5**, and **8** were predicted to engage Y112^3.33^ and Y260^6.51^ through aromatic interactions while maintaining hydrogen-bonding contacts between the amino group and residues C104^3.25^ and T107^3.28^. Numerous interactions were also predicted between F111^3.32^ and the benzimidazole core. Although the fluorophenyl-furan substituent in compound **8** altered the positioning of the distal aromatic region, the aromatic interactions with Y112^3.33^ and Y260^6.51^ and hydrogen-bonding contacts with C104^3.25^ and T107^3.28^ were preserved. These conserved interactions may explain the comparable potent antagonistic activity observed experimentally across the series despite variation in the terminal aromatic substituent. This suggests that the receptor can accommodate structurally diverse aromatic substituents provided that key binding interactions are retained.

The heteroaromatic compound **8** also retained favorable interactions within the binding pocket. Although the fluorophenyl-furan substituent altered the positioning of the distal aromatic region, aromatic interactions with Y112^3.33^ and Y260^6.51^ were preserved (Figure 8H). The maintenance of these contacts is consistent with the potent antagonistic activity observed experimentally and suggests that the receptor can accommodate structurally diverse aromatic substituents provided that key binding interactions are retained. Dose–response curves for compounds **2**, **4**, **6**, and **7** can be seen in Figure 7.

The binding interactions of the other heteroaromatic compounds (**6**, **7**) differ from those of compound **8** (Figure 8F,G). Both compounds **6** and **7** also altered the distal aromatic region, which resulted in a predicted loss of key residue contacts. The amino group on compound **6** and heteroaromatic nitrogens on compound **7** were predicted to instead exhibit hydrogen-bonding interactions with E183^ECL2^. The predicted loss of essential residue interactions could be linked to the observed loss in potency seen experimentally. In contrast to compound **1**, compound **7** did not retain the same network of predicted interactions with Y184^ECL2^, H291^7.36^, R87^2.60^, and Q287^7.32^. Instead, the altered orientation of the heteroaromatic substituent in compound **7** was associated with loss of several of these contacts and a predicted hydrogen-bonding interaction with E183^ECL2^. This change in the predicted interaction profile may contribute to the substantially lower antagonistic potency observed for compound **7**.

The aromatic moieties facilitated many more interactions when compared to the original prototype scaffold hit (Figure 5), supporting the idea that potent antagonism is strongly dependent on the nature of the substituent. Substitution of the 3,4-difluoro chain linker in the original scaffold hit with shorter, electron-rich aromatic substituents increased interactions with key residues. Additionally, SAR revealed that subtle changes in position, size, and atom identity could substantially affect binding interactions. Within heteroaromatic substituents, the positioning and identity of heteroatoms may dramatically influence binding affinity. Overall, the docking data suggest that GPR183 favors aromatic moieties capable of occupying the orthosteric region of the binding pocket [20,25,26] without introducing excessive steric bulk or disrupting key receptor interactions.

Collectively, the docking studies support the structure–activity relationship trends observed experimentally and suggest that the benzimidazole scaffold successfully preserves the critical receptor interactions required for potent GPR183 antagonism. These findings further validate the scaffold-hopping strategy and establish the benzimidazole chemotype as a promising platform for future lead optimization efforts.

### 2.5. Conclusions

A ligand-based core-hopping strategy was successfully employed to identify a novel benzimidazole scaffold for GPR183 antagonism. Virtual screening of the previously reported antagonist SAE-14, followed by synthesis and biological evaluation of a focused analogue series, demonstrated that the benzimidazole core effectively preserves the pharmacophoric features required for potent inhibition of GPR183. Several analogues exhibited low nanomolar activity, with compounds **1**, **3**, **5**, and **8** displaying comparable potency relative to SAE-14. Structure–activity relationship studies revealed that both electron-rich aromatic substituents and heteroaromatic motifs are well tolerated within the scaffold, providing valuable insight into the structural requirements governing receptor antagonism. Collectively, these findings establish benzimidazole as a promising new chemotype for GPR183 inhibition and further validate scaffold hopping as an effective approach for expanding the chemical space surrounding GPR183 antagonists. Future studies on the ADME/Tox properties of the benzimidazole chemotype are necessary to develop understanding of the class as potential therapeutics. The compounds described herein provide a foundation for future optimization efforts aimed at improving molecular properties and advancing novel non-opioid therapeutics for the treatment of neuropathic pain.

## 3. Materials and Methods

### 3.1. Materials

4-fluoro-5-nitrobenzene-1,2-diamine and 4-(3,4-difluorophenyl)butanoic acid were purchased from Aaron Chemical (San Diego, CA, USA). 3-methoxybenzaldehyde, (*1H*)-Indole-3-carbaldehyde, 5-(2-fluorophenyl)furan-carbaldehyde, and 5-(2-fluorophenyl)pyrrole-carbaldehyde were purchased from Oakwood Chemical (Estill, SC, USA). Cerium trifluoromethanesulfonate, 2,4-dimethoxy-, 4-ethoxy-, 4-(methylthio)-, 4-acetamido-benzaldehydes, and all other solvents were purchased from Sigma-Aldrich (St. Louis, MO, USA) and were used as received without further purification. 7-α,25-Dihydroxycholesterol was purchased from MedChemExpress (Monmouth Junction, NJ, USA).

### 3.2. Computational Methods

All molecular modeling studies were performed using the Schrödinger Maestro molecular modeling suite (Maestro 14.4, Schrödinger LLC, New York, NY, USA). A cryo-electron microscopy structure of human GPR183 (PDB ID: 7TUY) [19] was prepared using the Protein Preparation Wizard within Schrödinger Maestro. Chain L and non-essential crystallographic components were removed, missing residues were modeled using Prime, and the resulting structure was subjected to restrained minimization using the OPLS4 force field. The previously reported GPR183 antagonist SAE-14 was prepared using LigPrep and docked into the receptor binding pocket to generate the reference complex used for subsequent studies. The Pathfinder suite was used to examine the breakable bonds on SAE-14, which informed the location of the growth points for the core-hopping template. Growth points were defined at the β-carbon on the amide linker and the 3-position of the central aniline core ring to facilitate scaffold replacement while preserving the distal pharmacophoric elements (Figure 3). Ligand-based core hopping was performed using the Ligand-Based core-hopping module within Schrödinger (New York, NY, USA), employing the 2023 Enamine Building Blocks library (25.5 k compounds) as the source of replacement scaffolds. Output structures generated from the core-hopping workflow (10,000 compounds) were subject to virtual screening using the Virtual Screening Workflow (VSW) (Figure 2). First, all 10,000 compounds were prepared using LigPrep and used in a high-throughput Grid-based ligand docking and energetics (GLIDE) docking (HTVS, flexible docking), with 1 pose generated per compound state. All state penalties were generated by Epik with a target pH of 7.0 ± 2.0. The top 1000 compounds (10%) with the lowest GlideScores (computational estimate of binding) were subject to standard precision docking (GLIDE SP) using flexible ligand sampling in GLIDE with the default settings. Next, the top 100 compounds (10%) with the lowest GlideScores were subject to extra precision docking (GLIDE XP) using flexible ligand sampling in GLIDE with the default settings. Post-docking minimization was performed after each docking stage, and final hits were post-processed through Prime MM-GBSA (see Supplemental Table S1). Based on this final docking, the top 10 compounds were prioritized based on docking score, predicted binding mode, synthetic accessibility, and Prime MM-GBSA binding energy calculations (Figure 4). After optimization, the selected benzimidazole scaffold (Scaffold A, Figure 6) was advanced for synthesis and biological evaluation. Flexible GLIDE SP docking was performed on prepared structures of compounds **1**–**8** using the default settings with 3 poses generated per ligand.

### 3.3. Chemistry

General Experimental Procedures: All reagents and solvents were purchased from commercial suppliers and used without further purification unless otherwise noted. Reaction progress was monitored by thin-layer chromatography (TLC) on silica gel plates, visualized under UV light. Flash chromatography was performed using a Teledyne ISCO CombiFlash^®^ NextGen 100 system (Lincoln, NE, USA) equipped with C18 reverse-phase columns. Microwave-assisted reactions were conducted using a Biotage^®^ Initiator+ Robot Sixty microwave reactor (Charlotte, NC, USA). Nuclear magnetic resonance (NMR) spectra were recorded on a Bruker 400 MHz spectrometer (Billerica, MA, USA). Chemical shifts (δ) are reported in parts per million (ppm) and referenced to residual solvent signals. High-resolution mass spectra (HRMS) were obtained using electrospray ionization (ESI) methods. The purity of all final amino-benzimidazole compounds was confirmed to be ≥95% by analytical HPLC before biological evaluation.

Synthesis of 4-Nitro-5-(4-morpholinyl)-1,2-benzenediamine (Intermediate 1): 4-Fluoro-5-nitrobenzene-1,2-diamine (5.84 mmol), morpholine (11.68 mmol), and potassium carbonate (17.52 mmol) were dissolved in 1,4-dioxane (10 mL) in a sealed microwave reaction vessel. The reaction mixture was heated at 170 °C for 3 h under microwave irradiation. After cooling to room temperature, the reaction mixture was filtered to remove inorganic salts and extracted with ethyl acetate and water. The organic layer was concentrated under reduced pressure to afford Intermediate 1 as a red solid.

Int 1: 4-Nitro-5-(4-morpholinyl)-1,2-benzenediamine. Red solid, yield 79.0%. ^1^H NMR (400 MHz, CDCl_3_) δ 7.55 (s, 1H), 6.36 (s, 1H), 4.12 (s, 2H), 3.88–3.85 (m, 4H), 3.21 (s, 2H), 3.02–2.97 (m, 4H). ^13^C NMR (101 MHz, CDCl_3_) δ 143.62, 135.36, 125.18, 116.48, 105.87, 67.26, 53.07, 32.35, 26.54. Purity 96.3% HRMS (ESI): calculated for C_10_H_14_N_4_O_3_ [M+H]^+^ 239.1099, found 239.1127.

General Procedure for the Synthesis of Nitro-Benzimidazole Intermediates: Intermediate 1 (0.105 mmol), the appropriate aldehyde (0.105 mmol), and cerium trifluoromethanesulfonate (5 mol%) were dissolved in acetonitrile and stirred at 40 °C for 6 h. Upon completion, the reaction mixture was purified by reverse-phase flash chromatography using a water (0.1% formic acid)/acetonitrile gradient to afford the corresponding nitro-benzimidazole intermediates.

Int 2a: 2-(m-methoxyphenyl)-5-morpholino-6-nitro-1*H*-1,3-benzimidazole. Orange solid, yield 72.5%. ^1^H NMR (400 MHz, CDCl_3_) δ 8.26 (s, 1H), 7.98 (s, 1H), 7.63 (s, 1H), 7.45 (t, *J* = 7.9 Hz, 1H), 7.08 (d, *J* = 8.2 Hz, 1H), 3.92 (s, 3H), 3.90–3.84 (m, 4H), 3.11–3.00 (m, 4H). Purity 95.4%. HRMS (ESI): calculated for C_18_H_18_N_4_O_4_ [M+H]^+^ 355.1362, found 355.1389.

Int 2b: 2-(*p*-ethoxyphenyl)-5-morpholino-6-nitro-1*H*-1,3-benzimidazole. Orange solid, yield 63.0%. ^1^H NMR (400 MHz, CDCl_3_) δ 8.22 (s, 1H), 7.95 (d, *J* = 11.0 Hz, 2H), 7.59 (s, 1H), 7.04 (d, *J* = 8.5 Hz, 2H), 4.13 (q, *J* = 7.0 Hz, 2H), 3.94–3.82 (m, 4H), 3.13–2.99 (m, 4H), 1.47 (t, *J* = 7.0 Hz, 3H). Purity 95.3%. HRMS (ESI): calculated for C_19_H_20_N_4_O_4_ [M+H]^+^ 369.1518, found 369.1546.

Int 2c: 2-(2,4-dimethoxyphenyl)-5-morpholino-6-nitro-1*H*-1,3-benzimidazole. Orange solid, yield 60.0%. ^1^H NMR (400 MHz, CDCl_3_) δ 10.31–10.26 (m, 1H), 7.82 (d, *J* = 8.7 Hz, 1H), 6.55 (dd, *J* = 8.7, 2.2 Hz, 1H), 6.45 (d, *J* = 2.3 Hz, 1H), 4.11 (dd, *J* = 12.0, 6.4 Hz, 1H), 3.93–3.64 (m, 10H), 3.10–3.01 (m, 1H). Purity 90.8%. HRMS (ESI): calculated for C_19_H_20_N_4_O_5_ [M+H]^+^ 385.1467, found 385.1486.

Int 2d: *N*-[*p*-(5-morpholino-6-nitro-1*H*-1,3-benzimidazol-2-yl)phenyl]acetamide. Orange solid, yield 24.7%. ^1^H NMR (400 MHz, CDCl_3_) δ 8.55 (s, 1H), 8.00 (d, *J* = 6.2 Hz, 1H), 7.87 (d, *J* = 8.5 Hz, 1H), 7.71 (s, 1H), 7.64 (d, *J* = 8.2 Hz, 1H), 7.52 (s, 1H), 7.00 (s, 1H), 6.30 (s, 1H), 4.97 (s, 1H), 3.96–3.81 (m, 4H), 3.16–2.97 (m, 4H), 2.35 (t, *J* = 6.8 Hz, 1H), 2.24 (d, *J* = 6.9 Hz, 3H). Purity 90.3% HRMS (ESI): calculated for C_19_H_19_N_5_O_4_ [M+H]^+^ 382.1471, found 382.1493.

Int 2e: 2-[*p*-(methylthio)phenyl]-5-morpholino-6-nitro-1*H*-1,3-benzimidazole. Orange solid, yield 87.5%. 1H NMR (400 MHz, CDCl3) δ 8.23 (s, 1H), 7.99–7.90 (m, 2H), 7.61 (s, 1H), 7.37 (d, *J* = 7.9 Hz, 2H), 3.93–3.82 (m, 4H), 3.07 (d, *J* = 5.4 Hz, 4H), 2.56 (s, 3H). Purity 98.7%. HRMS (ESI): calculated for C_18_H_18_N_4_O_3_S [M+H]^+^ 371.1133, found 371.1161.

Int 2f: 2-(3-indolyl)-5-morpholino-6-nitro-1*H*-1,3-benzimidazole. Orange solid, yield 93.3% ^1^H NMR (400 MHz, CDCl_3_) δ 8.79 (s, 1H), 8.01 (d, *J* = 2.4 Hz, 1H), 7.85 (d, *J* = 3.1 Hz, 1H), 7.71 (d, *J* = 2.9 Hz, 1H), 7.55 (s, 1H), 7.51 (d, *J* = 3.3 Hz, 1H), 7.45 (dd, *J* = 8.1, 6.1 Hz, 1H), 7.38–7.32 (m, 2H), 6.35 (d, *J* = 9.5 Hz, 1H), 3.93–3.85 (m, 6H), 3.09 (t, *J* = 4.7 Hz, 4H), 3.01–2.97 (m, 2H). Purity 96.4% HRMS (ESI): calculated for C_19_H_17_N_5_O_3_ [M+H]^+^ 364.1365, found 364.1393.

Int 2g: 2-[5-(*o*-fluorophenyl)-2-pyrrolyl]-5-morpholino-6-nitro-1*H*-1,3-benzimidazole. Orange solid, yield 26.5%. ^1^H NMR (400 MHz, CDCl_3_) δ 7.93 (s, 1H), 7.65 (s, 1H), 7.59–7.50 (m, 2H), 7.17–7.08 (m, 4H), 6.71 (s, 1H), 6.52 (s, 1H), 5.50 (d, *J* = 5.5 Hz, 2H), 3.88 (d, *J* = 4.2 Hz, 4H), 3.11–2.99 (m, 4H). Purity 98.7%. HRMS (ESI): calculated for C_21_H_18_FN_5_O_3_ [M+H]^+^ 408.1427, found 408.1456.

Int 2h: 2-[5-(*o*-fluorophenyl)-2-furyl]-5-morpholino-6-nitro-1*H*-1,3-benzimidazole. Orange solid, yield 58.0%. ^1^H NMR (400 MHz, CDCl_3_) δ 8.22 (s, 1H), 8.01 (s, 1H), 7.97–7.89 (m, 1H), 7.42 (d, *J* = 3.7 Hz, 1H), 7.37 (d, *J* = 3.6 Hz, 1H), 7.22–7.16 (m, 1H), 7.08–7.04 (m, 1H), 3.93–3.84 (m, 4H), 3.07 (t, *J* = 4.2 Hz, 4H). Purity 96.7%. HRMS (ESI): calculated for C_21_H_17_FN_4_O_4_ [M+H]^+^ 409.1267, found 409.1282.

General Procedure for the Synthesis of Amino-Benzimidazole Analogues (Compounds **1**–**8**): The appropriate nitro-benzimidazole intermediate (Int 2a–2h) was dissolved in methanol and treated with 10% palladium on carbon under a hydrogen atmosphere. The reaction mixture was stirred at room temperature until complete consumption of the starting material, as determined by TLC. The catalyst was removed by filtration through Celite^®^, and the filtrate was concentrated under reduced pressure. The final products were purified by preparative reverse-phase HPLC to afford the desired amino-benzimidazole derivatives. Full characterization data for all intermediates and final compounds are provided in the Supporting Information.

Compound **1**: 2-(m-methoxyphenyl)-5-morpholino-1*H*-1,3-benzimidazol-6-ylamine. Yellow solid, yield 19.7%. ^1^H NMR (400 MHz, CDCl_3_) δ 8.57 (s, 1H), 7.57 (dt, *J* = 8.7, 2.2 Hz, 1H), 7.47 (t, *J* = 7.2 Hz, 1H), 7.29 (s, 1H), 7.08 (d, *J* = 6.9 Hz, 1H), 6.90–6.80 (m, 2H), 3.92–3.85 (m, 8H), 3.15 (s, 2H), 2.95 (t, *J* = 4.6 Hz, 4H), 2.86 (s, 3H). ^13^C NMR (101 MHz, CDCl_3_) δ 160.04, 129.99, 118.24, 116.81, 110.76, 67.71, 55.46, 52.25, 50.81, 42.77. Purity: 98.2%. HRMS (ESI): calculated for C_18_H_20_N_4_O_2_ [M+H]^+^ 325.1620, found 325.1646.

Compound **2**: 2-(*p*-ethoxyphenyl)-5-morpholino-1*H*-1,3-benzimidazol-6-ylamine. Yellow solid, yield 15.8%. ^1^H NMR (400 MHz, CDCl_3_) δ 8.85 (s, 1H), 7.88 (t, *J* = 9.0 Hz, 2H), 7.18 (s, 1H), 7.07 (d, *J* = 14.1 Hz, 1H), 6.89–6.77 (m, 3H), 4.00 (q, *J* = 6.9 Hz, 3H), 3.86 (s, 4H), 3.11 (s, 2H), 2.90 (t, *J* = 4.7 Hz, 4H), 2.83 (s, 3H). ^13^C NMR (101 MHz, CDCl_3_) δ 168.55, 160.98, 139.87, 138.12, 128.31, 114.83, 114.81, 105.90, 67.60, 63.57, 52.02, 50.68, 42.64. Purity: 97.8%. HRMS (ESI): calculated for C_19_H_22_N_4_O_2_ [M+H]^+^ 339.1776, found 339.1803.

Compound **3**: 2-(2,4-dimethoxyphenyl)-5-morpholino-1*H*-1,3-benzimidazol-6-ylamine. Yellow solid, yield 33.3%. ^1^H NMR (400 MHz, CDCl_3_) δ 8.44 (d, *J* = 8.8 Hz, 1H), 7.35 (s, 1H), 6.94 (s, 1H), 6.68 (dd, *J* = 8.8, 2.3 Hz, 1H), 6.58 (d, *J* = 2.3 Hz, 1H), 4.05 (s, 3H), 3.91–3.86 (m, 7H), 2.97 (t, *J* = 4.6 Hz, 4H). ^13^C NMR (101 MHz, CDCl_3_) δ 131.09, 106.15, 99.14, 83.56, 67.93, 56.14, 55.74, 52.56. Purity 95.9% HRMS (ESI): calculated for C_19_H_22_N_4_O_3_ [M+H]^+^ 355.1725, found 355.1751.

Compound **4**: *N*-[*p*-(6-amino-5-morpholino-1*H*-1,3-benzimidazol-2-yl)phenyl]acetamide. Yellow solid, yield 25.0%. ^1^H NMR (400 MHz, MeOD) δ 7.85 (d, *J* = 8.8 Hz, 2H), 7.66 (d, *J* = 8.9 Hz, 2H), 7.29 (d, *J* = 8.5 Hz, 1H), 7.18 (s, 1H), 7.00 (d, *J* = 8.5 Hz, 1H), 6.90 (d, *J* = 8.1 Hz, 1H), 3.78 (t, *J* = 4.5 Hz, 7H), 2.89–2.77 (m, 6H), 2.18 (s, 2H), 2.07 (s, 3H). ^13^C NMR (101 MHz, MeOD) δ 149.07, 140.82, 140.21, 138.42, 135.86, 133.76, 133.49, 128.80, 126.74, 119.92, 119.71, 105.71, 97.83, 67.24, 67.20, 52.56, 52.12, 22.59, 22.30, 19.48. Purity: 95.0%. HRMS (ESI): calculated for C_19_H_21_N_5_O_2_ [M+H]^+^ 352.1729, found 352.1755.

Compound **5**: 2-[*p*-(methylthio)phenyl]-5-morpholino-1*H*-1,3-benzimidazol-6-ylamine. Yellow solid, yield 75.0%. ^1^H NMR (400 MHz, CDCl_3_) δ 8.74 (s, 1H), 7.75 (d, *J* = 8.0 Hz, 2H), 7.06 (d, *J* = 6.3 Hz, 3H), 6.73 (s, 1H), 3.86 (t, *J* = 4.4 Hz, 4H), 2.87 (t, *J* = 4.6 Hz, 4H), 2.45 (s, 3H). ^13^C NMR (101 MHz, CDCl_3_) δ 168.19, 147.15, 143.04, 140.28, 138.60, 126.68, 125.55, 105.63, 97.54, 67.58, 51.96, 50.89, 14.82. Purity: 96.7%. HRMS (ESI): calculated for C_18_H_20_N_4_OS [M+H]^+^ 341.1391, found 341.1417.

Compound **6**: 2-(3-indolyl)-5-morpholino-1*H*-1,3-benzimidazol-6-ylamine. Yellow solid, yield 87.5%. ^1^H NMR (400 MHz, CDCl_3_) δ 8.65 (s, 1H), 8.23–8.16 (m, 1H), 7.80 (d, *J* = 2.5 Hz, 1H), 7.48–7.41 (m, 1H), 7.32–7.27 (m, 2H), 6.91 (s, 1H), 4.12 (q, *J* = 7.1 Hz, 1H), 3.88 (s, 4H), 3.40–3.29 (m, 1H), 2.95 (dt, *J* = 14.3, 6.6 Hz, 4H). ^13^C NMR (101 MHz, CDCl_3_) δ 153.42, 153.04, 142.92, 142.73, 141.55, 140.64, 139.62, 138.76, 130.34, 129.97, 129.92, 127.02, 126.01, 125.18, 125.14, 116.67, 112.09, 111.79, 102.87, 72.39, 72.05, 57.77, 57.38, 57.20. Purity 97.2%. HRMS (ESI): calculated for C_19_H_19_N_5_O [M+H]^+^ 334.1623, found 334.1653.

Compound **7**: 2-[5-(*o*-fluorophenyl)-2-pyrrolyl]-5-morpholino-1*H*-1,3-benzimidazol-6-ylamine. Light yellow solid, yield 50.0%. ^1^H NMR (400 MHz, CDCl_3_) δ 7.69–7.50 (m, 2H), 7.45 (d, *J* = 11.1 Hz, 1H), 7.10 (d, *J* = 14.1 Hz, 4H), 6.70 (s, 1H), 6.62–6.47 (m, 2H), 3.82 (d, *J* = 17.9 Hz, 4H), 2.97–2.78 (m, 4H). ^13^C NMR (101 MHz, CDCl_3_) δ 157.35, 127.94, 126.86, 124.73, 116.30, 106.45, 95.51, 67.42, 53.59, 51.77, 32.41. Purity 98.1% HRMS (ESI): calculated for C_21_H_20_FN_5_O [M+H]^+^ 378.1685, found 378.1712.

Compound **8**: 2-[5-(*o*-fluorophenyl)-2-furyl]-5-morpholino-1*H*-1,3-benzimidazol-6-ylamine. Yellow solid, yield 80.0%. ^1^H NMR (400 MHz, CDCl_3_) δ 8.37 (s, 1H), 7.95 (td, *J* = 7.6, 1.9 Hz, 1H), 7.34–7.29 (m, 1H), 7.25–7.21 (m, 1H), 7.14 (ddd, *J* = 11.4, 8.0, 1.3 Hz, 1H), 6.86 (t, *J* = 3.5 Hz, 1H), 6.83 (s, 1H), 3.91–3.80 (m, 4H), 2.91 (t, *J* = 4.5 Hz, 4H). ^13^C NMR (101 MHz, CDCl_3_) δ 160.02, 157.52, 148.95, 148.92, 144.78, 142.45, 139.38, 137.70, 128.93, 128.85, 126.09, 126.07, 124.24, 124.21, 118.25, 118.13, 116.02, 115.81, 112.64, 112.52, 112.02, 107.76, 98.22, 67.68, 52.29. Purity 99.8% HRMS (ESI): calculated for C_21_H_19_FN_4_O_2_ [M+H]^+^ 379.1562, found 379.1547.

### 3.4. Cell Culture and Transfection

Human U-937 cells were maintained in RPMI-1640 medium supplemented with 10% fetal bovine serum, 1% penicillin-streptomycin, and 1% GlutaMAX™ at 37 °C in a humidified incubator containing 5% CO_2_. Cells (1 × 10^7^ cells total) were transiently transfected with the promiscuous chimeric G protein GαΔ6qi4myr [29] using polyethyleneimine (PEI) transfection reagent. The chimeric G protein gene vector, GαΔ6qi4myr, was manufactured by VectorBuilder (VB220414-1268cnf, VectorBuilder, Chicago, IL, USA) using the sequence previously described [29]. Briefly, plasmid DNA was incubated with PEI in Opti-MEM^®^ reduced-serum medium for 30 min at room temperature before addition to the cell suspension. Cells were incubated for 48 h before use in calcium mobilization assays.

### 3.5. Calcium Mobilization Assay

Compounds were screened for their potency using a calcium mobilization assay, as previously published [14]. Intracellular calcium mobilization was measured using an Indo-1 AM fluorescence assay. Test compounds were prepared as 10 mM stock solutions in DMSO and serially diluted in assay buffer consisting of HBSS supplemented with HEPES (50:1 HBSS:HEPES) and 0.1% bovine serum albumin. U-937 cells expressing GαΔ6qi4myr [29] were loaded with Indo-1 AM dye (5 µM) (AAT Bioquest, Cat No. 21030, Pleasanton, CA, USA) in the presence of probenecid (2.5 mM) and 0.05% Pluronic^®^ F-127 and incubated for 30 min at room temperature in the dark.

Cells were harvested after incubation, resuspended in 50:1 HBSS-HEPES buffer, and seeded in a black 96-well plate at 100,000 cells/well. To screen for antagonism, the compounds were added at a 5× concentration (final concentration of DMSO was 0.5%) and incubated for 15 min at 37 °C in a black Greiner 96-well flat-bottom plate before stimulation with 200 nM 7α,25-dihydroxycholesterol. Thereafter, plates were placed in a FlexStation 3 Multimode Plate Reader (Molecular Devices, Sunnyvale, CA, USA) for measurement. The instrument added the agonist (200 nM) prepared at 5× into the wells, and fluorescence signals were recorded for 120 s (via SoftMax Pro 7.1 Software, Molecular Devices, Sunnyvale, CA, USA). The calcium mobilization values were obtained ratiometrically using λ_ex_ 350 nm and λ_em_ 405/490 nm, respectively. Concentration-response curves were generated using GraphPad Prism 8 software (San Diego, CA, USA). IC_50_ values were determined by nonlinear regression analysis and are reported as mean ± standard deviation from 3 independent experiments. Each compound was tested in triplicate wells in three independent experiments performed on separate days (n = 3 independent experiments).

### 3.6. Statistical Analysis

Dose–response curves and IC_50_ values were generated using GraphPad Prism 8 (GraphPad Software, San Diego, CA, USA). Data are expressed as mean ± standard deviation from at least three independent experiments unless otherwise noted. Statistical significance was defined as *p* < 0.05. Raw data from each experiment were subtracted from the respective average vehicle control value and normalized (Y_max_ = the highest average among the dataset, Y_min_ = 0). The resulting data were fit with a non-linear regression curve (log[inhibitor] vs. normalized response), which was then graphed to give the respective dose–response curves (Figure 7). The average positive control (OHC) and average vehicle values were calculated in Microsoft Excel before entry into GraphPad Prism. All Hill Slope, pIC_50_, pIC_50_ 95% confidence intervals (CI), and R^2^ values were also calculated using GraphPad Prism.

## Figures and Tables

**Table 1:** Structures of benzimidazole compounds and calcium mobilization IC_50_ values. IC_50_ and Hill slope values are presented as the mean ± standard deviation of 3 independent experiments. pIC_50_ and pIC_50_ 95% Confidence Interval (CI) are representative values chosen from each compound dataset.

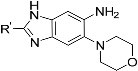						
Compound	Glide Score	R	IC_50_ (nM)	Hill Slope	pIC_50_	pIC_50_ 95% CI
**1**	−6.172	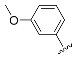	15.8 ± 6.8	−1.10 ± 0.27	7.805	7.997 to 7.607
**2**	−5.660	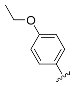	45.7 ± 8.2	−1.18 ± 0.17	7.330	7.480 to 7.188
**3**	−5.617	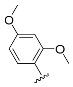	19.3 ± 8.5	−1.20 ± 0.24	7.428	7.583 to 7.271
**4**	−8.667	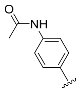	137.3 ± 17.5	−1.08 ± 0.22	6.650	6.774 to 6.521
**5**	−8.426	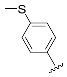	15.7 ± 2.5	−1.14 ± 0.05	7.475	7.614 to 7.337
**6**	−8.550	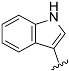	237.0 ± 11.4	−0.81 ± 0.27	6.840	6.973 to 6.707
**7**	−9.744	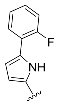	1211.0 ± 390.0	−0.52 ± 0.17	5.982	6.185 to 5.775
**8**	−10.251	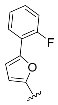	18.0 ± 2.2	−0.72 ± 0.09	7.685	7.965 to 7.406

## Data Availability

Dataset available on request from the authors.

## Supplementary Materials

The following supporting information can be downloaded at: https://www.mdpi.com/article/10.3390/molecules31193469/s1.
