# Supplementary material for: Development of G Protein-Coupled Receptor 183 Benzimidazole Antagonists Utilizing a Core-Hopping Approach
Source: Molecules. 2026 Sep 29;31(19):3469. doi: 10.3390/molecules31193469 (PMC13666532; doi:10.3390/molecules31193469)
Supplement: Supplementary file 1 [file molecules-31-03469-s001.zip › molecules-4511178-supplementary.pdf]

Supplemental Information for:

Development of G Protein-Coupled Receptor 183 Benzimidazole Antagonists Utilizing a Core-Hopping Approach

Angelina Ellis [1], Israel Olayide [1], Vanja Kalajdzic [1], Daniela Salvemini [1,2,3,4] and Christopher Arnatt [1,2,3,4,*]

1 Department of Chemistry, Saint Louis University, St. Louis, MO 63103, USA

2 Department of Pharmacology and Physiology, Saint Louis University, St. Louis, MO 63103, USA

3 Institute for Drug and Biotherapeutic Innovation, Saint Louis University, St. Louis, MO 63103, USA

4 Institute for Translational Neuroscience, Saint Louis University, St. Louis, MO 63103, USA

* Correspondence: chris.arnatt@slu.edu

Scaffold	Glide score	Prime MM-GBSA Score (kcal/mol)
**1**	-12.303	-77.15
**2**	-11.949	-63.47
**3**	-11.530	-62.98
**4**	-11.271	-65.83
**5**	-11.453	-62.51
**6**	-11.231	-72.29
**7**	-11.221	-67.90
**8**	-11.285	-72.38
**9**	-11.191	-68.40
**10**	-11.264	-70.82

Table S1: Glide and Prime MM-GBSA scores for the top ten scaffolds produced by the core-hopping virtual screening workflow. Final hit scaffolds were post-processed through Prime MM-GBSA. Structures for each scaffold can be seen in the main text (Figure 4).

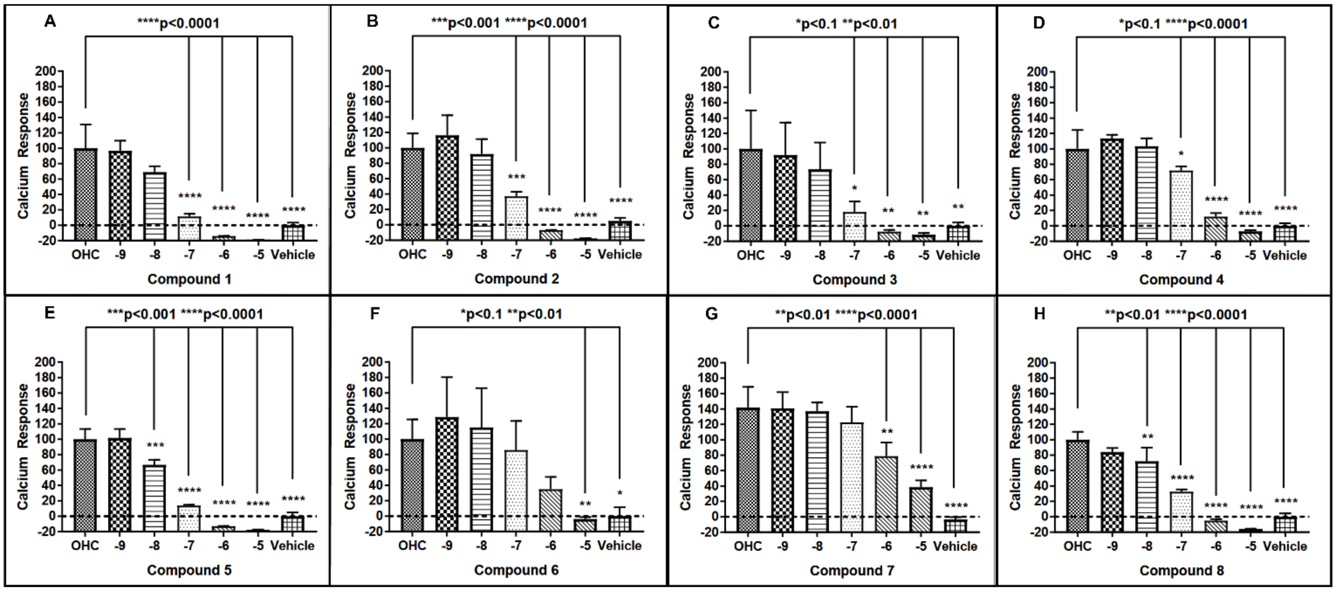

Figure S1: Dunnett's post hoc multiple comparison ANOVA versus 7α,25-dihydroxycholesterol (OHC) of compounds 1-8 (A-H) where α = 0.05, *p < 0.1, **p < 0.01, ***p < 0.001, ****p < 0.0001. Datasets are represented as a normalized average, n = 3.

Int 1

**Data file:**	ae-2-150-org.dx		
**Sequence Name:**	SingleSample	**Project Name:**	MeyersLab
**Sample name:**	ae-2-150-org	**Operator:**	SYSTEM (SYSTEM)
**Instrument:**	LCMS	**Injection date:**	2026-04-15 13:11:26-05:00
**Inj. volume:**	3.000 µL	**Location:**	95
**Acq. method:**	Meyerslab-Sept-24.amx	**Type:**	Sample
**Processing method:**	*Method Test-1.pmx	**Sample amount:**	0.00
**Manually modified:**	Manual Integration		

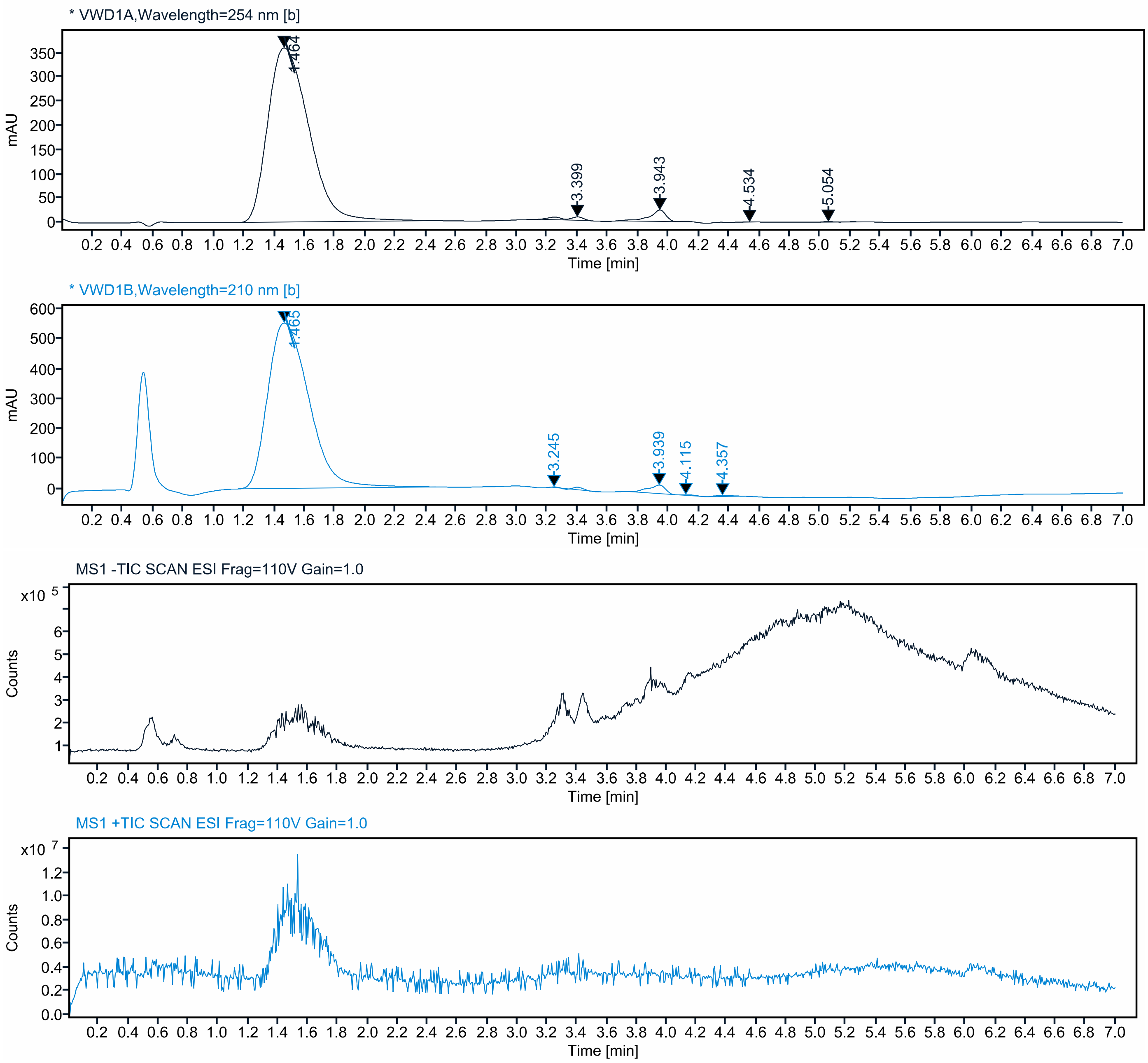

Signal: * VWD1A,Wavelength=254 nm [b]

RT [min]	Type	Width [min]	Area	Height	Area%
1.464	MM m	1.30	6774.38	360.22	96.28
3.399	MM m	0.35	74.10	7.13	1.05
3.943	MM m	0.50	180.81	23.56	2.57
4.534	MM m	0.11	0.69	0.18	0.01
5.054	MM m	0.30	5.85	0.93	0.08
		**Sum**	**7035.83**		

Signal: * VWD1B,Wavelength=210 nm [b]

RT [min]	Type	Width [min]	Area	Height	Area%
1.465	MM m	1.30	10419.60	553.84	97.18
3.245	MM m	0.31	35.18	1.47	0.33
3.939	MM m	0.31	223.37	28.74	2.08
4.115	MM m	0.21	13.86	2.19	0.13
4.357	MM m	0.25	29.99	3.74	0.28
		**Sum**	**10722.00**		

Signal: MS1 +TIC SCAN ESI Frag=110V Gain=1.0

RT [min]	Type	Width [min]	Area	Height	Area%
1.529	MM m	1.09	99232292.16	10328407.36	90.45
3.412	MM m	0.38	10481517.37	1906575.09	9.55
		**Sum**	**109713809.53**		

Signal: MS1 -TIC SCAN ESI Frag=110V Gain=1.0

RT [min]	Type	Width [min]	Area	Height	Area%
1.536	MM m	0.72	2847308.07	193029.42	55.17
3.307	MM m	0.41	1458451.70	173623.07	28.26
3.893	MM m	0.41	855095.26	149868.54	16.57
		**Sum**	**5160855.02**		

Int 2a

**Data file:**	AE-2-160-10.dx		
**Sequence Name:**	SingleSample	**Project Name:**	MeyersLab
**Sample name:**	AE-2-160-10	**Operator:**	SYSTEM (SYSTEM)
**Instrument:**	LCMS	**Injection date:**	2026-05-12 15:35:24-05:00
**Inj. volume:**	3.000 µL	**Location:**	91
**Acq. method:**	Meyerslab-Sept-24.amx	**Type:**	Sample
**Processing method:**	*Method Test-1.pmx	**Sample amount:**	0.00
**Manually modified:**	Manual Integration		

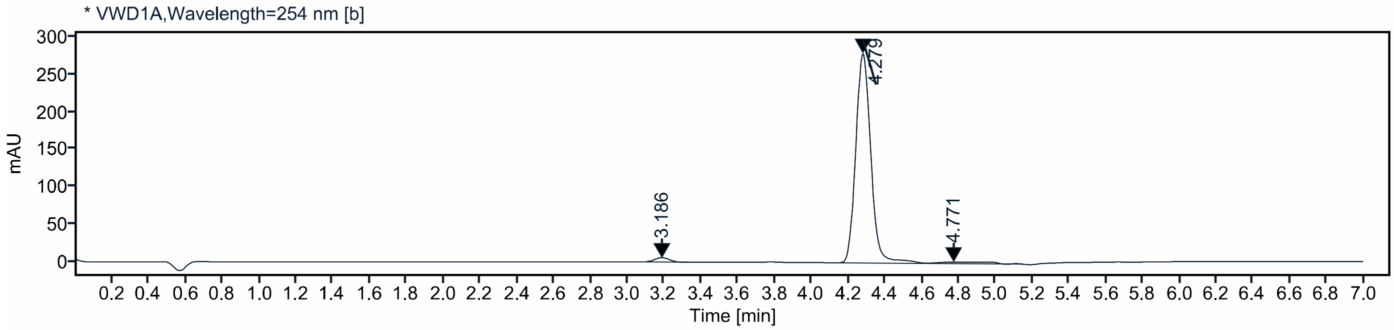

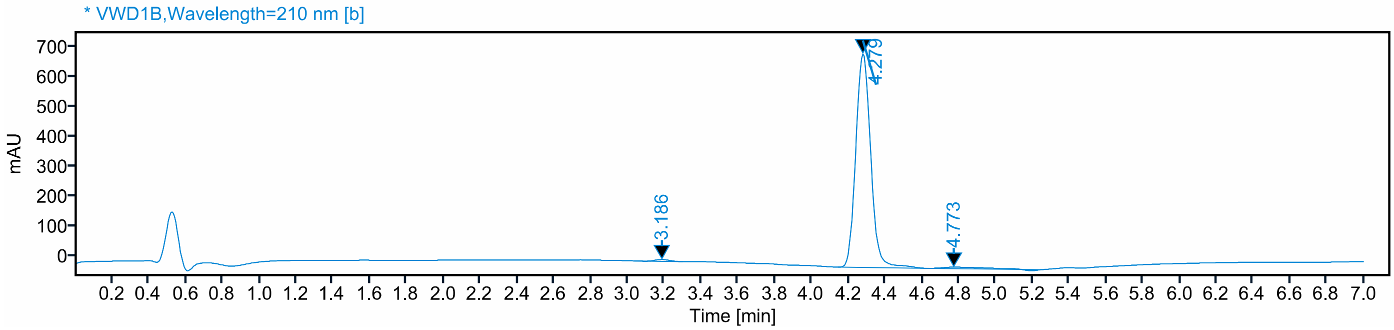

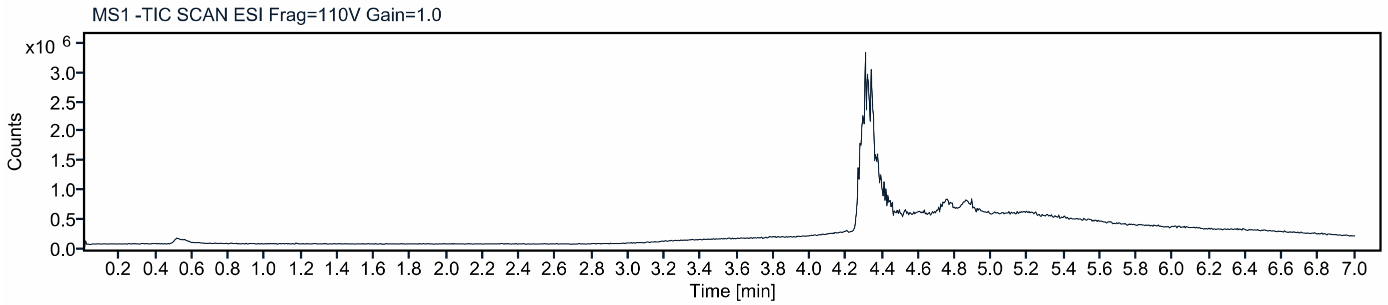

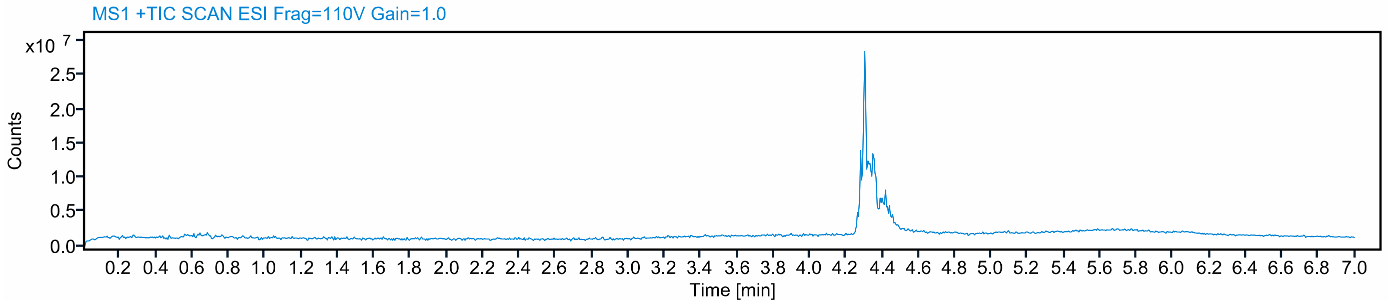

Signal: * VWD1A,Wavelength=254 nm [b]

RT [min]	Type	Width [min]	Area	Height	Area%
3.186	BB	0.27	33.31	5.95	1.98
4.279	BM m	0.50	1603.96	281.29	95.41
4.771	MM m	0.52	43.80	2.15	2.61
		**Sum**	**1681.08**		

Signal: * VWD1B,Wavelength=210 nm [b]

RT [min]	Type	Width [min]	Area	Height	Area%
3.186	MM m	0.26	28.93	5.92	0.70
4.279	BB	0.49	4023.36	713.01	97.24
4.773	MM m	0.61	85.35	4.98	2.06
		**Sum**	**4137.64**		

Signal: MS1 +TIC SCAN ESI Frag=110V Gain=1.0

RT [min]	Type	Width [min]	Area	Height	Area%
4.302	MM m	0.42	94885481.90	26713356.82	100.00
		**Sum**	**94885481.90**		

Signal: MS1 -TIC SCAN ESI Frag=110V Gain=1.0

RT [min]	Type	Width [min]	Area	Height	Area%
4.304	MM m	0.32	14206370.98	2954763.26	88.73
4.661	MM n	0.18	232887.04	74135.55	1.45
4.891	MM m	0.27	1572199.08	223332.90	9.82
		**Sum**	**16011457.10**		

Int 2b

Data file: ae-2-191-14-15.dx

**Sequence Name:**	SingleSample	**Project Name:**	MeyersLab
**Sample name:**	ae-2-191-14-15	**Operator:**	SYSTEM (SYSTEM)
**Instrument:**	LCMS	**Injection date:**	2026-07-28 14:36:55-05:00
**Inj. volume:**	3.000 µL	**Location:**	91
**Acq. method:**	Meyerslab-Sept-24.amx	**Type:**	Sample
**Processing method:**	Method Test-1.pmx	**Sample amount:**	0.00
**Manually modified:**	Manual Integration		

* VWD1A,Wavelength=254 nm [b]

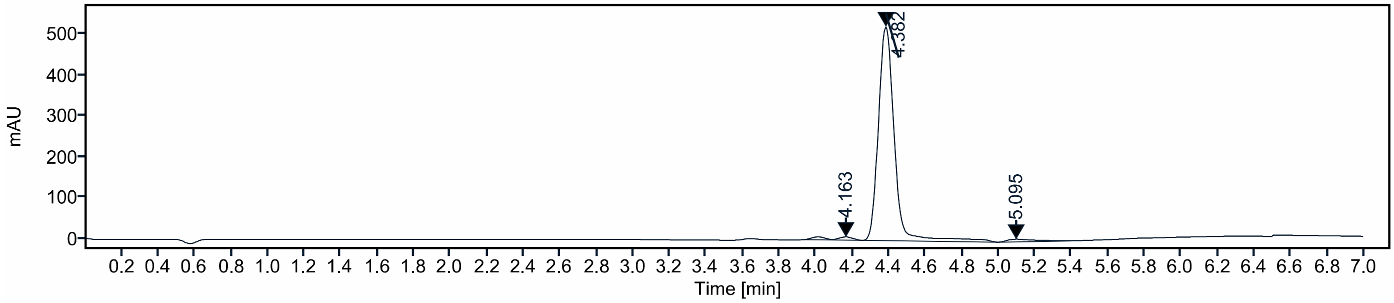

* VWD1B,Wavelength=210 nm [b]

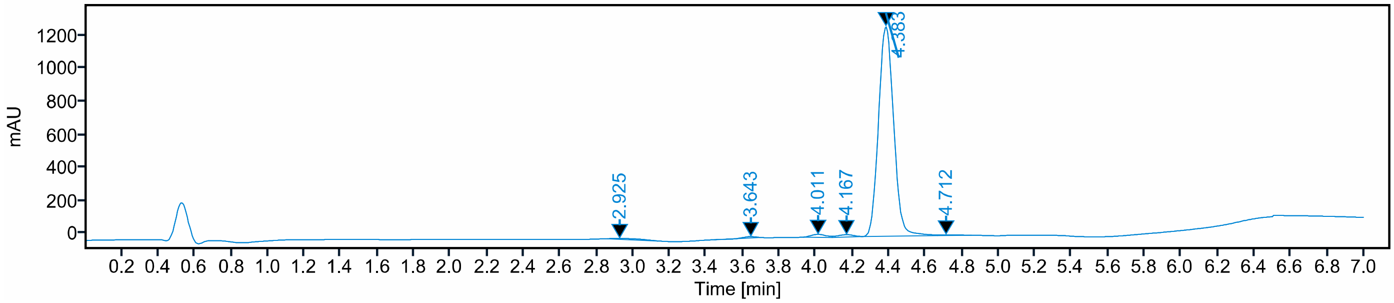

MS1 -TIC SCAN ESI Frag=110V Gain=1.0

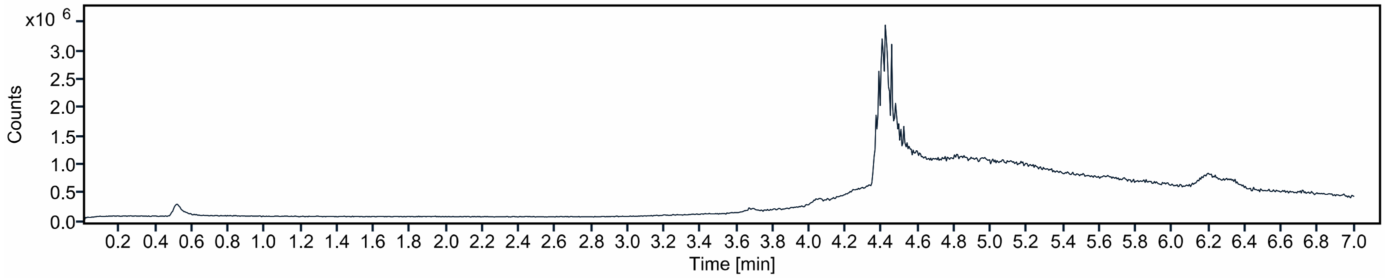

MS1 +TIC SCAN ESI Frag=110V Gain=1.0

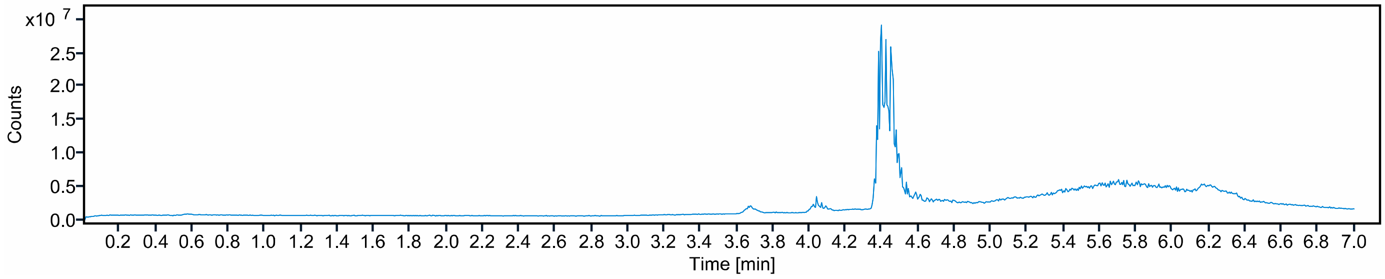

Signal: * VWD1A,Wavelength=254 nm [b]

RT [min]	Type	Width [min]	Area	Height	Area%
4.163	MB m	0.33	82.15	8.14	2.45
4.382	BB	0.74	3191.52	523.38	95.28
5.095	BV	0.39	76.03	6.60	2.27
		**Sum**	**3349.70**		

Signal: * VWD1B,Wavelength=210 nm [b]

RT [min]	Type	Width [min]	Area	Height	Area%
2.925	MM m	0.27	63.42	4.35	0.83
3.643	MM m	0.25	27.83	7.75	0.36
4.011	MM m	0.16	95.79	17.13	1.25
4.167	VB	0.16	81.75	14.05	1.07
4.383	BM m	0.41	7342.92	1269.78	96.08
4.712	MM m	0.19	30.92	4.18	0.40
		**Sum**	**7642.63**		

Signal: MS1 +TIC SCAN ESI Frag=110V Gain=1.0

RT [min]	Type	Width [min]	Area	Height	Area%
3.673	MM m	0.18	3903053.17	1100254.39	2.49
4.039	MM m	0.23	5602086.97	2266580.73	3.57
4.395	MM m	0.49	147218440.54	27564676.27	93.94
		**Sum**	**156723580.68**		

Signal: MS1 -TIC SCAN ESI Frag=110V Gain=1.0

RT [min]	Type	Width [min]	Area	Height	Area%
4.416	MM m	0.47	16854253.87	2733232.69	100.00
		**Sum**	**16854253.87**		

Int 2c

**Data file:**	ae-2-169-16.dx		
**Sequence Name:**	SingleSample	**Project Name:**	MeyersLab
**Sample name:**	ae-2-169-16	**Operator:**	SYSTEM (SYSTEM)
**Instrument:**	LCMS	**Injection date:**	2026-05-15 16:55:38-05:00
**Inj. volume:**	3.000 µL	**Location:**	94
**Acq. method:**	Meyerslab-Sept-24.amx	**Type:**	Sample
**Processing method:**	*Method Test-1.pmx	**Sample amount:**	0.00
**Manually modified:**	Manual Integration		

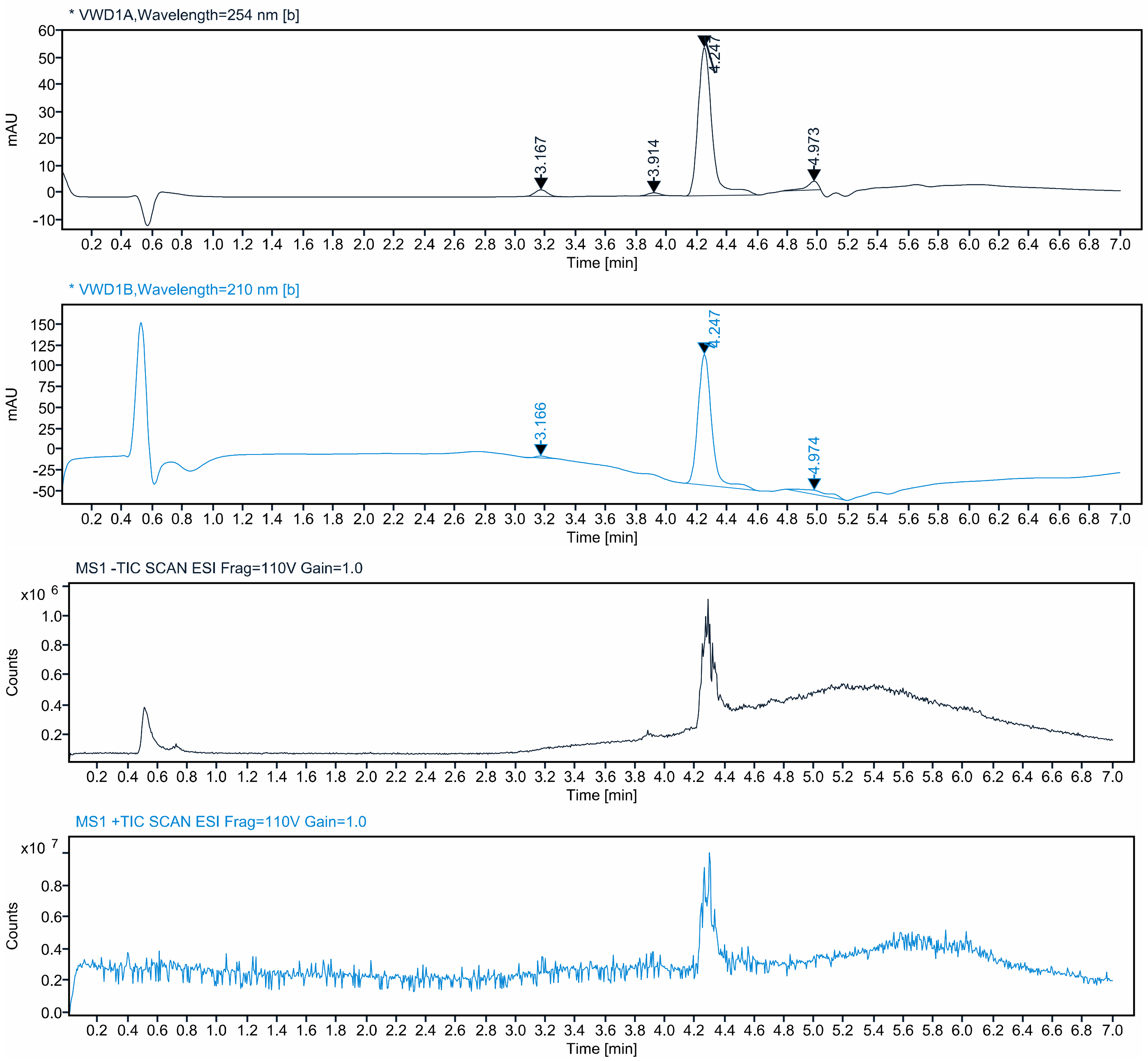

Signal: * VWD1A,Wavelength=254 nm [b]

RT [min]	Type	Width [min]	Area	Height	Area%
3.167	MB m	0.27	13.77	2.44	3.52
3.914	MM m	0.17	5.39	1.04	1.38
4.247	BM m	0.49	355.56	55.01	90.79
4.973	MM m	0.25	16.89	3.24	4.31
		**Sum**	**391.61**		

Signal: * VWD1B,Wavelength=210 nm [b]

RT [min]	Type	Width [min]	Area	Height	Area%
3.166	MM m	0.23	11.16	2.34	1.03
4.247	BM m	0.48	995.83	157.45	92.12
4.974	MM m	0.41	74.03	5.05	6.85
		**Sum**	**1081.01**		

Signal: MS1 +TIC SCAN ESI Frag=110V Gain=1.0

RT [min]	Type	Width [min]	Area	Height	Area%
4.294	MM m	0.50	34076827.14	7263205.01	100.00
		**Sum**	**34076827.14**		

Signal: MS1 -TIC SCAN ESI Frag=110V Gain=1.0

RT [min]	Type	Width [min]	Area	Height	Area%
3.885	MM m	0.19	153182.72	58536.07	3.87
4.287	MM m	0.35	3806274.41	832143.60	96.13
		**Sum**	**3959457.13**		

Int 2d

**Data file:**	ae-2-186-25-26.dx		
**Sequence Name:**	SingleSample	**Project Name:**	MeyersLab
**Sample name:**	ae-2-186-25-26	**Operator:**	SYSTEM (SYSTEM)
**Instrument:**	LCMS	**Injection date:**	2026-07-28 14:45:40-05:00
**Inj. volume:**	3.000 µL	**Location:**	92
**Acq. method:**	Meyerslab-Sept-24.amx	**Type:**	Sample
**Processing method:**	*Method Test-1.pmx	**Sample amount:**	0.00
**Manually modified:**	Manual Integration		

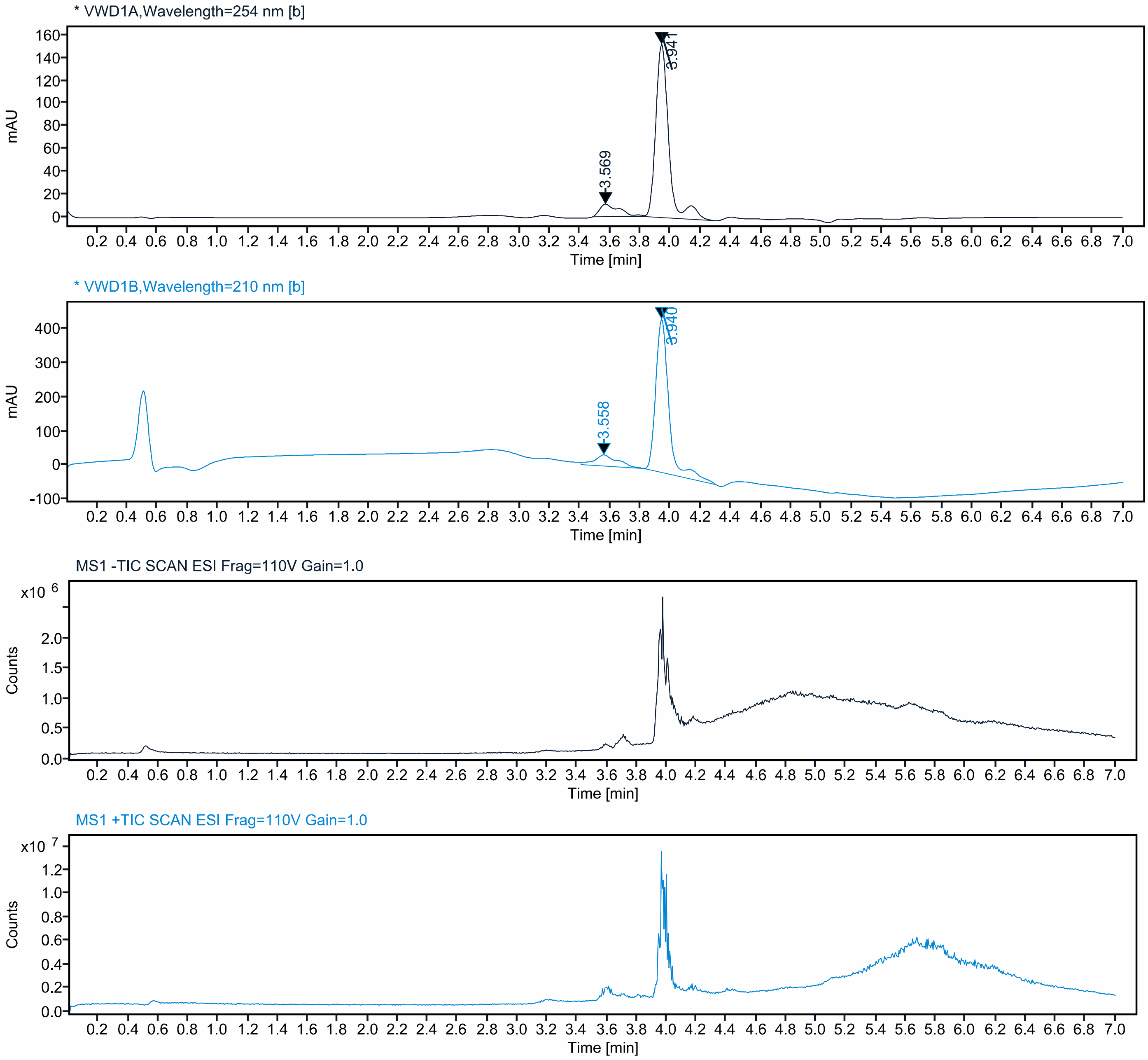

Signal: * VWD1A,Wavelength=254 nm [b]

RT [min]	Type	Width [min]	Area	Height	Area%
3.569	MM m	0.33	98.94	10.92	9.75
3.941	MM m	0.48	916.08	152.61	90.25
		**Sum**	**1015.01**		

Signal: * VWD1B,Wavelength=210 nm [b]

RT [min]	Type	Width [min]	Area	Height	Area%
3.558	MM m	0.42	357.98	32.93	11.59
3.940	MM m	0.48	2729.65	450.71	88.41
		**Sum**	**3087.62**		

Signal: MS1 +TIC SCAN ESI Frag=110V Gain=1.0

RT [min]	Type	Width [min]	Area	Height	Area%
3.611	MM m	0.37	7374199.76	1130080.63	17.20
3.964	MM m	0.26	35287010.00	12185464.67	82.28
4.295	MM n	0.18	222950.12	146473.72	0.52
		**Sum**	**42884159.88**		

Signal: MS1 -TIC SCAN ESI Frag=110V Gain=1.0

RT [min]	Type	Width [min]	Area	Height	Area%
3.710	MM m	0.32	774666.12	195189.54	8.68
3.973	MM m	0.33	7923311.18	2270724.99	88.74
4.178	MM m	0.13	231091.95	120320.98	2.59
		**Sum**	**8929069.26**		

Int 2e

**Data file:**	ae-2-85-15thru16.dx		
**Sequence Name:**	SingleSample	**Project Name:**	MeyersLab
**Sample name:**	ae-2-85-15thru16	**Operator:**	SYSTEM (SYSTEM)
**Instrument:**	LCMS	**Injection date:**	2025-10-31 14:49:02-05:00
**Inj. volume:**	3.000 µL	**Location:**	3
**Acq. method:**	Meyerslab-Sept-24.amx	**Type:**	Sample
**Processing method:**	*Method Test-1.pmx	**Sample amount:**	0.00
**Manually modified:**	Manual Integration		

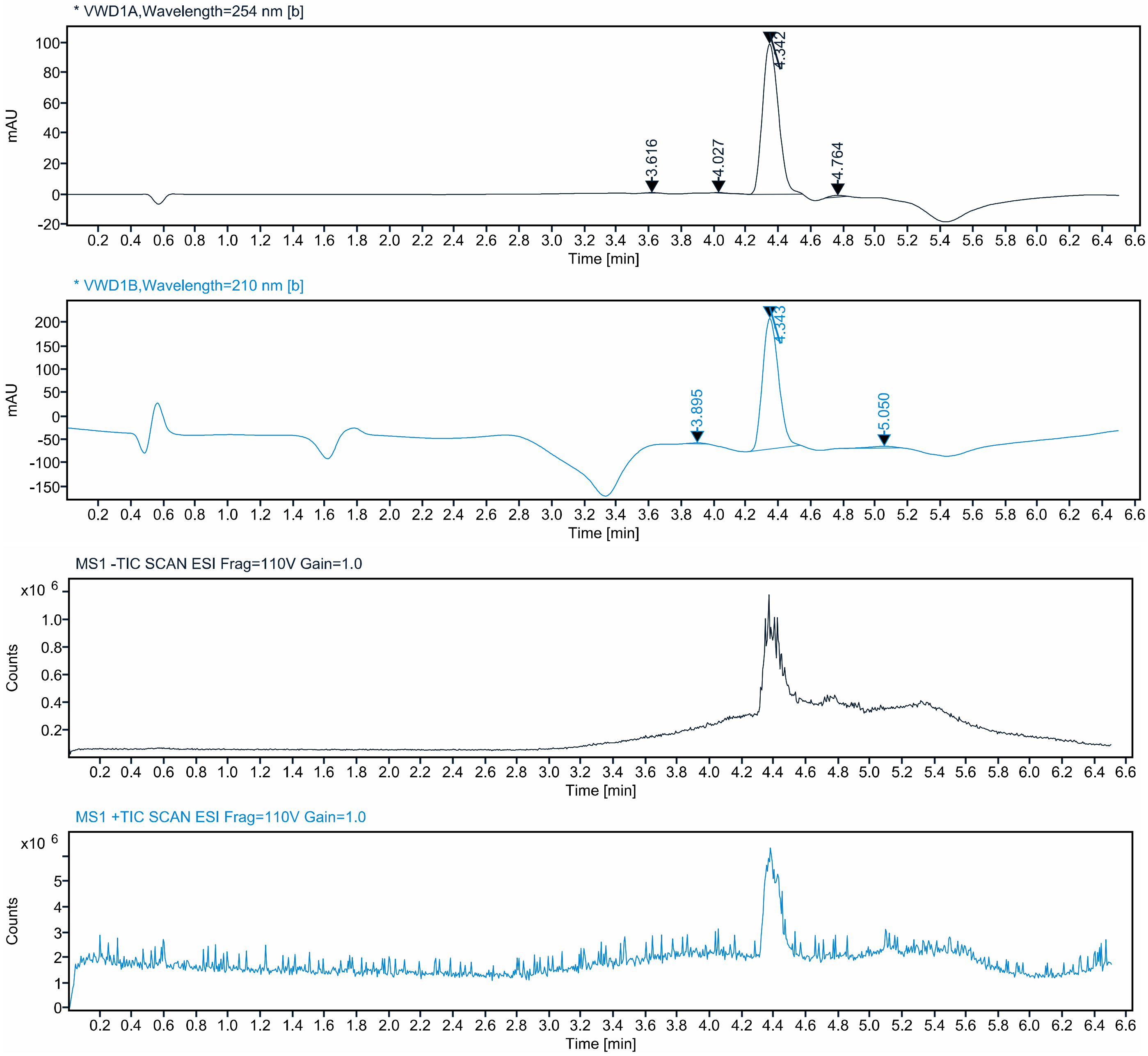

Signal: * VWD1A,Wavelength=254 nm [b]

RT [min]	Type	Width [min]	Area	Height	Area%
3.616	MM m	0.10	1.02	0.27	0.15
4.027	MM m	0.22	1.29	0.30	0.19
4.342	MM m	0.33	677.64	99.64	98.69
4.764	MM m	0.14	6.66	1.13	0.97
		**Sum**	**686.61**		

Signal: * VWD1B,Wavelength=210 nm [b]

RT [min]	Type	Width [min]	Area	Height	Area%
3.895	MM m	0.14	10.58	1.91	0.54
4.343	MM m	0.32	1905.59	279.47	97.53
5.050	MM m	0.35	37.75	3.61	1.93
		**Sum**	**1953.91**		

Signal: MS1 +TIC SCAN ESI Frag=110V Gain=1.0

RT [min]	Type	Width [min]	Area	Height	Area%
4.375	MM m	0.25	25650705.14	4205150.97	100.00
		**Sum**	**25650705.14**		

Signal: MS1 -TIC SCAN ESI Frag=110V Gain=1.0

RT [min]	Type	Width [min]	Area	Height	Area%
4.367	MM m	0.27	4419227.67	829484.16	89.69
4.732	MM m	0.27	508019.35	66408.72	10.31
		**Sum**	**4927247.02**		

Int 2f

**Data file:**	ae-2-87-14.dx		
**Sequence Name:**	SingleSample	**Project Name:**	MeyersLab
**Sample name:**	ae-2-87-14	**Operator:**	SYSTEM (SYSTEM)
**Instrument:**	LCMS	**Injection date:**	2025-11-17 15:26:45-06:00
**Inj. volume:**	3.000 µL	**Location:**	93
**Acq. method:**	Meyerslab-Sept-24.amx	**Type:**	Sample
**Processing method:**	*Method Test-1.pmx	**Sample amount:**	0.00
**Manually modified:**	Manual Integration		

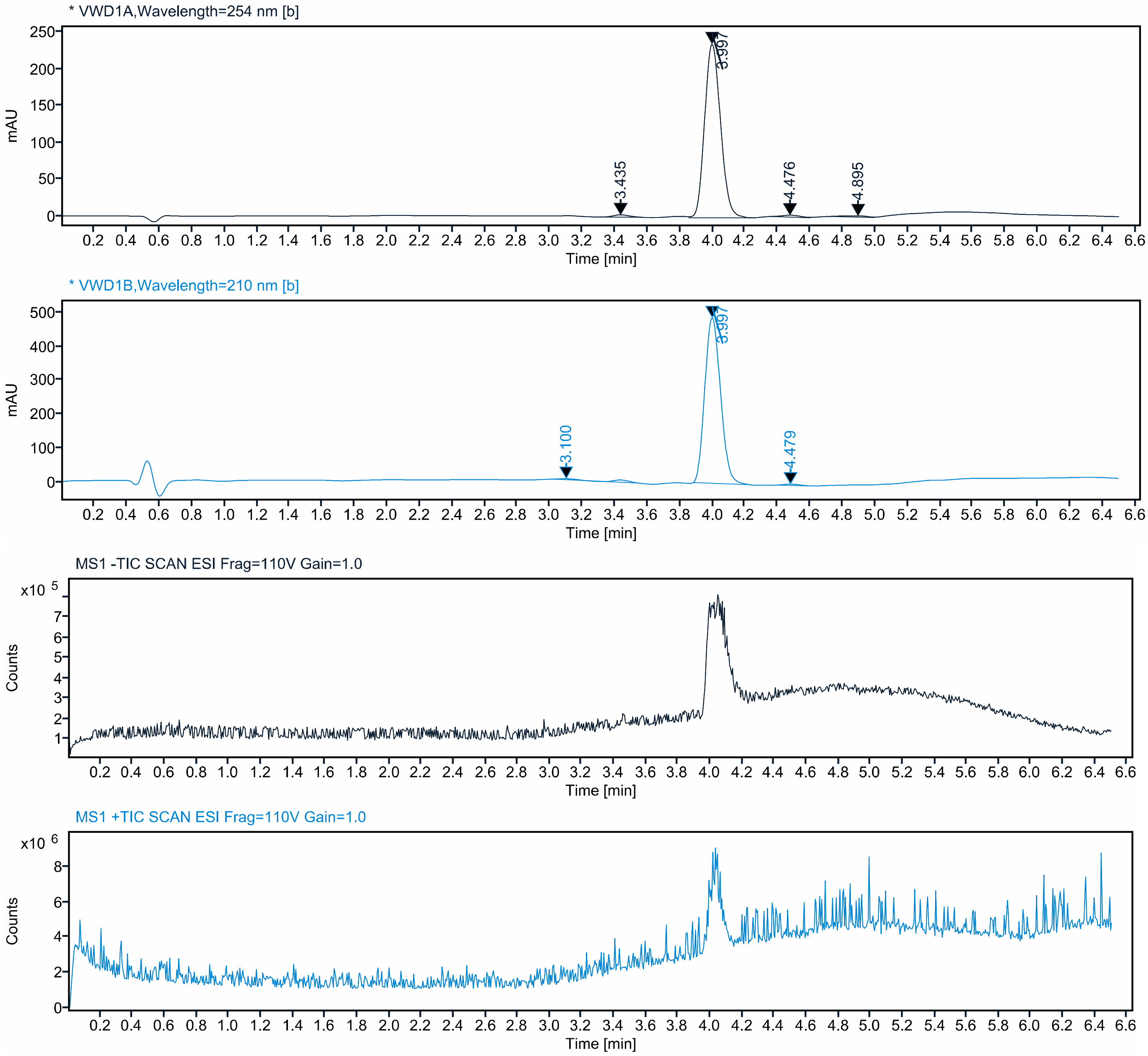

Signal: * VWD1A,Wavelength=254 nm [b]

RT [min]	Type	Width [min]	Area	Height	Area%
3.435	MM m	0.25	21.82	3.18	1.31
3.997	VB	0.39	1599.80	236.38	96.40
4.476	MM m	0.24	20.34	2.62	1.23
4.895	MV m	0.27	17.54	1.70	1.06
		**Sum**	**1659.49**		

Signal: * VWD1B,Wavelength=210 nm [b]

RT [min]	Type	Width [min]	Area	Height	Area%
3.100	MM m	0.52	58.51	2.86	1.75
3.997	MM m	0.34	3268.21	488.57	97.55
4.479	MM m	0.21	23.43	3.65	0.70
		**Sum**	**3350.15**		

Signal: MS1 +TIC SCAN ESI Frag=110V Gain=1.0

RT [min]	Type	Width [min]	Area	Height	Area%
4.033	MM m	0.22	26020014.04	5717147.56	100.00
		**Sum**	**26020014.04**		

Signal: MS1 -TIC SCAN ESI Frag=110V Gain=1.0

RT [min]	Type	Width [min]	Area	Height	Area%
4.049	MM m	0.31	4139552.33	546866.85	100.00
		**Sum**	**4139552.33**		

Int 2g

**Data file:**	AE-2-83-12.dx		
**Sequence Name:**	SingleSample	**Project Name:**	MeyersLab
**Sample name:**	AE-2-83-12	**Operator:**	SYSTEM (SYSTEM)
**Instrument:**	LCMS	**Injection date:**	2025-10-29 14:32:07-05:00
**Inj. volume:**	3.000 µL	**Location:**	1
**Acq. method:**	Meyerslab-Sept-24.amx	**Type:**	Sample
**Processing method:**	*Method Test-1.pmx	**Sample amount:**	0.00
**Manually modified:**	Manual Integration		

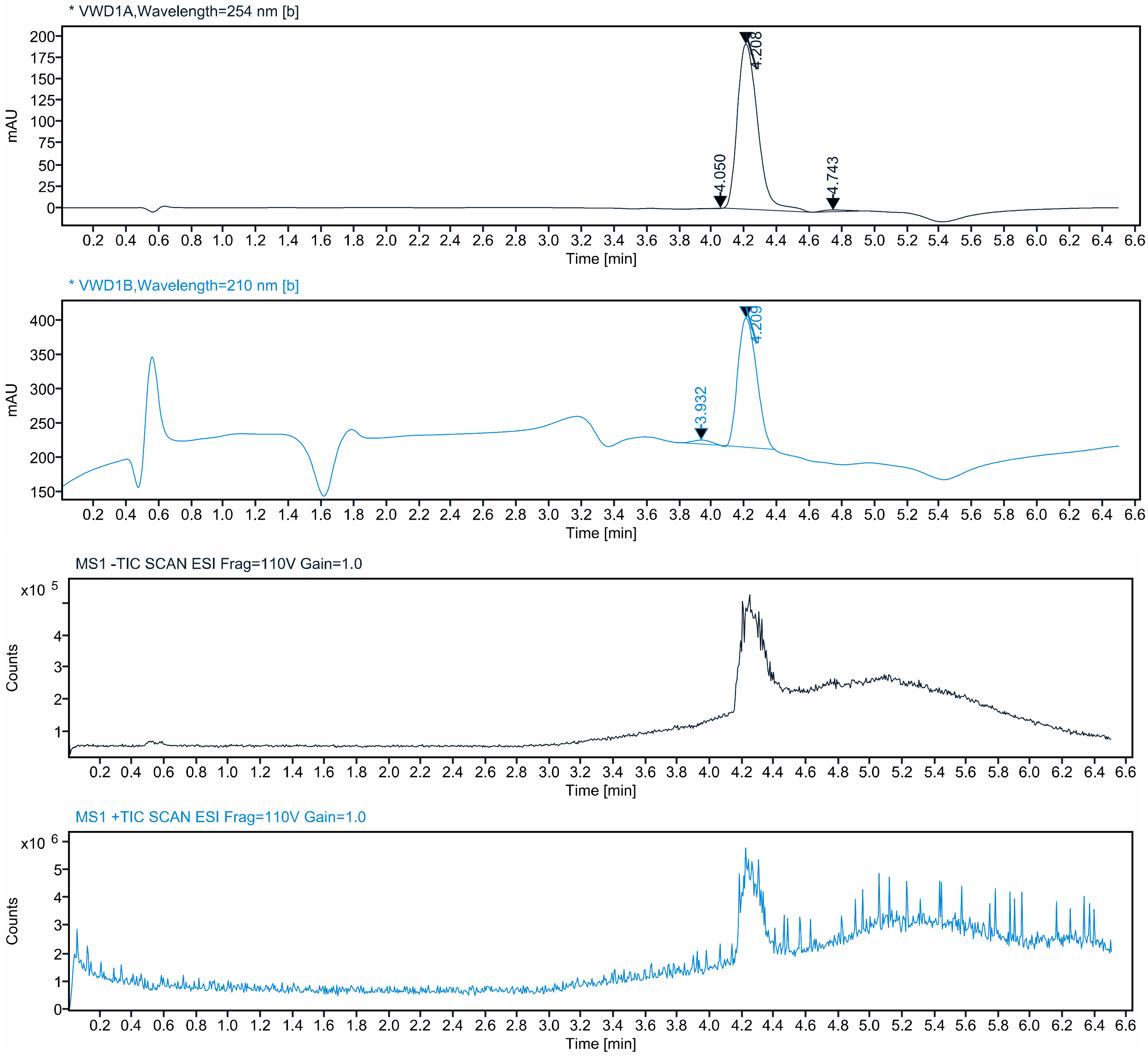

Signal: * VWD1A,Wavelength=254 nm [b]

RT [min]	Type	Width [min]	Area	Height	Area%
4.050	MM n	0.22	1.27	0.40	0.07
4.208	MM m	0.54	1689.22	193.66	98.74
4.743	MM m	0.31	20.27	1.98	1.18
		**Sum**	**1710.76**		

Signal: * VWD1B,Wavelength=210 nm [b]

RT [min]	Type	Width [min]	Area	Height	Area%
3.932	MM m	0.27	45.88	5.73	2.95
4.209	MM m	0.34	1508.94	188.13	97.05
		**Sum**	**1554.82**		

Signal: MS1 +TIC SCAN ESI Frag=110V Gain=1.0

RT [min]	Type	Width [min]	Area	Height	Area%
4.222	MM m	0.29	31227715.57	4155476.57	100.00
		**Sum**	**31227715.57**		

Signal: MS1 -TIC SCAN ESI Frag=110V Gain=1.0

RT [min]	Type	Width [min]	Area	Height	Area%
4.247	MM m	0.35	3114664.93	351847.40	100.00
		**Sum**	**3114664.93**		

Int 2h

**Data file:**	ae-2-88-18.dx		
**Sequence Name:**	SingleSample	**Project Name:**	MeyersLab
**Sample name:**	ae-2-88-18	**Operator:**	SYSTEM (SYSTEM)
**Instrument:**	LCMS	**Injection date:**	2025-11-17 15:35:00-06:00
**Inj. volume:**	3.000 µL	**Location:**	94
**Acq. method:**	Meyerslab-Sept-24.amx	**Type:**	Sample
**Processing method:**	*Method Test-1.pmx	**Sample amount:**	0.00
**Manually modified:**	Manual Integration		

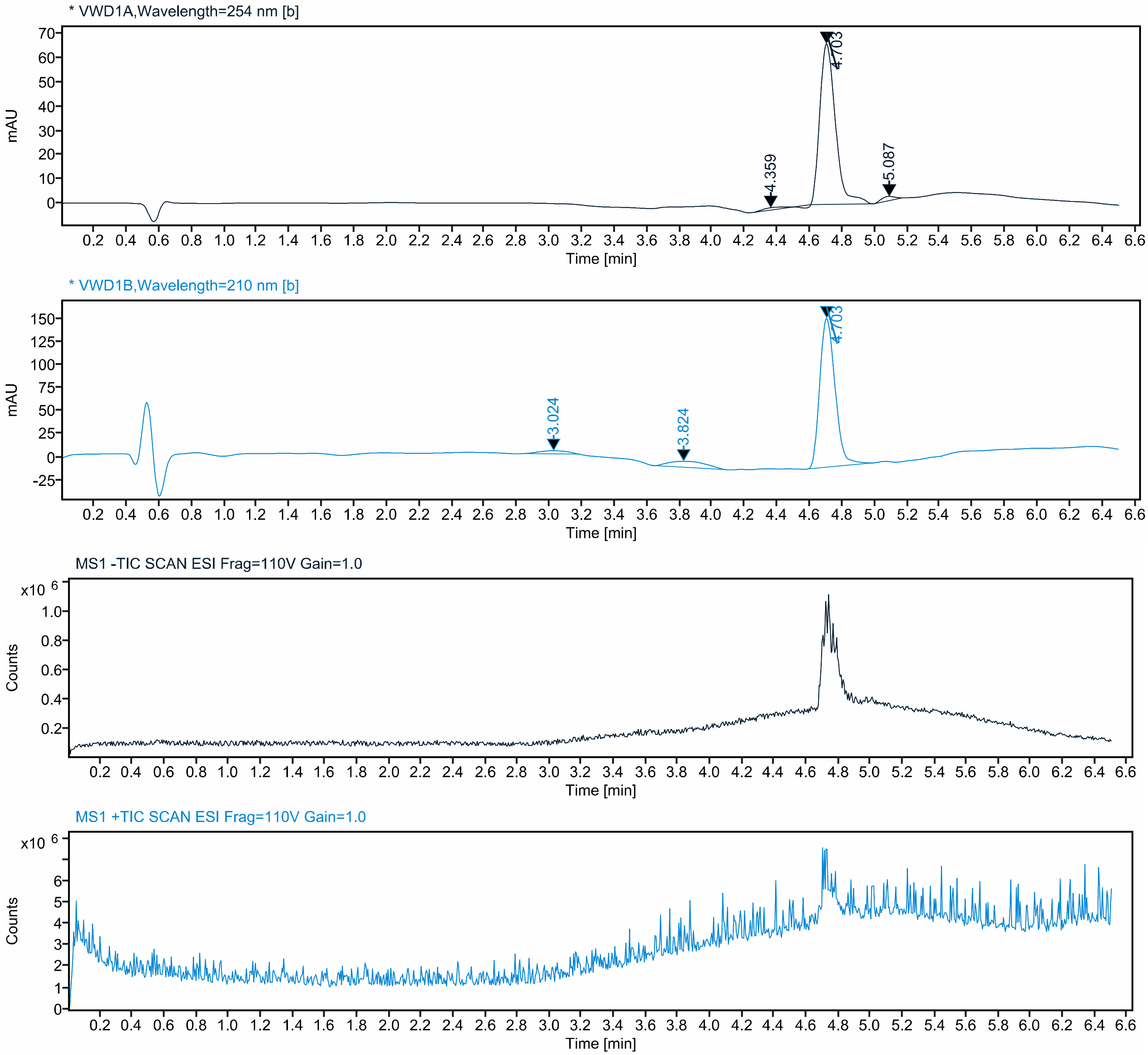

Signal: * VWD1A,Wavelength=254 nm [b]

RT [min]	Type	Width [min]	Area	Height	Area%
4.359	MM m	0.35	4.50	0.99	0.98
4.703	MM m	0.39	444.11	66.64	96.71
5.087	MM m	0.18	10.59	1.80	2.31
		**Sum**	**459.20**		

Signal: * VWD1B,Wavelength=210 nm [b]

RT [min]	Type	Width [min]	Area	Height	Area%
3.024	MM m	0.33	41.91	3.43	3.50
3.824	MM m	0.45	103.80	6.38	8.66
4.703	MM m	0.42	1052.22	161.88	87.84
		**Sum**	**1197.93**		

Signal: MS1 +TIC SCAN ESI Frag=110V Gain=1.0

RT [min]	Type	Width [min]	Area	Height	Area%
4.700	MM m	0.20	10705665.71	3194861.67	100.00
		**Sum**	**10705665.71**		

Signal: MS1 -TIC SCAN ESI Frag=110V Gain=1.0

RT [min]	Type	Width [min]	Area	Height	Area%
4.737	MM m	0.25	3728383.39	759080.61	100.00
		**Sum**	**3728383.39**		

Compound 1

Data file: ae-2-160-nh2-2nd-4-6.dx

**Sequence Name:**	SingleSample	**Project Name:**	MeyersLab
**Sample name:**	ae-2-160-nh2-2nd-4-6	**Operator:**	SYSTEM (SYSTEM)
**Instrument:**	LCMS	**Injection date:**	2026-05-21 15:37:16-05:00
**Inj. volume:**	3.000 µL	**Location:**	91
**Acq. method:**	Meyerslab-Sept-24.amx	**Type:**	Sample
**Processing method:**	*Method Test-1.pmx	**Sample amount:**	0.00
**Manually modified:**	Manual Integration		

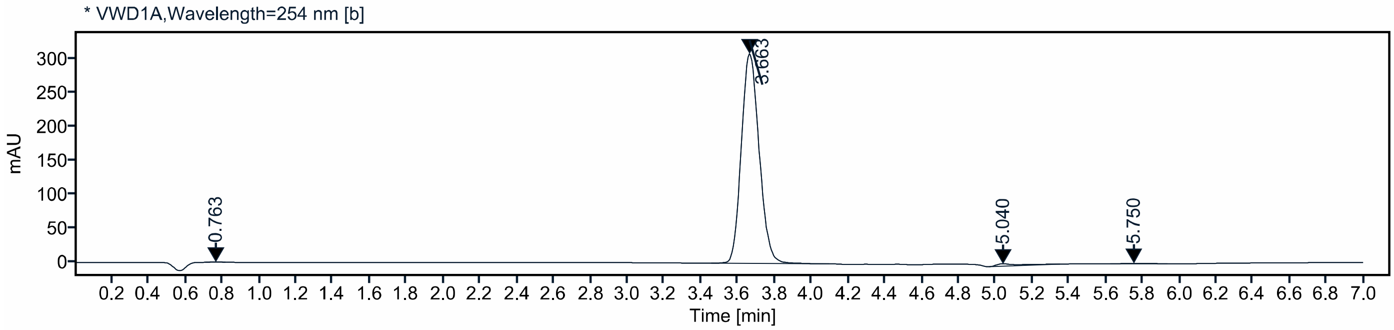

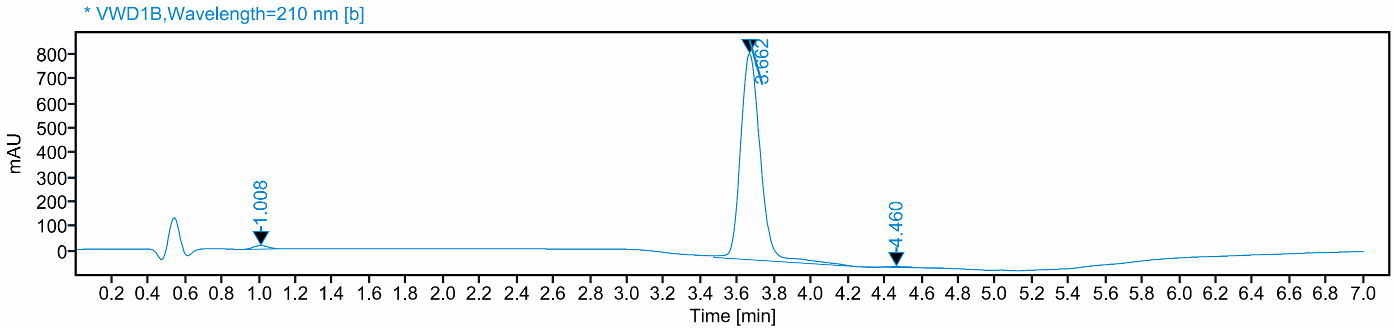

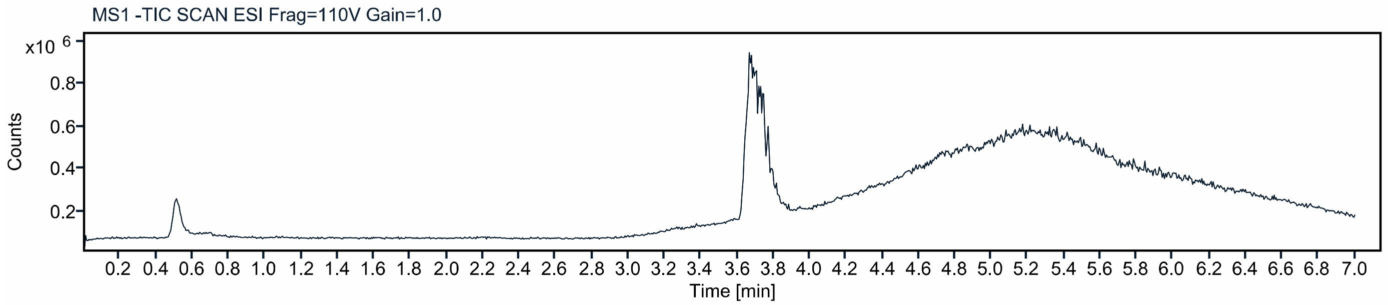

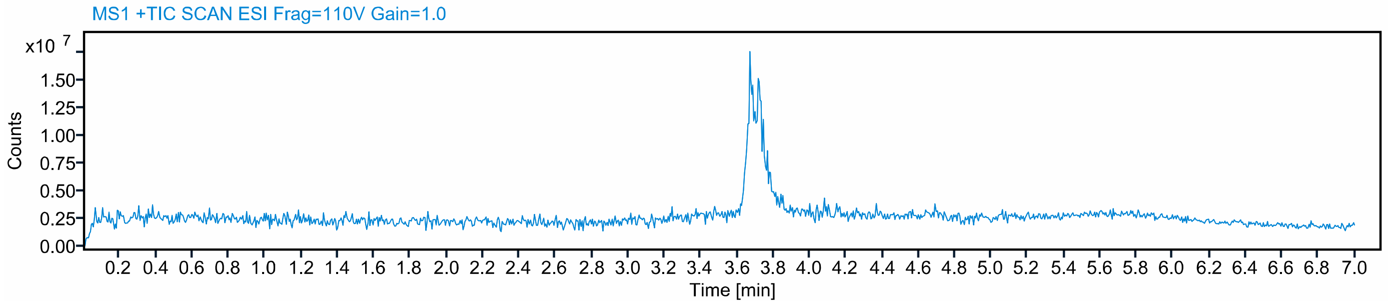

Signal: * VWD1A,Wavelength=254 nm [b]

RT [min]	Type	Width [min]	Area	Height	Area%
0.763	MM m	0.11	1.92	0.50	0.09
3.663	BM m	0.64	2079.19	308.55	98.17
5.040	MM m	0.38	29.63	3.55	1.40
5.750	MM m	0.31	7.18	0.72	0.34
		**Sum**	**2117.93**		

Signal: * VWD1B,Wavelength=210 nm [b]

RT [min]	Type	Width [min]	Area	Height	Area%
1.008	MM m	0.23	85.73	14.77	1.33
3.662	VB	0.79	6326.87	837.92	98.20
4.460	MM m	0.48	30.46	3.82	0.47
		**Sum**	**6443.07**		

Signal: MS1 +TIC SCAN ESI Frag=110V Gain=1.0

RT [min]	Type	Width [min]	Area	Height	Area%
3.669	MM m	0.45	77301407.20	14636648.96	100.00
		**Sum**	**77301407.20**		

Signal: MS1 -TIC SCAN ESI Frag=110V Gain=1.0

RT [min]	Type	Width [min]	Area	Height	Area%
3.666	MM m	0.44	5275539.91	774652.74	100.00
		**Sum**	**5275539.91**		

Compound 2

Data file: AE-2-172-NH2-3.dx

**Sequence Name:**	SingleSample	**Project Name:**	MeyersLab
**Sample name:**	AE-2-172-NH2-3	**Operator:**	SYSTEM (SYSTEM)
**Instrument:**	LCMS	**Injection date:**	2026-05-25 12:15:10-05:00
**Inj. volume:**	3.000 µL	**Location:**	91
**Acq. method:**	Meyerslab-Sept-24.amx	**Type:**	Sample
**Processing method:**	*Method Test-1.pmx	**Sample amount:**	0.00
**Manually modified:**	Manual Integration		

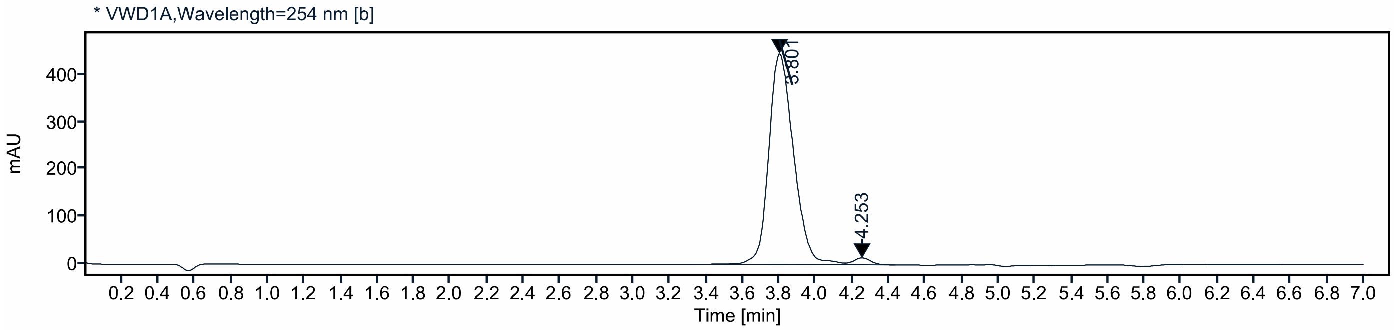

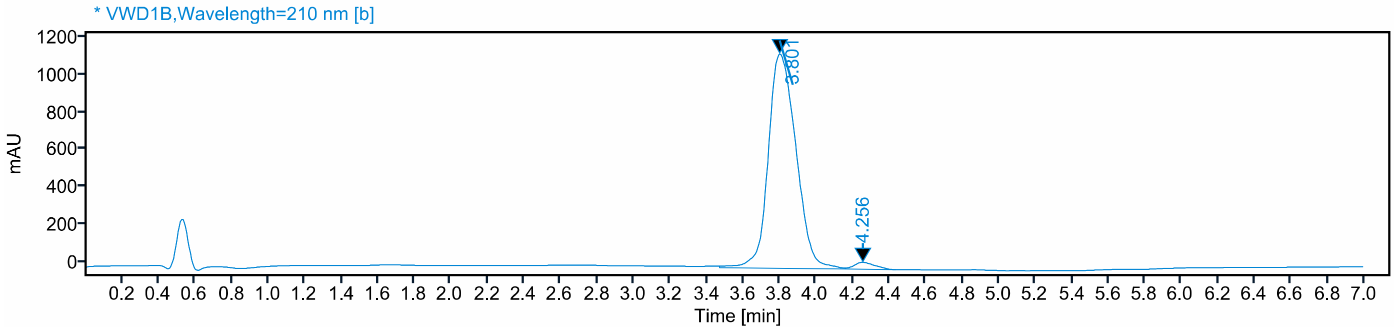

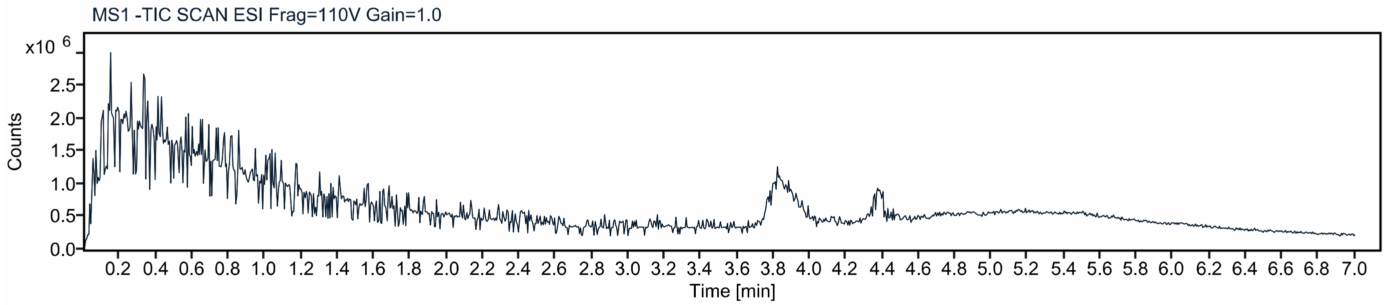

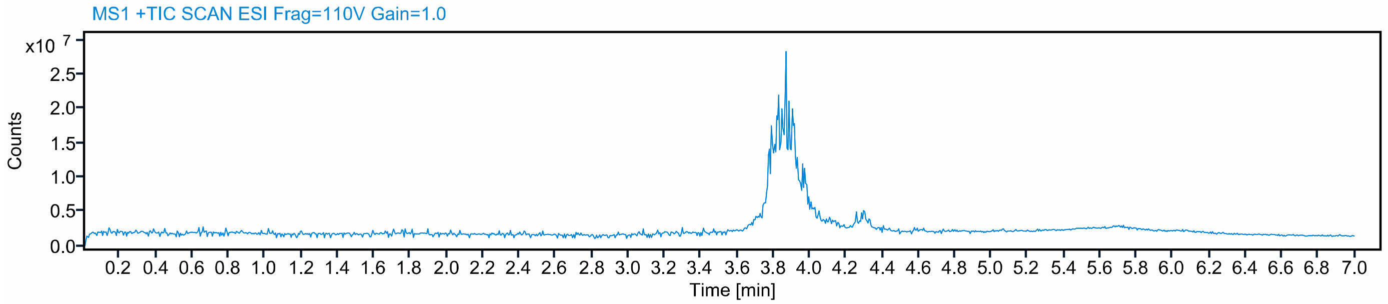

Signal: * VWD1A,Wavelength=254 nm [b]

RT [min]	Type	Width [min]	Area	Height	Area%
3.801	BV	0.84	4222.67	447.45	97.76
4.253	VB	0.27	96.54	14.63	2.24
		**Sum**	**4319.21**		

Signal: * VWD1B,Wavelength=210 nm [b]

RT [min]	Type	Width [min]	Area	Height	Area%
3.801	VV	0.69	12103.26	1142.68	97.67
4.256	VB	0.27	288.63	37.19	2.33
		**Sum**	**12391.89**		

Signal: MS1 +TIC SCAN ESI Frag=110V Gain=1.0

RT [min]	Type	Width [min]	Area	Height	Area%
3.866	MM m	0.57	191130765.46	25949725.25	96.86
4.296	MM m	0.23	6197886.71	2392204.47	3.14
		**Sum**	**197328652.17**		

Signal: MS1 -TIC SCAN ESI Frag=110V Gain=1.0

RT [min]	Type	Width [min]	Area	Height	Area%
3.820	MM m	0.45	6203995.11	853022.54	83.56
4.373	MM m	0.39	1220383.20	460841.87	16.44
		**Sum**	**7424378.30**		

Compound 3

**Data file:**	ae-2-169-nh2-2-.dx		
**Sequence Name:**	SingleSample	**Project Name:**	MeyersLab
**Sample name:**	ae-2-169-nh2-2-	**Operator:**	SYSTEM (SYSTEM)
**Instrument:**	LCMS	**Injection date:**	2026-05-26 11:13:50-05:00
**Inj. volume:**	3.000 µL	**Location:**	91
**Acq. method:**	Meyerslab-Sept-24.amx	**Type:**	Sample
**Processing method:**	*Method Test-1.pmx	**Sample amount:**	0.00
**Manually modified:**	Manual Integration		

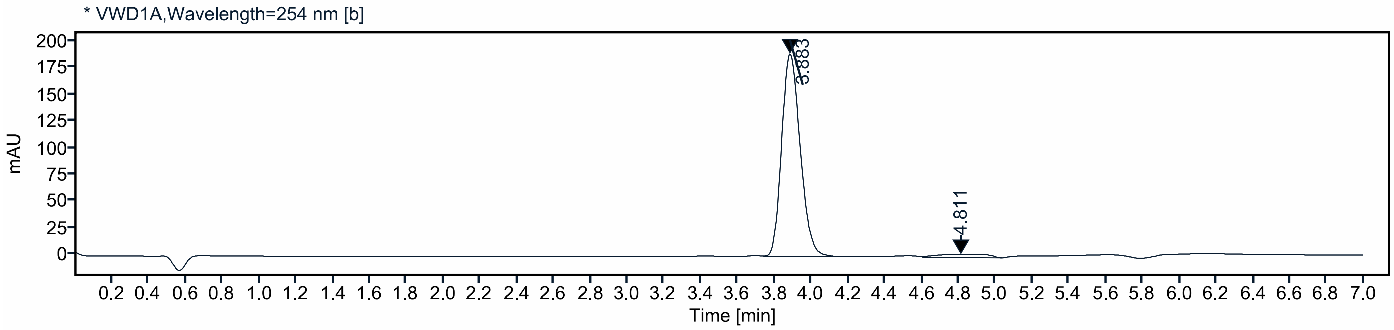

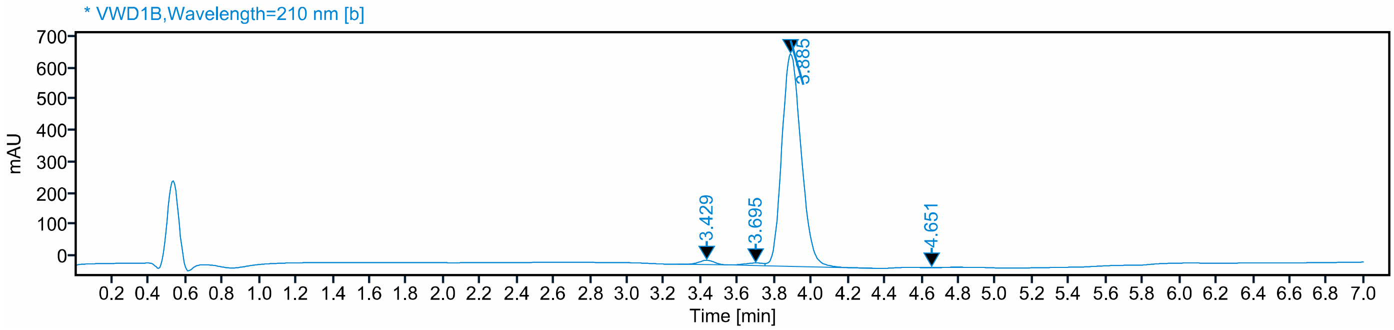

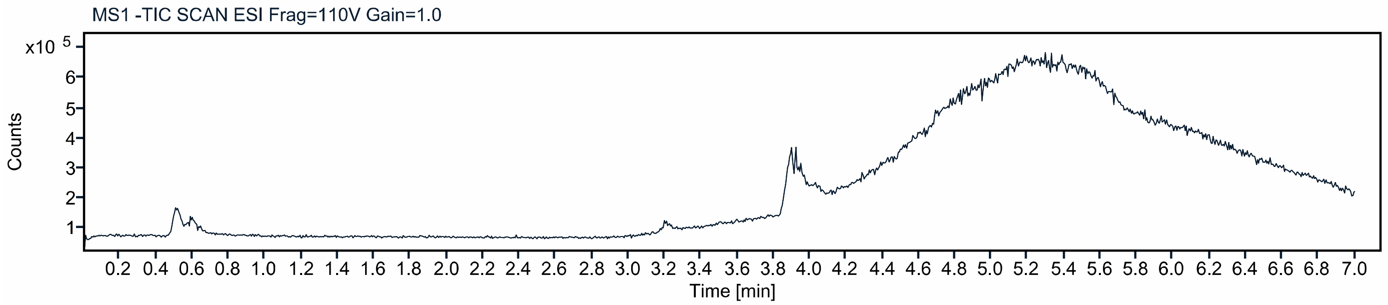

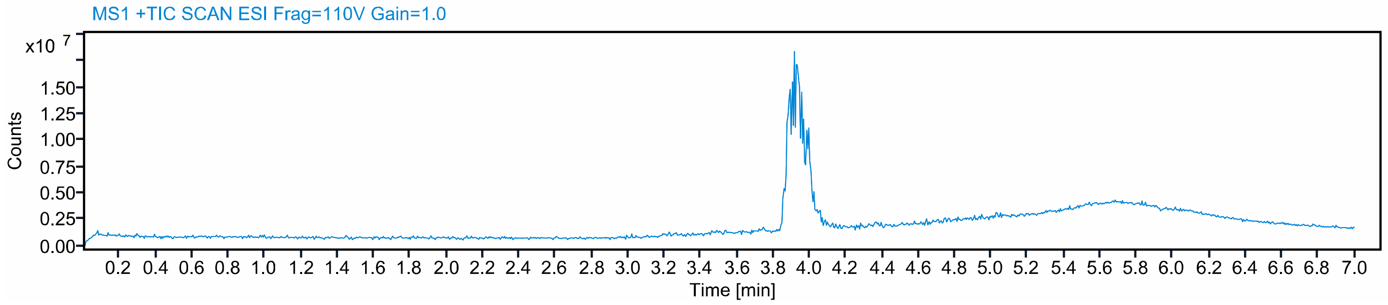

Signal: * VWD1A,Wavelength=254 nm [b]

RT [min]	Type	Width [min]	Area	Height	Area%
3.883	VB	0.51	1338.95	190.79	95.91
4.811	VB	0.43	57.13	3.03	4.09
		**Sum**	**1396.08**		

Signal: * VWD1B,Wavelength=210 nm [b]

RT [min]	Type	Width [min]	Area	Height	Area%
3.429	BB	0.27	81.94	14.02	1.61
3.695	BV	0.19	55.45	8.18	1.09
3.885	VB	0.61	4952.24	677.73	97.25
4.651	MM n	0.37	2.57	1.31	0.05
		**Sum**	**5092.20**		

Signal: MS1 +TIC SCAN ESI Frag=110V Gain=1.0

RT [min]	Type	Width [min]	Area	Height	Area%
3.914	MM m	0.35	103704475.10	16917174.16	100.00
		**Sum**	**103704475.10**		

Signal: MS1 -TIC SCAN ESI Frag=110V Gain=1.0

RT [min]	Type	Width [min]	Area	Height	Area%
3.198	MM m	0.15	112707.91	38002.72	8.36
3.922	MM m	0.29	1235525.01	199661.47	91.64
		**Sum**	**1348232.92**		

Compound 4

**Data file:**	AE-2-84-NH2-2.dx		
**Sequence Name:**	SingleSample	**Project Name:**	MeyersLab
**Sample name:**	AE-2-84-NH2-2	**Operator:**	SYSTEM (SYSTEM)
**Instrument:**	LCMS	**Injection date:**	2026-05-26 13:30:02-05:00
**Inj. volume:**	3.000 µL	**Location:**	91
**Acq. method:**	Meyerslab-Sept-24.amx	**Type:**	Sample
**Processing method:**	*Method Test-1.pmx	**Sample amount:**	0.00
**Manually modified:**	Manual Integration		

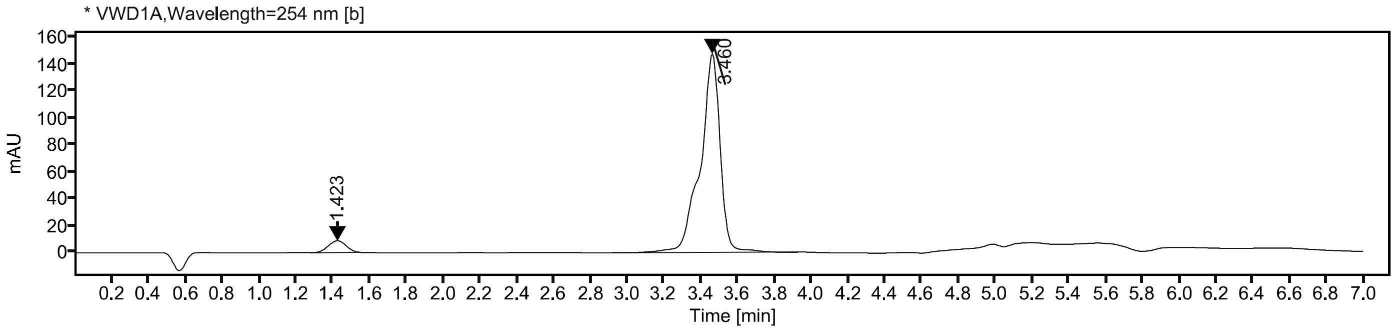

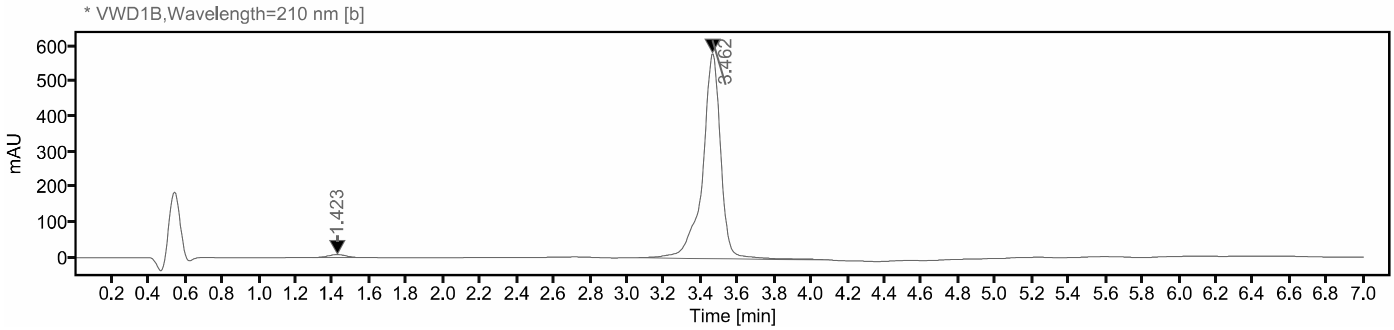

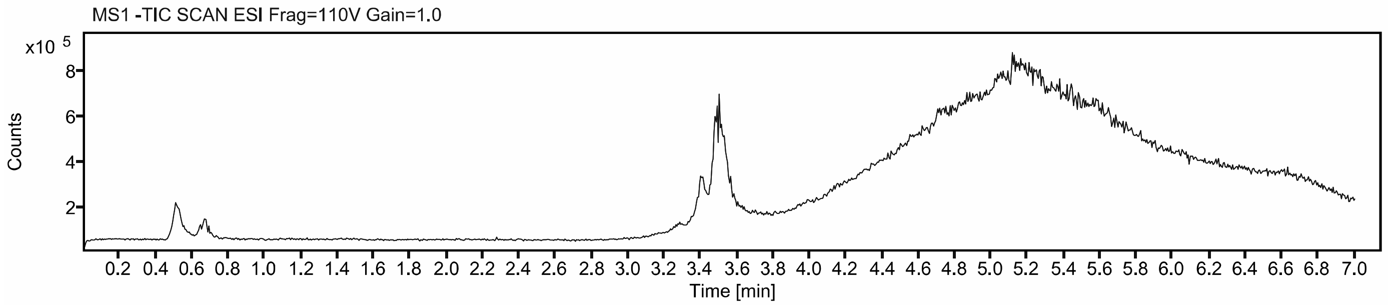

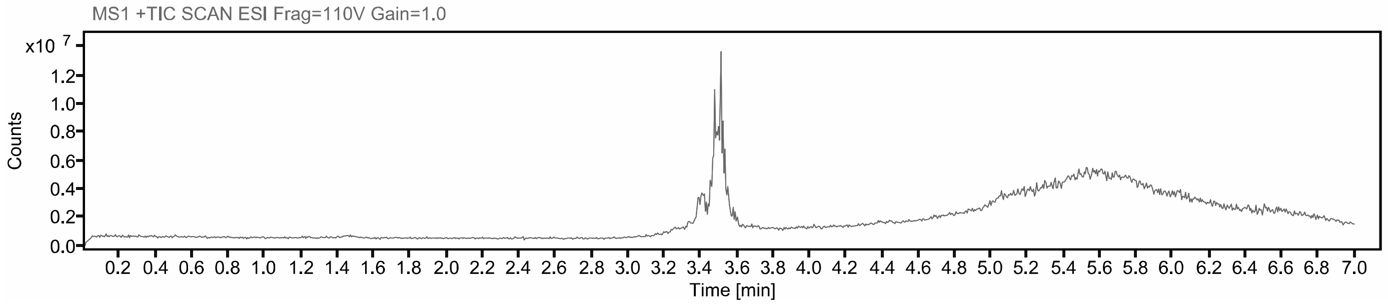

Signal: * VWD1A,Wavelength=254 nm [b]

RT [min]	Type	Width [min]	Area	Height	Area%
1.423	VM m	0.26	58.62	8.73	5.02
3.460	BM m	1.11	1107.96	147.74	94.98
		**Sum**	**1166.58**		

Signal: * VWD1B,Wavelength=210 nm [b]

RT [min]	Type	Width [min]	Area	Height	Area%
1.423	MM m	0.23	49.64	7.81	1.23
3.462	MM m	1.05	3996.20	581.82	98.77
		**Sum**	**4045.83**		

Signal: MS1 +TIC SCAN ESI Frag=110V Gain=1.0

RT [min]	Type	Width [min]	Area	Height	Area%
3.405	MM m	0.22	11949290.56	2739457.94	21.94
3.507	MM m	0.32	42520371.29	12676460.40	78.06
		**Sum**	**54469661.84**		

Signal: MS1 -TIC SCAN ESI Frag=110V Gain=1.0

RT [min]	Type	Width [min]	Area	Height	Area%
3.398	MM m	0.12	754605.39	212855.83	20.55
3.498	MM m	0.34	2917549.55	566007.60	79.45
		**Sum**	**3672154.94**		

Compound 5

**Data file:**	AE-2-85-NH2-8.dx		
**Sequence Name:**	SingleSample	**Project Name:**	MeyersLab
**Sample name:**	AE-2-85-NH2-8	**Operator:**	SYSTEM (SYSTEM)
**Instrument:**	LCMS	**Injection date:**	2026-05-21 15:18:59-05:00
**Inj. volume:**	3.000 µL	**Location:**	91
**Acq. method:**	Meyerslab-Sept-24.amx	**Type:**	Sample
**Processing method:**	*Method Test-1.pmx	**Sample amount:**	0.00
**Manually modified:**	Manual Integration		

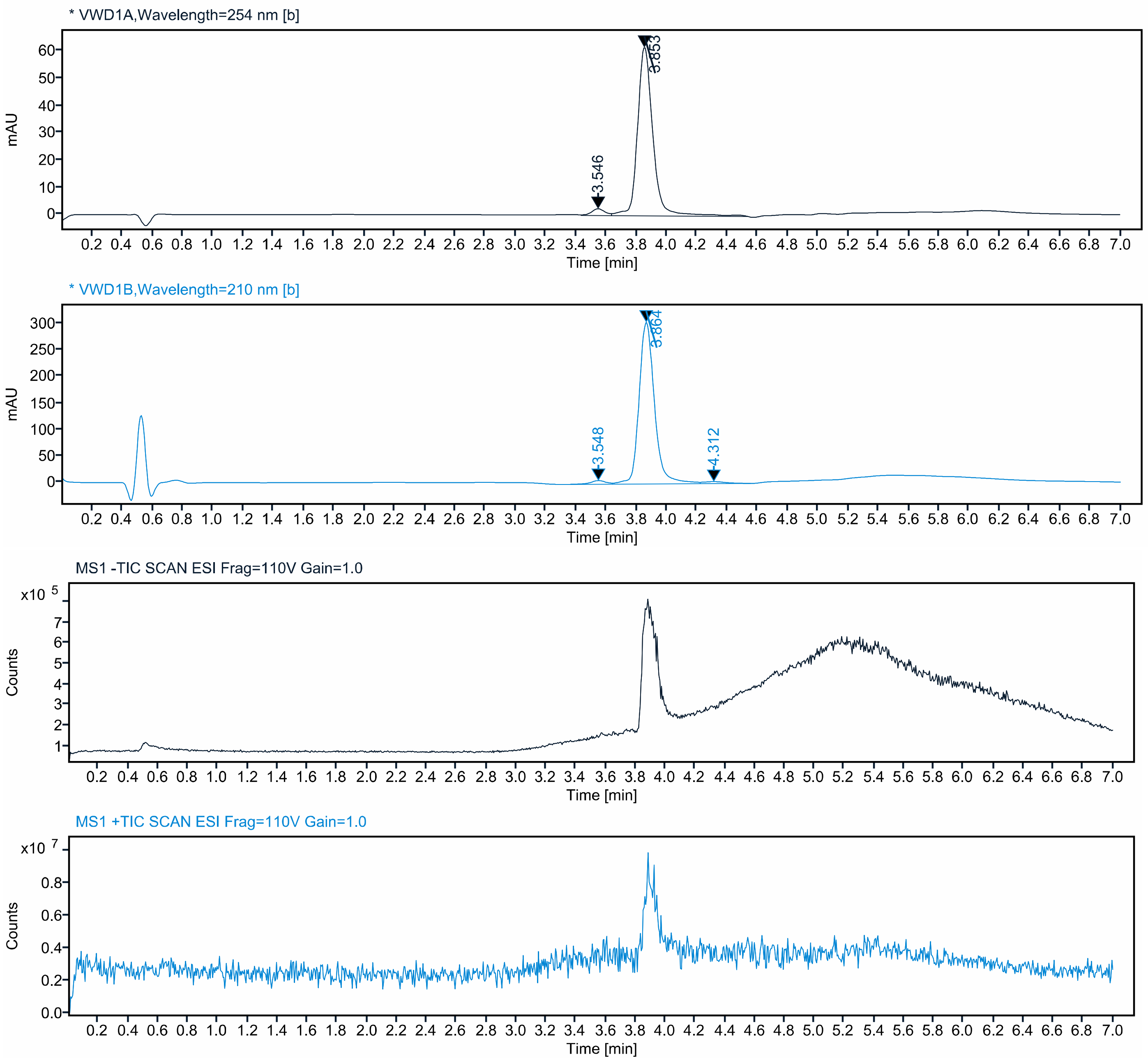

Signal: * VWD1A,Wavelength=254 nm [b]

RT [min]	Type	Width [min]	Area	Height	Area%
3.546	VM m	0.20	15.22	2.48	3.34
3.853	MM m	0.90	440.47	61.87	96.66
		**Sum**	**455.69**		

Signal: * VWD1B,Wavelength=210 nm [b]

RT [min]	Type	Width [min]	Area	Height	Area%
3.548	BV	0.31	49.57	6.88	2.21
3.864	VV	0.58	2156.97	303.72	95.97
4.312	VB	0.33	41.05	3.86	1.83
		**Sum**	**2247.60**		

Signal: MS1 +TIC SCAN ESI Frag=110V Gain=1.0

RT [min]	Type	Width [min]	Area	Height	Area%
3.887	MM m	0.37	39944469.99	7207048.74	100.00
		**Sum**	**39944469.99**		

Signal: MS1 -TIC SCAN ESI Frag=110V Gain=1.0

RT [min]	Type	Width [min]	Area	Height	Area%
3.882	MM m	0.36	3842645.88	611199.58	100.00
		**Sum**	**3842645.88**		

Compound 6

**Data file:**	ae-2-192-nh2-2hr.dx		
**Sequence Name:**	SingleSample	**Project Name:**	MeyersLab
**Sample name:**	ae-2-192-nh2-2hr	**Operator:**	SYSTEM (SYSTEM)
**Instrument:**	LCMS	**Injection date:**	2026-06-26 11:06:17-05:00
**Inj. volume:**	3.000 µL	**Location:**	91
**Acq. method:**	Meyerslab-Sept-24.amx	**Type:**	Sample
**Processing method:**	*Method Test-1.pmx	**Sample amount:**	0.00
**Manually modified:**	Manual Integration		

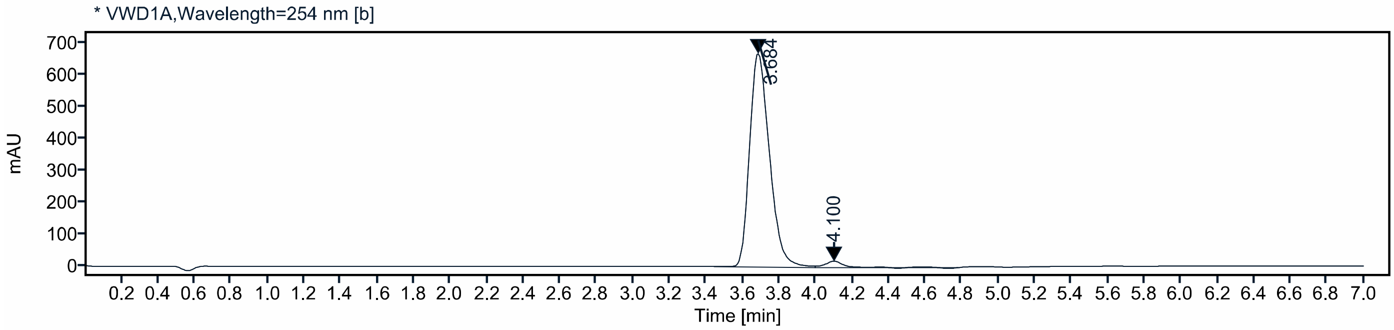

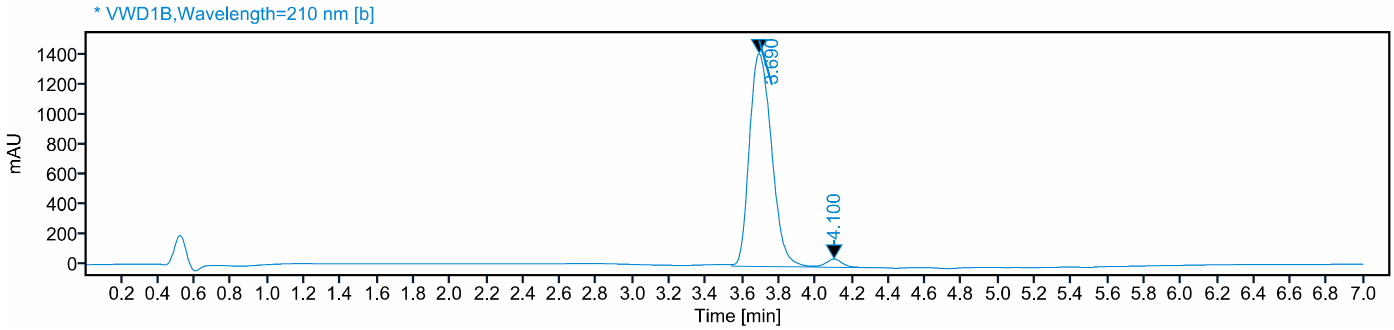

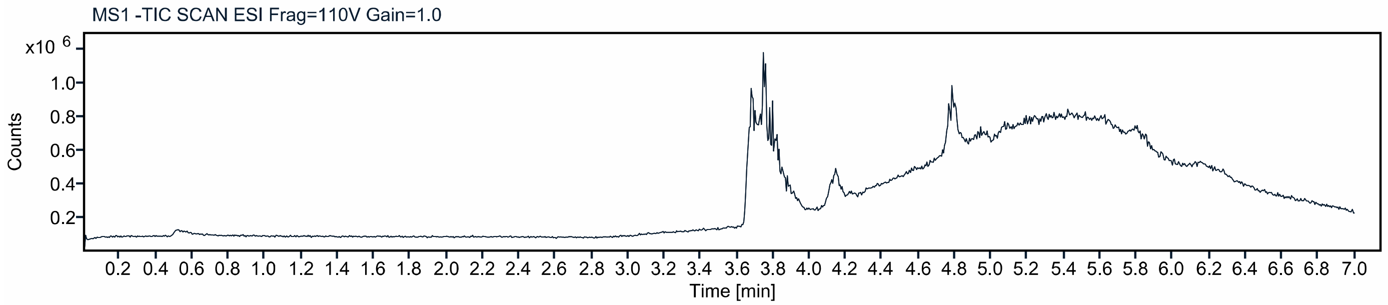

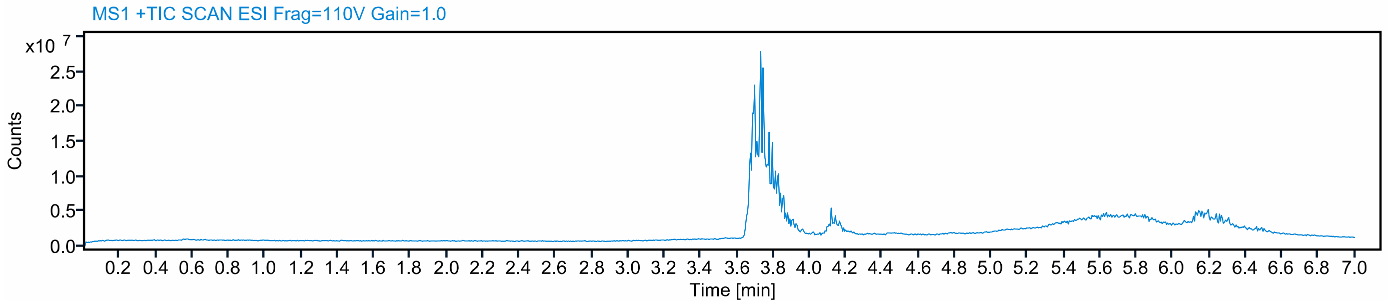

Signal: * VWD1A,Wavelength=254 nm [b]

RT [min]	Type	Width [min]	Area	Height	Area%
3.684	VV	0.55	5084.34	667.61	97.23
4.100	MM m	0.78	144.92	20.13	2.77
		**Sum**	**5229.26**		

Signal: * VWD1B,Wavelength=210 nm [b]

RT [min]	Type	Width [min]	Area	Height	Area%
3.690	VV	0.45	12070.17	1428.07	97.21
4.100	MM m	0.32	346.87	55.52	2.79
		**Sum**	**12417.04**		

Signal: MS1 +TIC SCAN ESI Frag=110V Gain=1.0

RT [min]	Type	Width [min]	Area	Height	Area%
3.732	MM m	0.43	151854778.50	26814255.29	93.22
4.120	MM m	0.25	11041065.47	3841834.89	6.78
		**Sum**	**162895843.98**		

Signal: MS1 -TIC SCAN ESI Frag=110V Gain=1.0

RT [min]	Type	Width [min]	Area	Height	Area%
3.747	MM m	0.41	7819642.20	1006208.49	81.42
4.142	MM m	0.27	754176.51	203290.64	7.85
4.782	MM m	0.16	1029904.11	378399.51	10.72
		**Sum**	**9603722.82**		

Compound 7

**Data file:**	ae-2-154-3-nh2-5.dx		
**Sequence Name:**	SingleSample	**Project Name:**	MeyersLab
**Sample name:**	ae-2-154-3-nh2-5	**Operator:**	SYSTEM (SYSTEM)
**Instrument:**	LCMS	**Injection date:**	2026-06-24 12:19:07-05:00
**Inj. volume:**	3.000 µL	**Location:**	91
**Acq. method:**	Meyerslab-Sept-24.amx	**Type:**	Sample
**Processing method:**	*Method Test-1.pmx	**Sample amount:**	0.00
**Manually modified:**	Manual Integration		

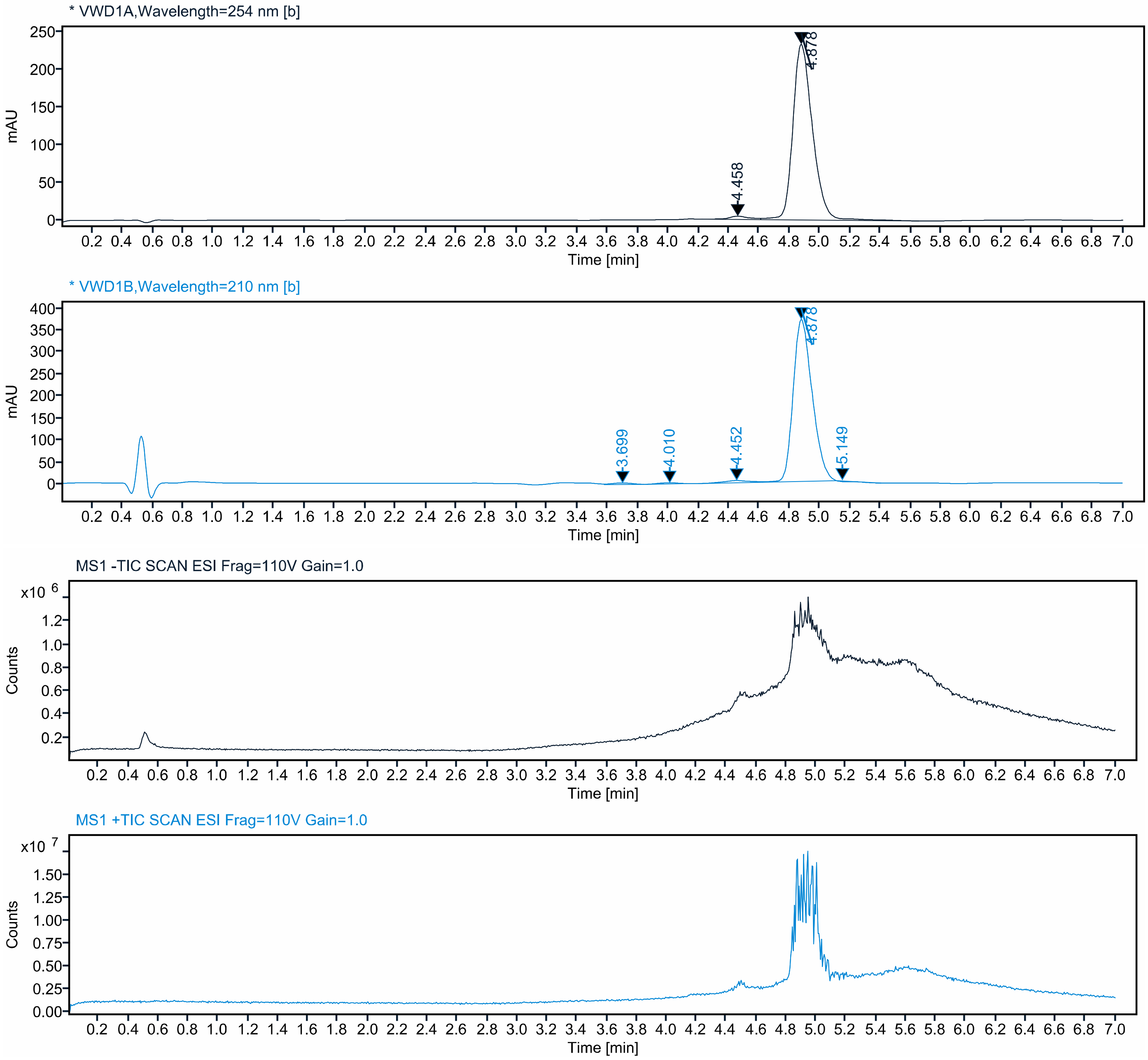

Signal: * VWD1A,Wavelength=254 nm [b]

RT [min]	Type	Width [min]	Area	Height	Area%
4.458	BV	0.39	42.05	4.45	1.95
4.878	VB	1.04	2114.02	234.40	98.05
		**Sum**	**2156.07**		

Signal: * VWD1B,Wavelength=210 nm [b]

RT [min]	Type	Width [min]	Area	Height	Area%
3.699	VB	0.26	29.82	3.26	0.90
4.010	BV	0.27	24.86	2.77	0.75
4.452	BM m	0.43	58.70	5.05	1.77
4.878	MM m	0.49	3199.24	368.15	96.24
5.149	MB n	0.29	11.78	1.65	0.35
		**Sum**	**3324.39**		

Signal: MS1 +TIC SCAN ESI Frag=110V Gain=1.0

RT [min]	Type	Width [min]	Area	Height	Area%
4.498	MM m	0.30	3970486.64	1061732.73	3.50
4.943	MM m	0.50	109516600.10	14122440.28	96.50
		**Sum**	**113487086.74**		

Signal: MS1 -TIC SCAN ESI Frag=110V Gain=1.0

RT [min]	Type	Width [min]	Area	Height	Area%
4.679	MM m	0.30	414545.24	10141.20	6.76
4.947	MM m	0.43	5718160.81	635958.20	93.24
		**Sum**	**6132706.05**		

Compound 8

**Data file:**	ae-2-193-2-3-5.dx		
**Sequence Name:**	SingleSample	**Project Name:**	MeyersLab
**Sample name:**	ae-2-193-2-3-5	**Operator:**	SYSTEM (SYSTEM)
**Instrument:**	LCMS	**Injection date:**	2026-06-24 15:25:36-05:00
**Inj. volume:**	3.000 µL	**Location:**	92
**Acq. method:**	Meyerslab-Sept-24.amx	**Type:**	Sample
**Processing method:**	*Method Test-1.pmx	**Sample amount:**	0.00
**Manually modified:**	Manual Integration		

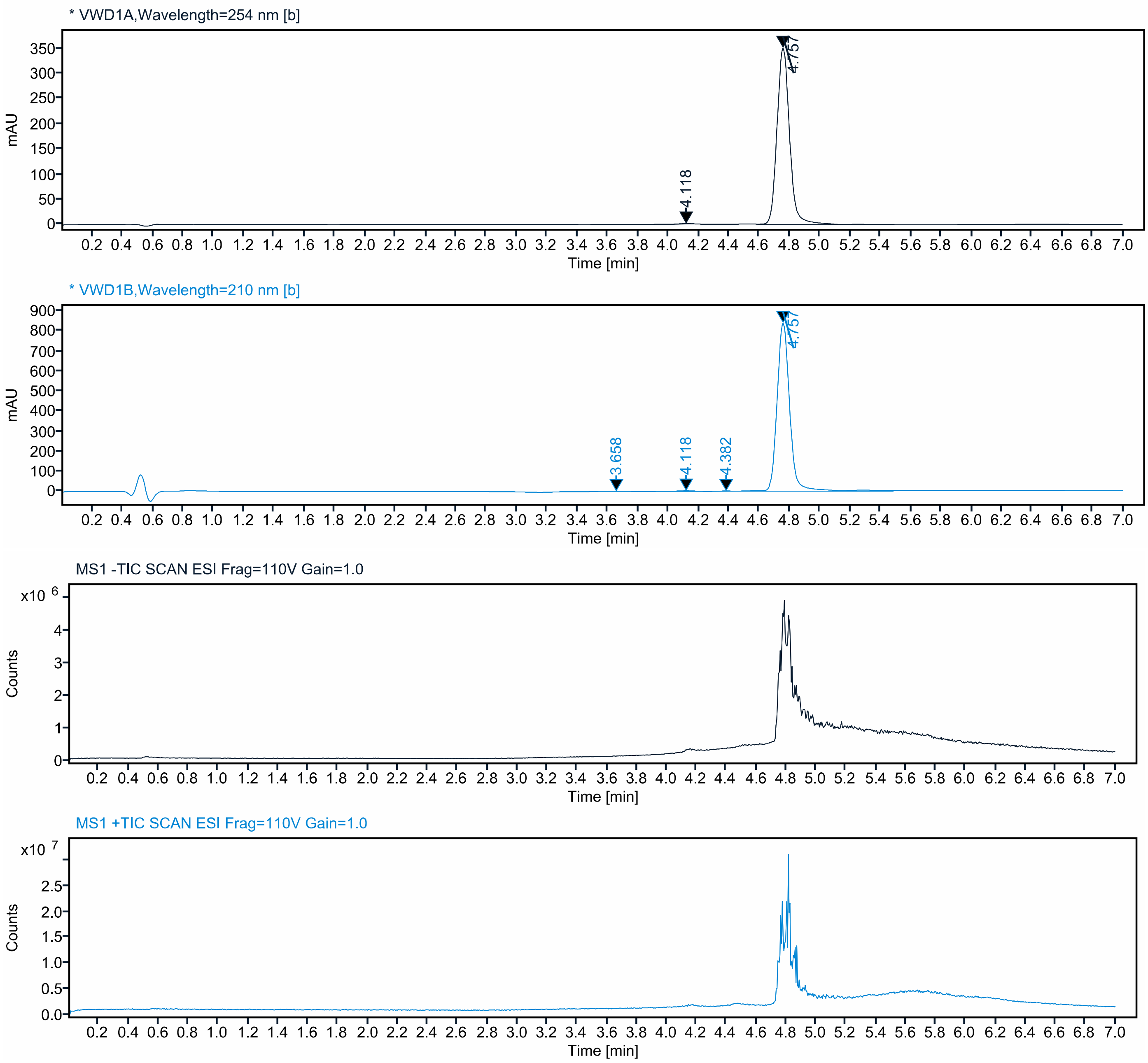

Signal: * VWD1A,Wavelength=254 nm [b]

RT [min]	Type	Width [min]	Area	Height	Area%
4.118	MM m	0.13	4.25	0.95	0.21
4.757	VB	0.56	2020.81	351.37	99.79
		**Sum**	**2025.06**		

Signal: * VWD1B,Wavelength=210 nm [b]

RT [min]	Type	Width [min]	Area	Height	Area%
3.658	MM m	0.33	18.15	1.62	0.37
4.118	MM m	0.41	34.25	3.34	0.69
4.382	MM m	0.19	10.65	1.58	0.21
4.757	VV m	1.04	4894.38	837.58	98.73
		**Sum**	**4957.43**		

Signal: MS1 +TIC SCAN ESI Frag=110V Gain=1.0

RT [min]	Type	Width [min]	Area	Height	Area%
4.813	MM m	0.39	114761550.53	28953205.44	100.00
		**Sum**	**114761550.53**		

Signal: MS1 -TIC SCAN ESI Frag=110V Gain=1.0

RT [min]	Type	Width [min]	Area	Height	Area%
4.785	MM m	0.41	25873875.94	4261603.78	100.00
		**Sum**	**25873875.94**		

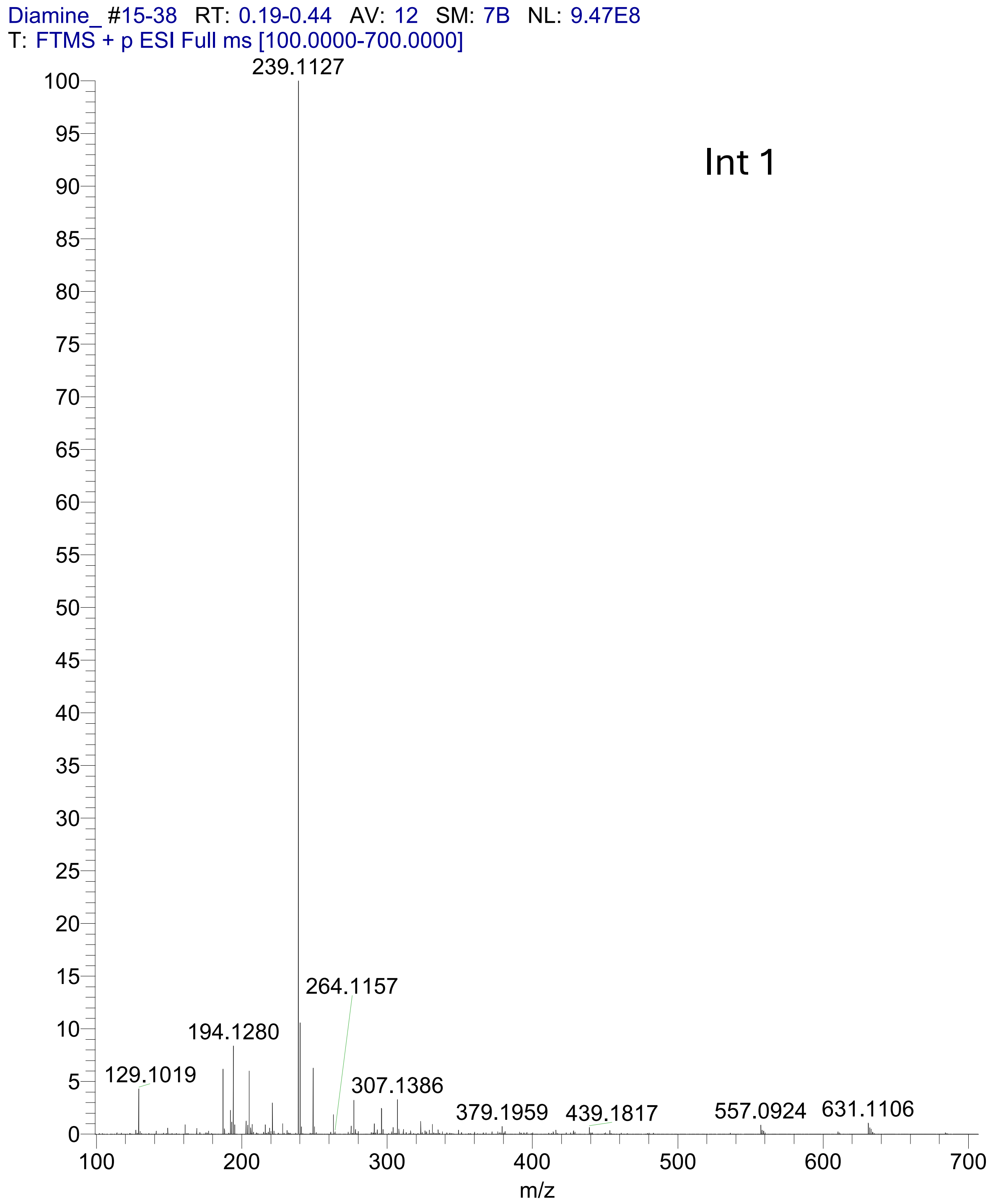

Diamine_ #15-38 RT: 0.19-0.44 AV: 12 SM: 7B NL: 9.47E8
T: FTMS + p ESI Full ms [100.0000-700.0000]
239.1127
Int 1
264.1157
194.1280
129.1019
307.1386
379.1959
439.1817
557.0924
631.1106
m/z

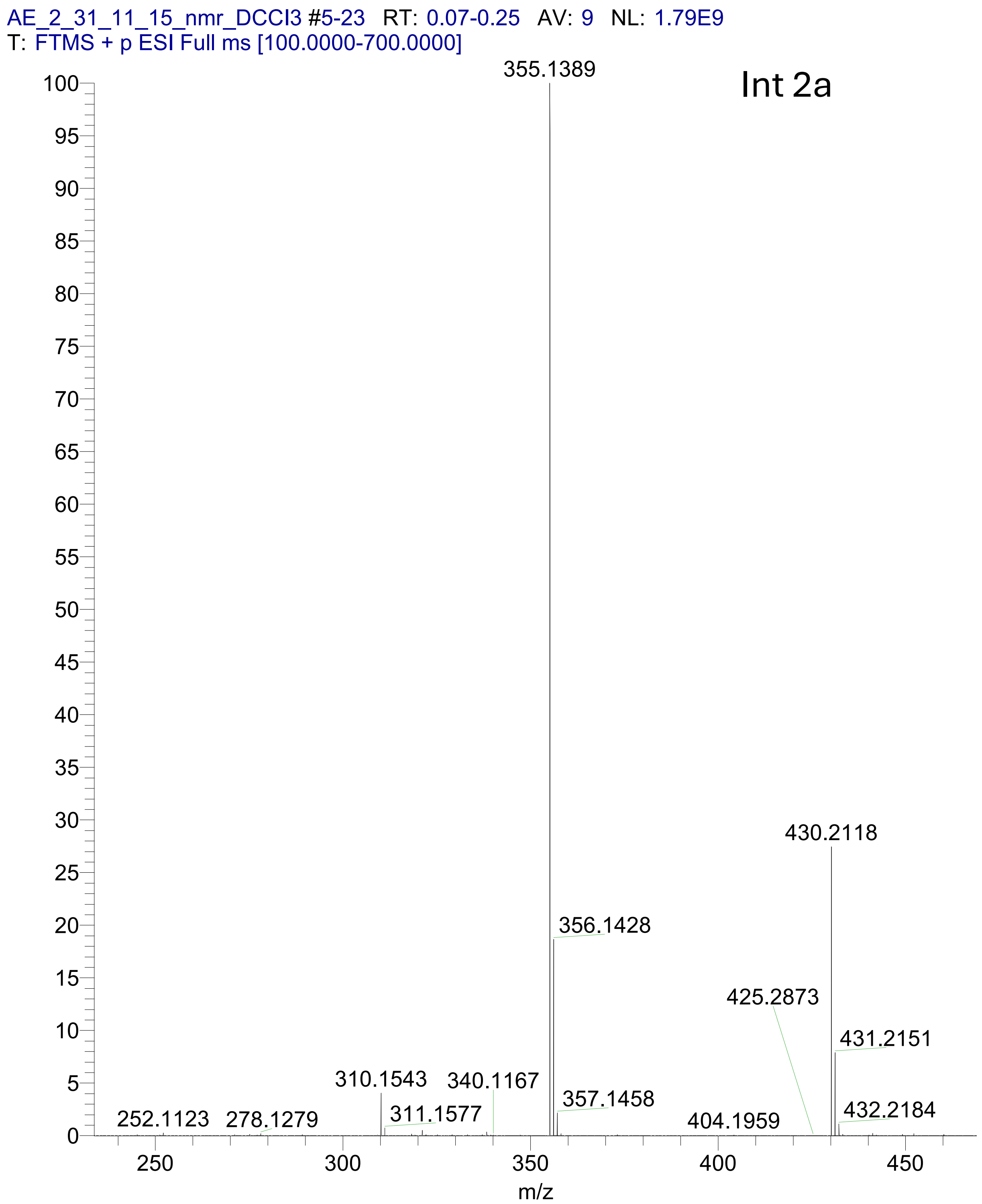

AE_2_31_11_15_nmr_DCCl3 #5-23 RT: 0.07-0.25 AV: 9 NL: 1.79E9
T: FTMS + p ESI Full ms [100.0000-700.0000]
Int 2a
355.1389
430.2118
356.1428
425.2873
431.2151
310.1543
340.1167
357.1458
252.1123
278.1279
311.1577
404.1959
432.2184
m/z

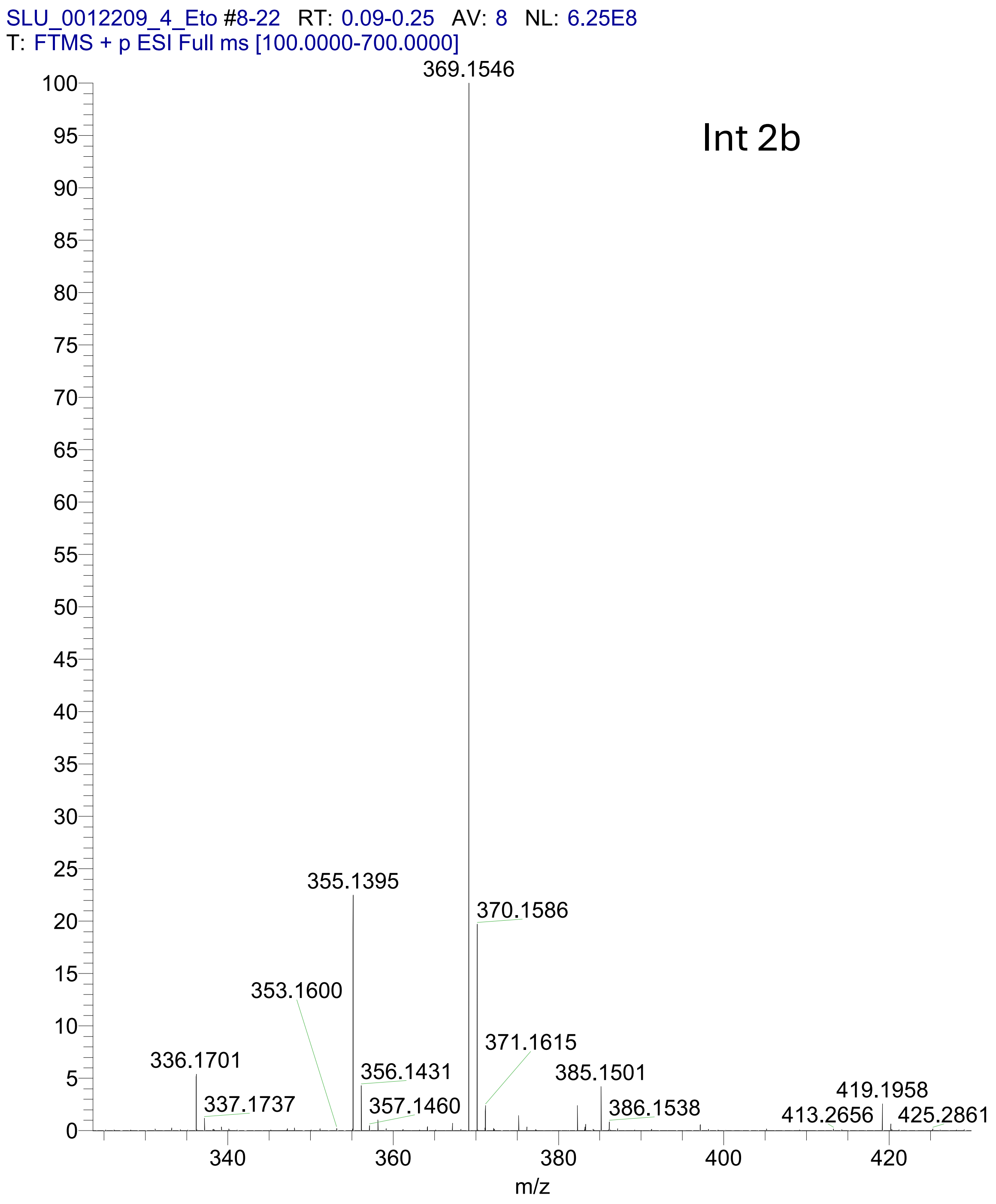
SLU_0012209_4_Eto #8-22 RT: 0.09-0.25 AV: 8 NL: 6.25E8
T: FTMS + p ESI Full ms [100.0000-700.0000]
Int 2b
369.1546
355.1395
370.1586
353.1600
371.1615
336.1701
356.1431
385.1501
419.1958
337.1737
357.1460
386.1538
413.2656
425.2861
m/z

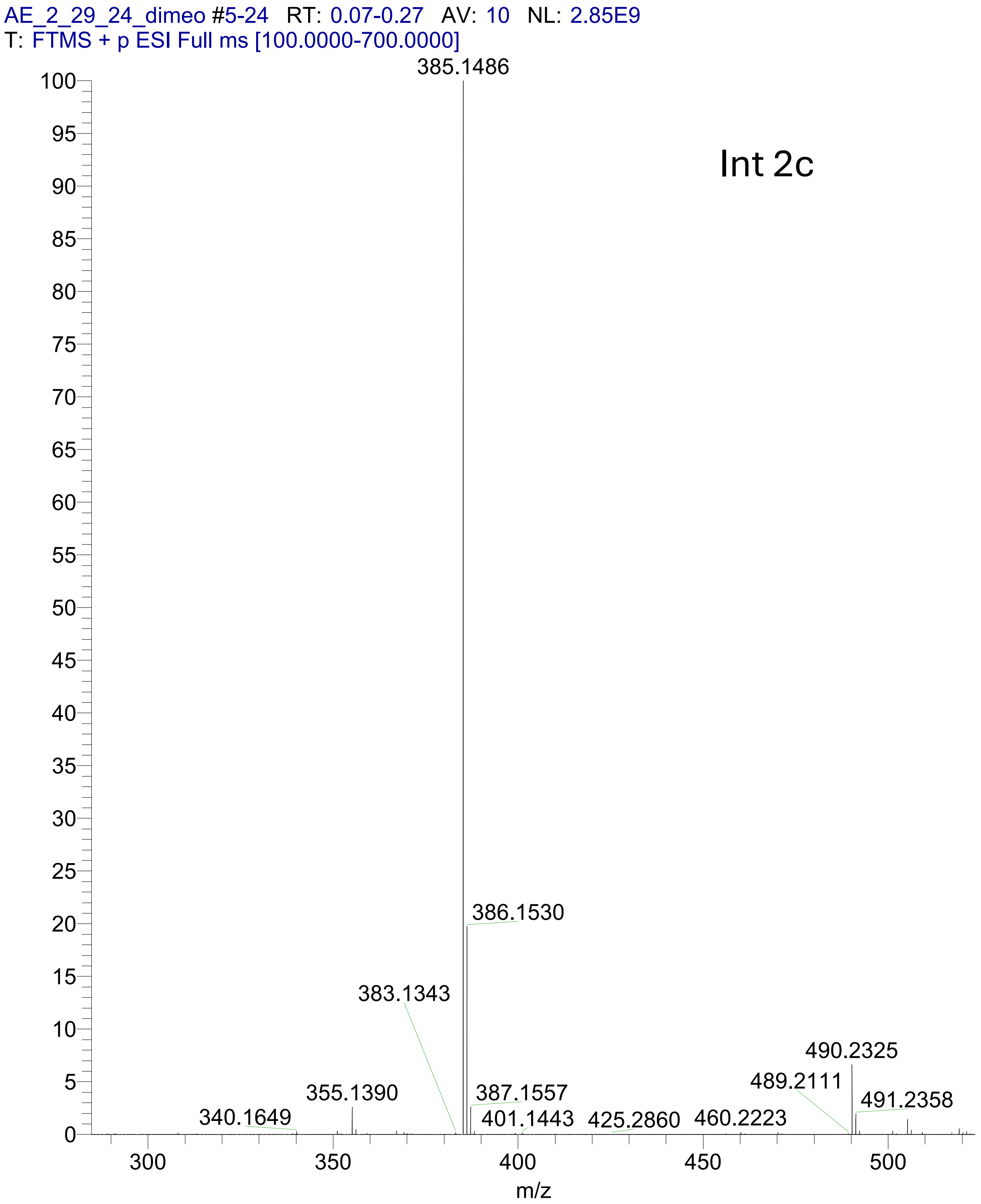
AE_2_29_24_dimeo #5-24 RT: 0.07-0.27 AV: 10 NL: 2.85E9
T: FTMS + p ESI Full ms [100.0000-700.0000]
Int 2c
385.1486
386.1530
383.1343
355.1390
340.1649
387.1557
401.1443
425.2860
460.2223
489.2111
490.2325
491.2358
m/z

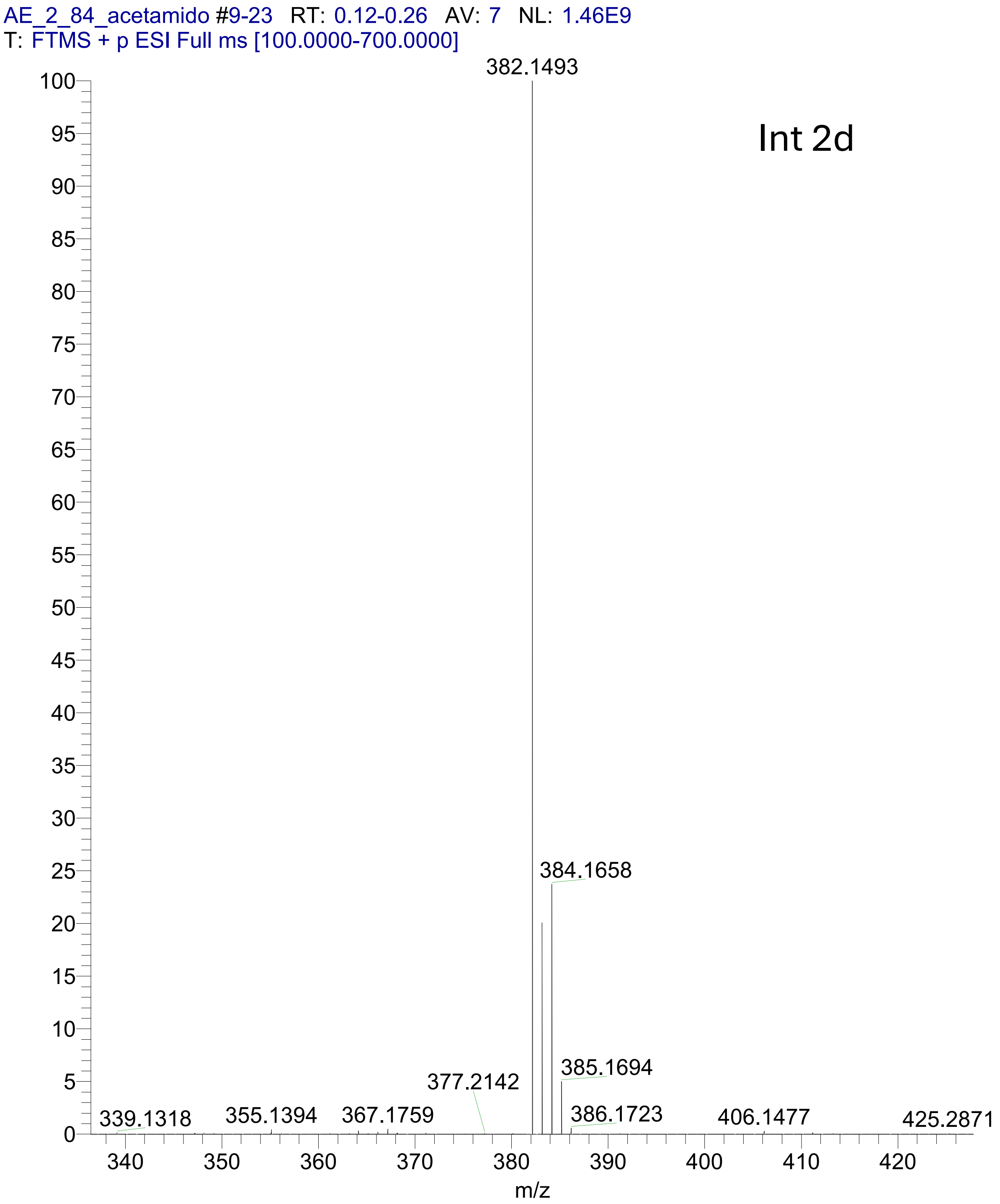
AE_2_84_acetamido #9-23 RT: 0.12-0.26 AV: 7 NL: 1.46E9
T: FTMS + p ESI Full ms [100.0000-700.0000]
Int 2d
382.1493
384.1658
385.1694
377.2142
386.1723
339.1318
355.1394
367.1759
406.1477
425.2871
m/z

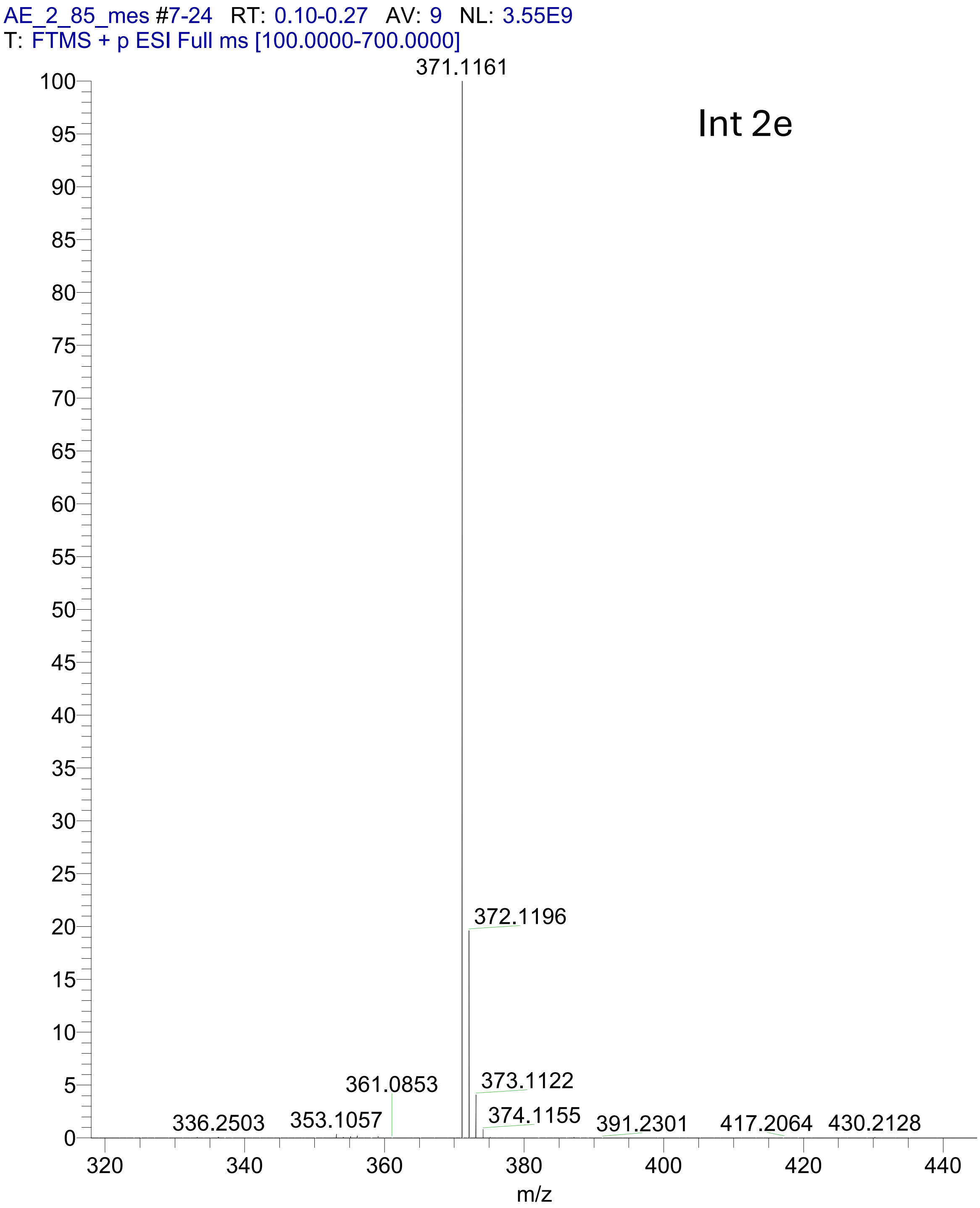
AE_2_85_mes #7-24 RT: 0.10-0.27 AV: 9 NL: 3.55E9
T: FTMS + p ESI Full ms [100.0000-700.0000]
Int 2e
371.1161
372.1196
373.1122
374.1155
361.0853
353.1057
336.2503
391.2301
417.2064
430.2128
m/z

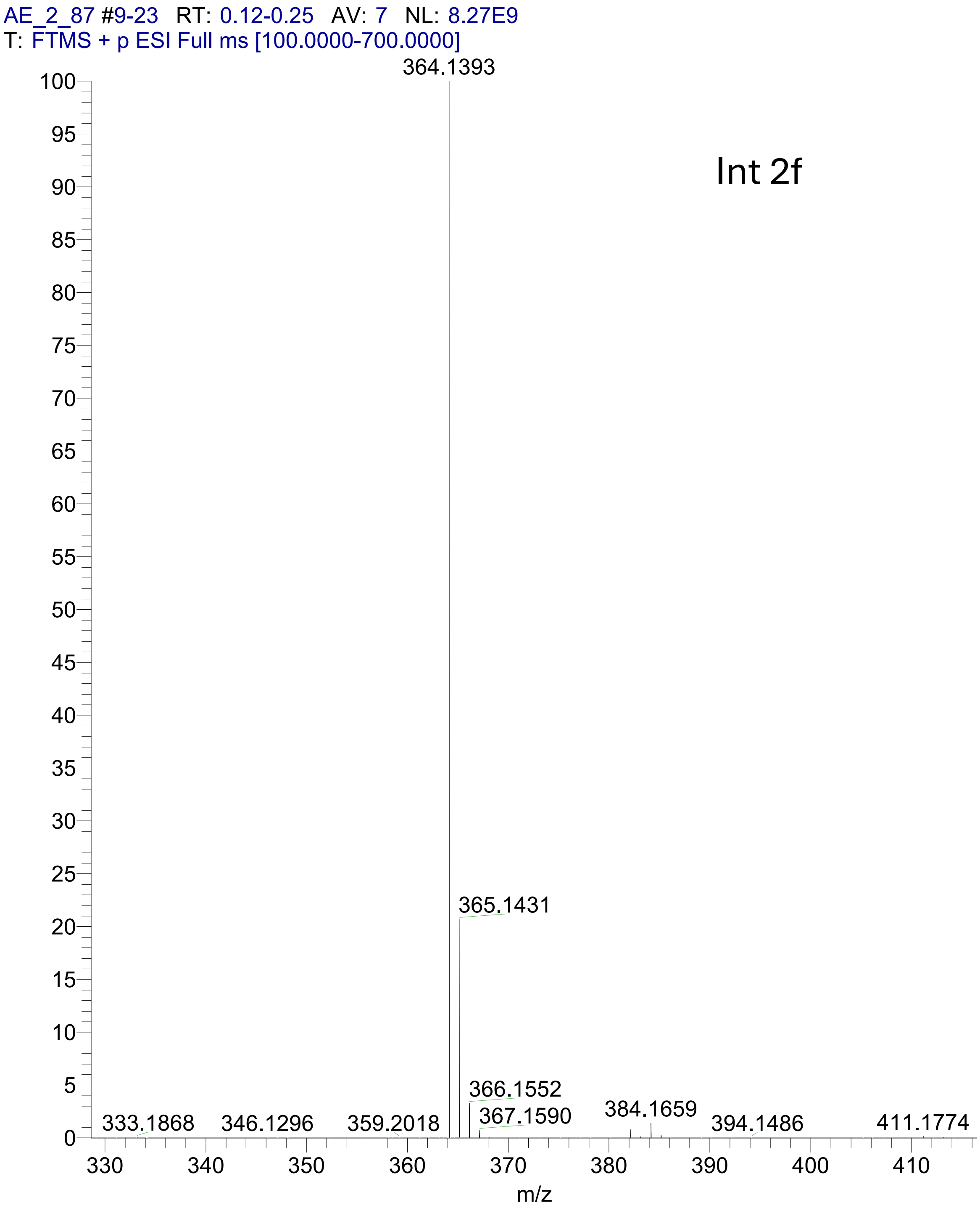

AE_2_87 #9-23 RT: 0.12-0.25 AV: 7 NL: 8.27E9
T: FTMS + p ESI Full ms [100.0000-700.0000]
Int 2f
364.1393
365.1431
366.1552
367.1590
384.1659
333.1868
346.1296
359.2018
394.1486
411.1774
m/z

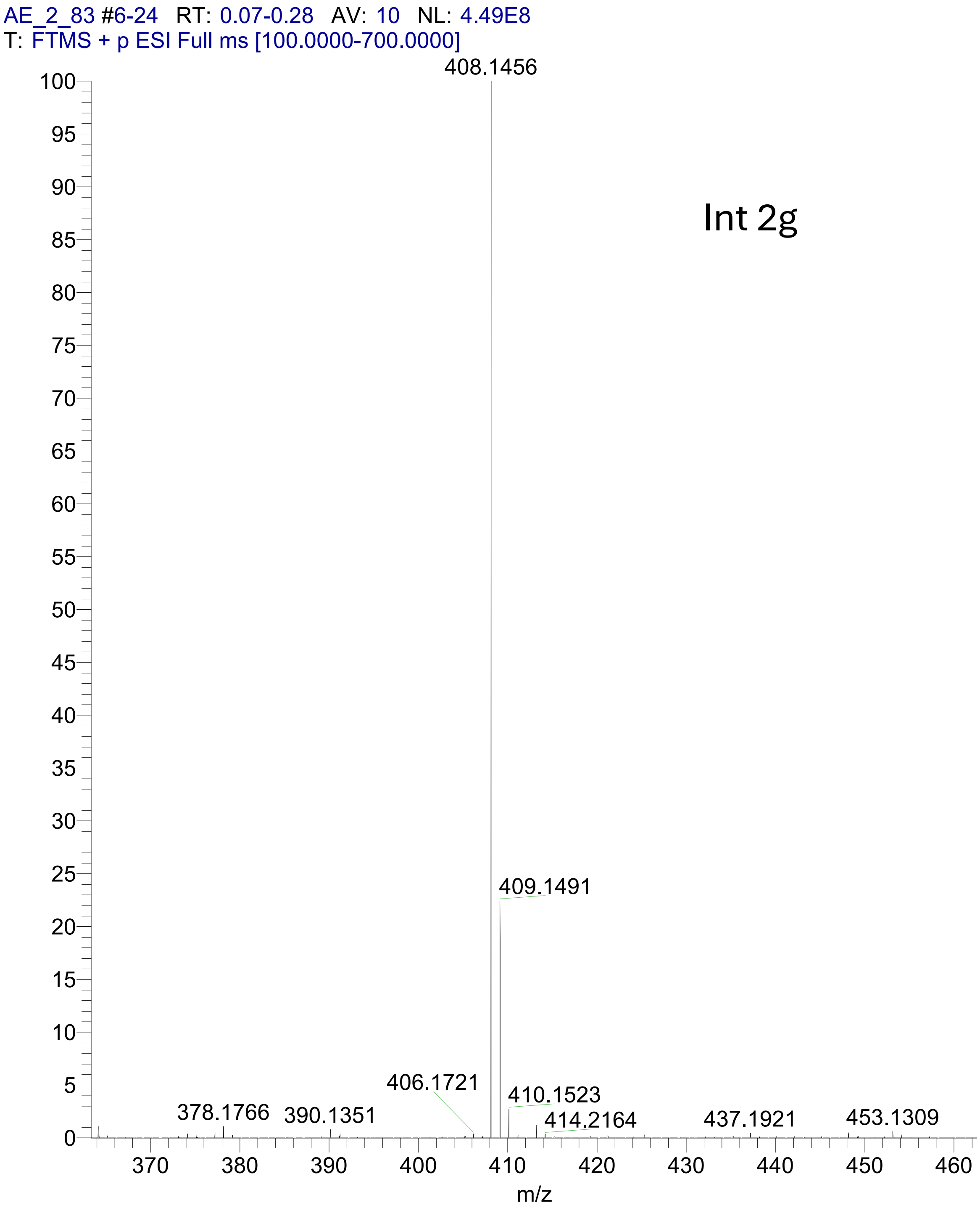
AE_2_83 #6-24 RT: 0.07-0.28 AV: 10 NL: 4.49E8
T: FTMS + p ESI Full ms [100.0000-700.0000]
Int 2g
408.1456
409.1491
406.1721
410.1523
414.2164
378.1766
390.1351
437.1921
453.1309
m/z

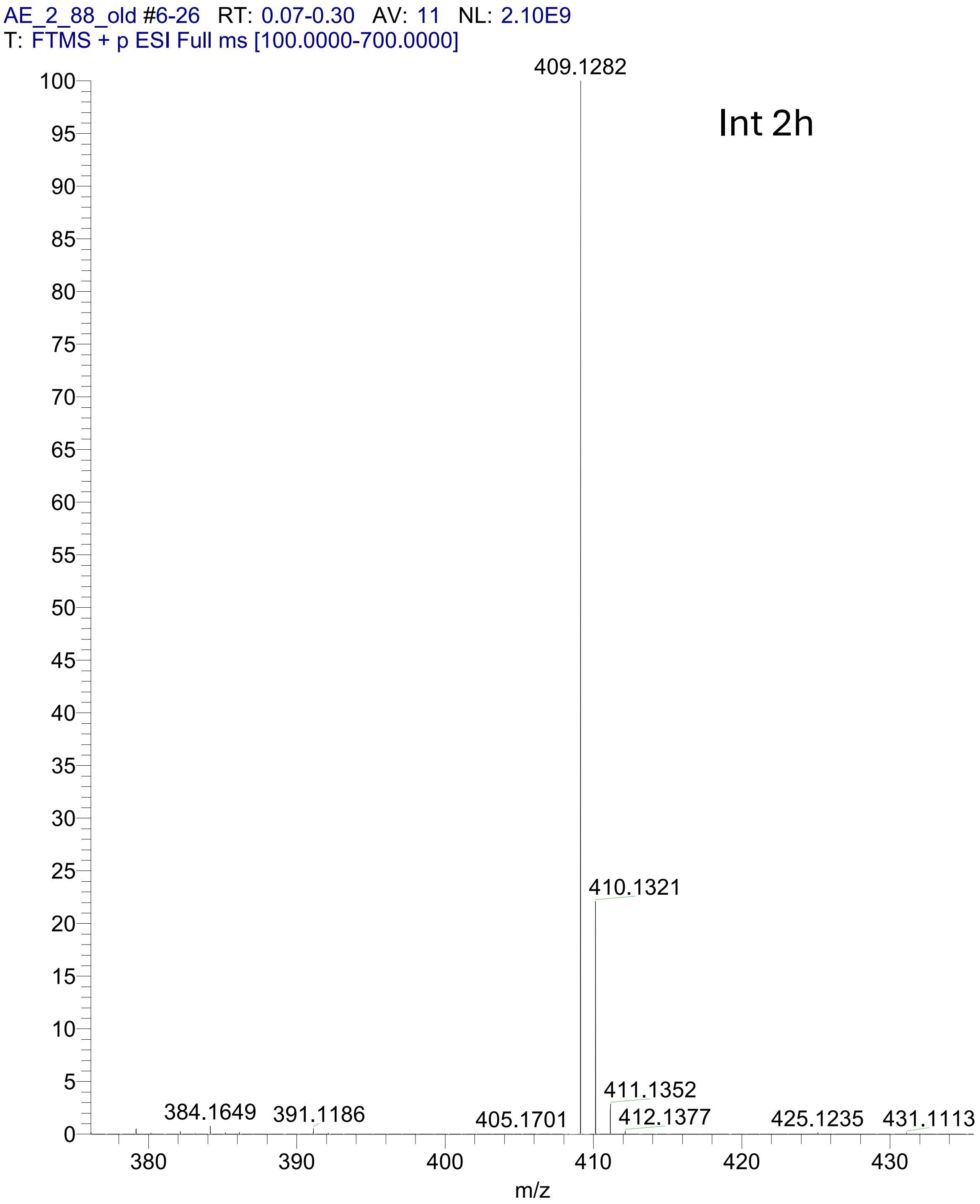
AE_2_88_old #6-26 RT: 0.07-0.30 AV: 11 NL: 2.10E9
T: FTMS + p ESI Full ms [100.0000-700.0000]
Int 2h
409.1282
410.1321
411.1352
412.1377
384.1649
391.1186
405.1701
425.1235
431.1113
m/z

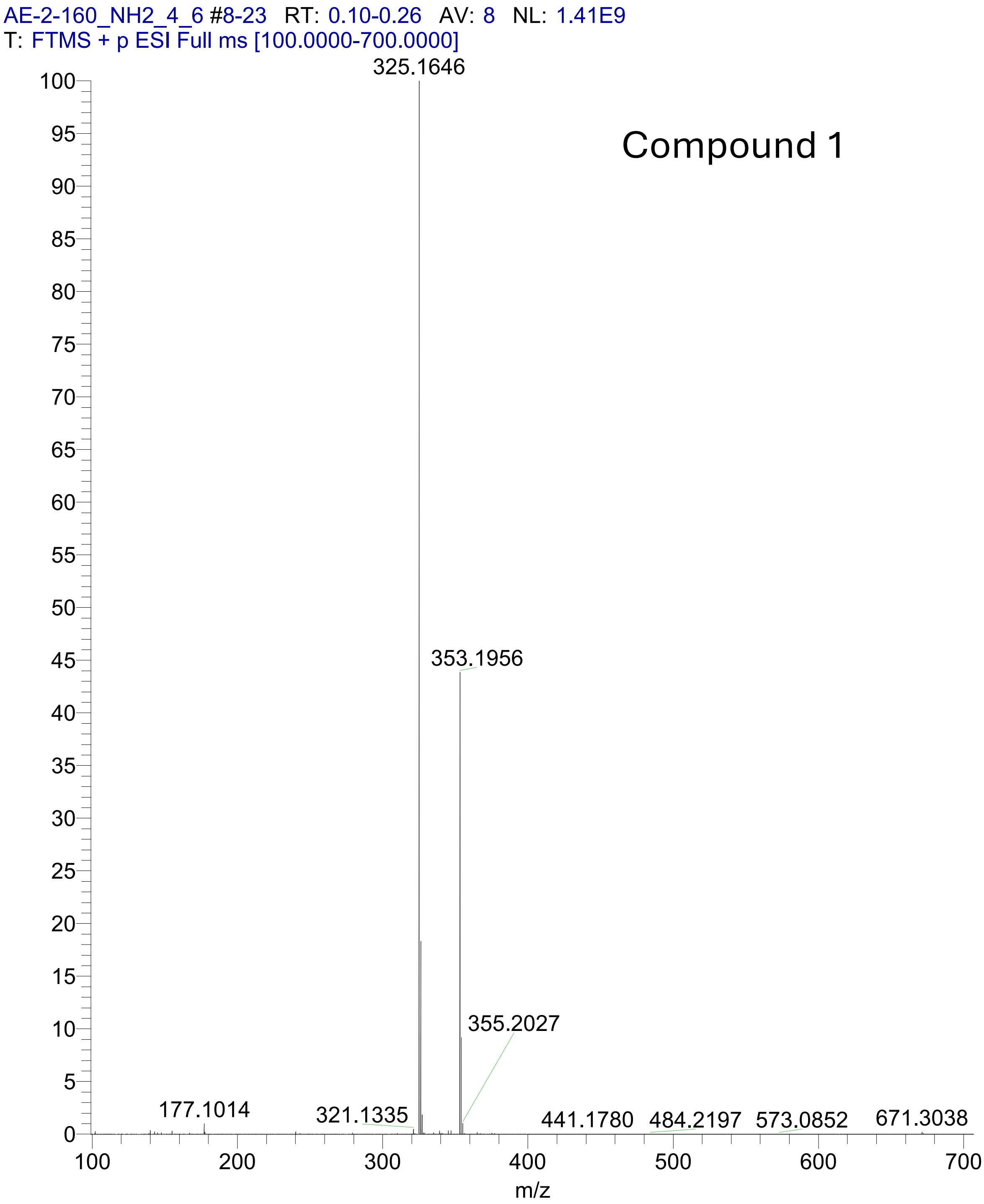
AE-2-160_NH2_4_6 #8-23 RT: 0.10-0.26 AV: 8 NL: 1.41E9
T: FTMS + p ESI Full ms [100.0000-700.0000]
Compound 1
325.1646
353.1956
355.2027
177.1014
321.1335
441.1780
484.2197
573.0852
671.3038
m/z

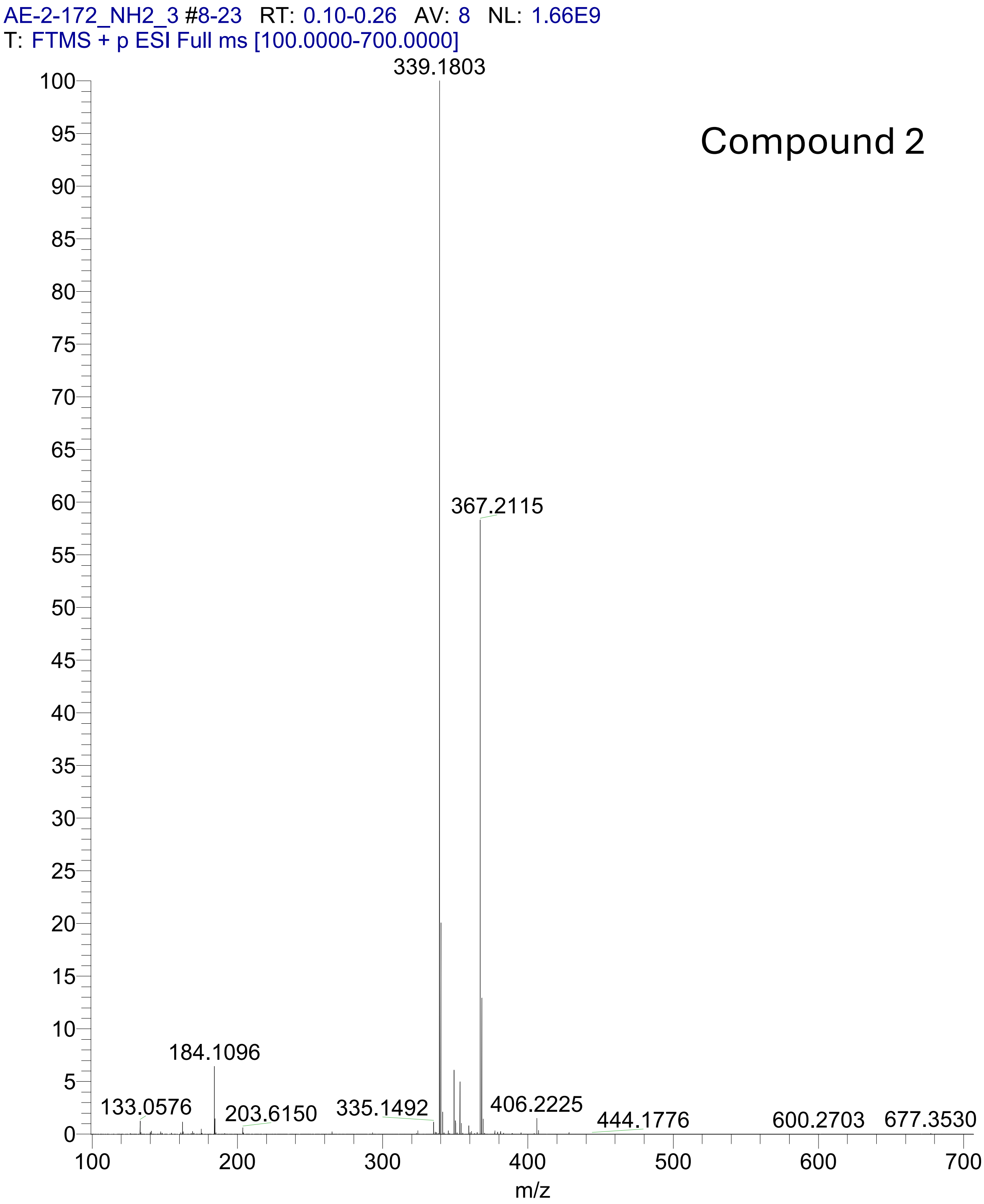

AE-2-172_NH2_3 #8-23 RT: 0.10-0.26 AV: 8 NL: 1.66E9
T: FTMS + p ESI Full ms [100.0000-700.0000]
Compound 2
339.1803
367.2115
184.1096
133.0576
203.6150
335.1492
406.2225
444.1776
600.2703
677.3530
m/z

AE-2-169_NH2_2 #8-23 RT: 0.10-0.26 AV: 8 NL: 1.79E9
T: FTMS + p ESI Full ms [100.0000-700.0000]

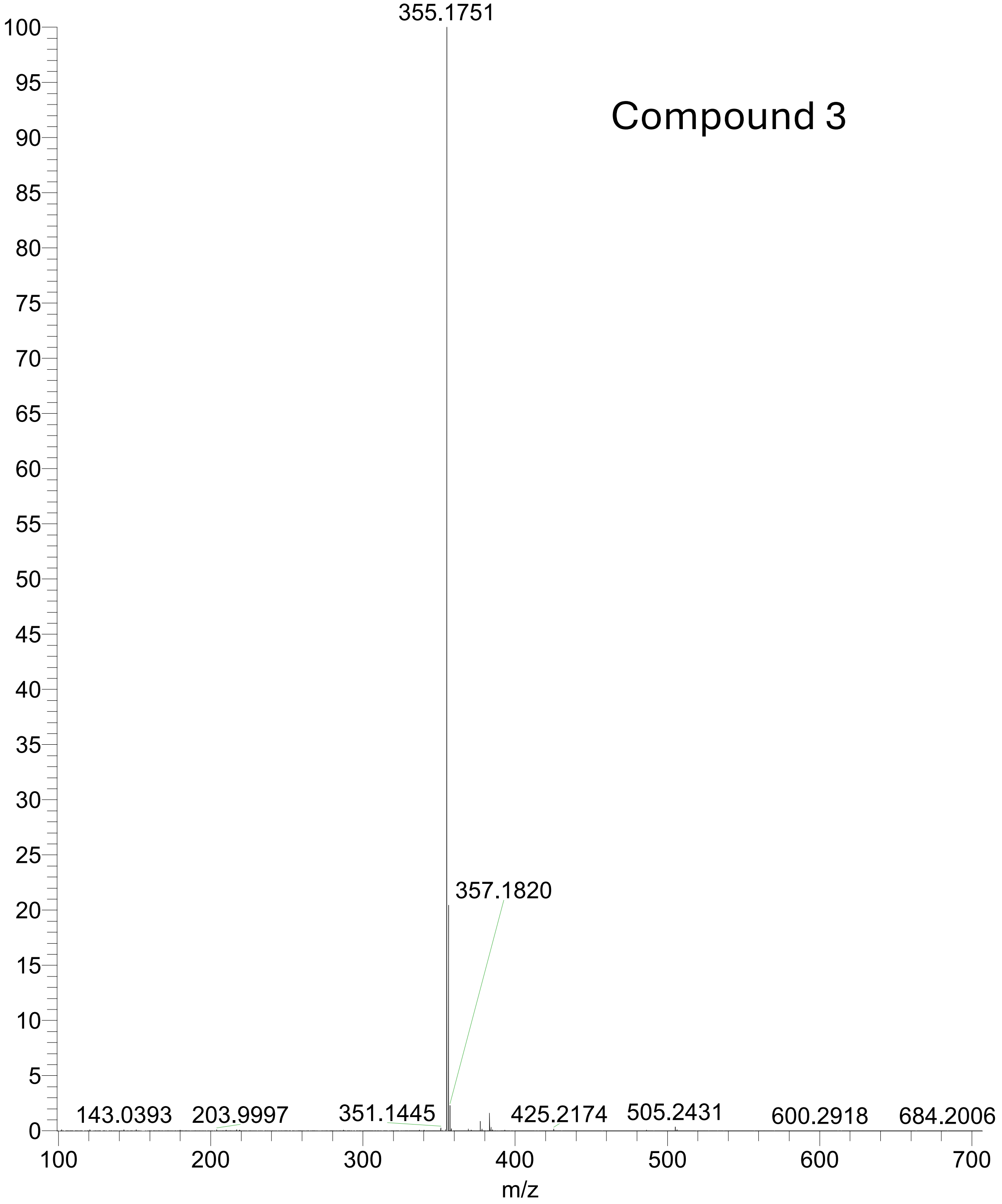

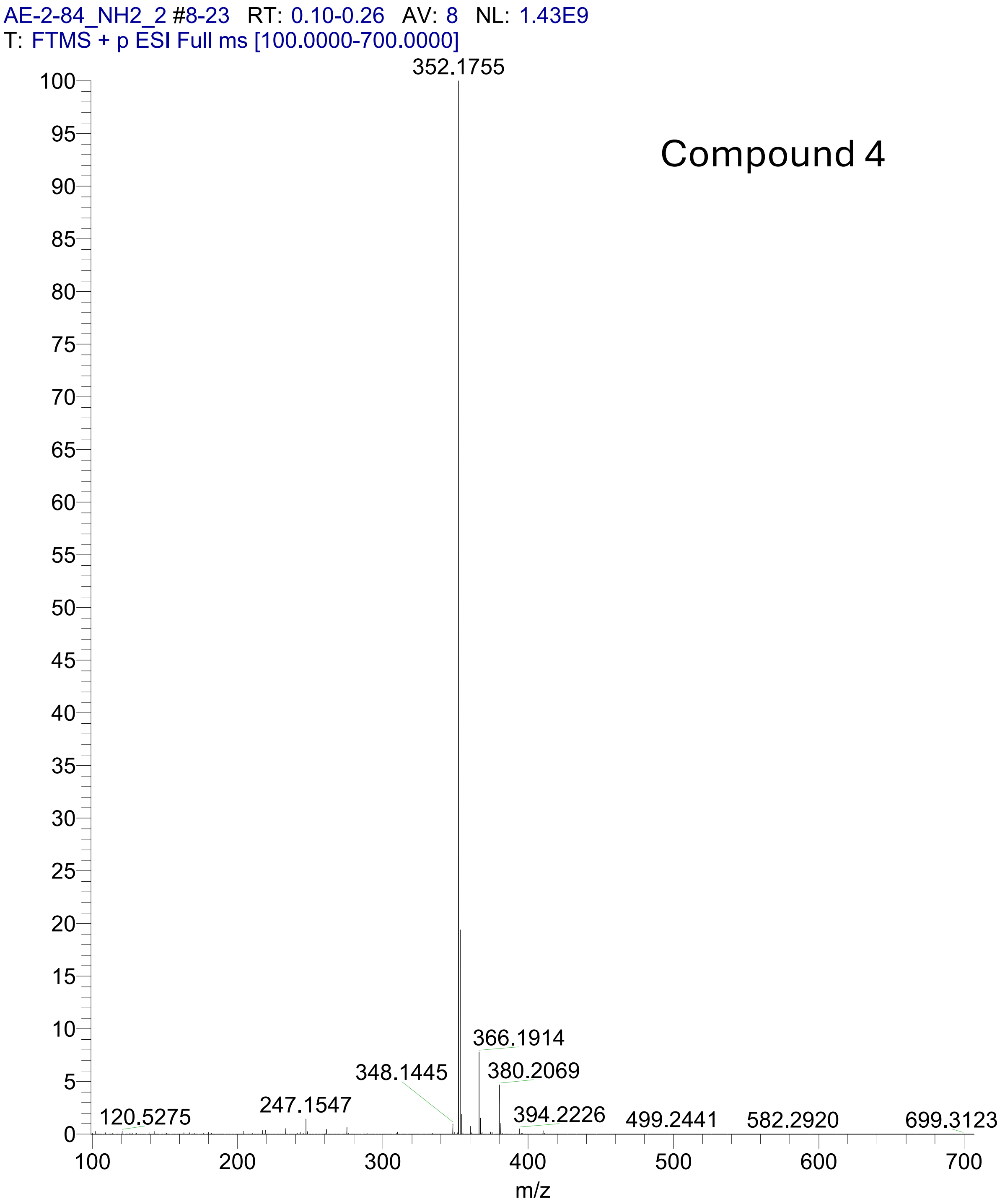

AE-2-84_NH2_2 #8-23 RT: 0.10-0.26 AV: 8 NL: 1.43E9
T: FTMS + p ESI Full ms [100.0000-700.0000]
Compound 4
352.1755
366.1914
380.2069
348.1445
247.1547
120.5275
394.2226
499.2441
582.2920
699.3123
m/z

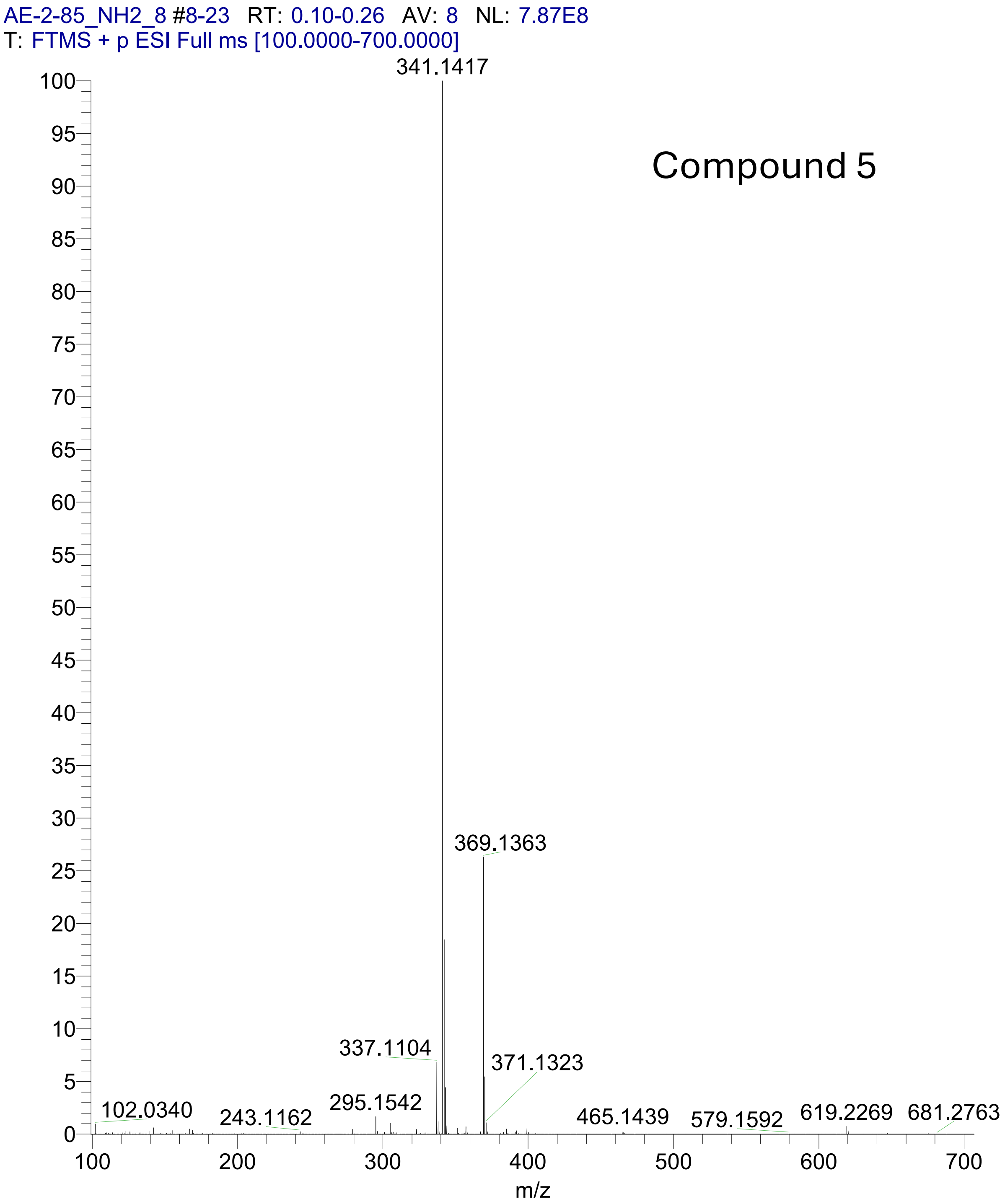
AE-2-85_NH2_8 #8-23 RT: 0.10-0.26 AV: 8 NL: 7.87E8
T: FTMS + p ESI Full ms [100.0000-700.0000]
Compound 5
341.1417
369.1363
337.1104
371.1323
295.1542
102.0340
243.1162
465.1439
579.1592
619.2269
681.2763
m/z

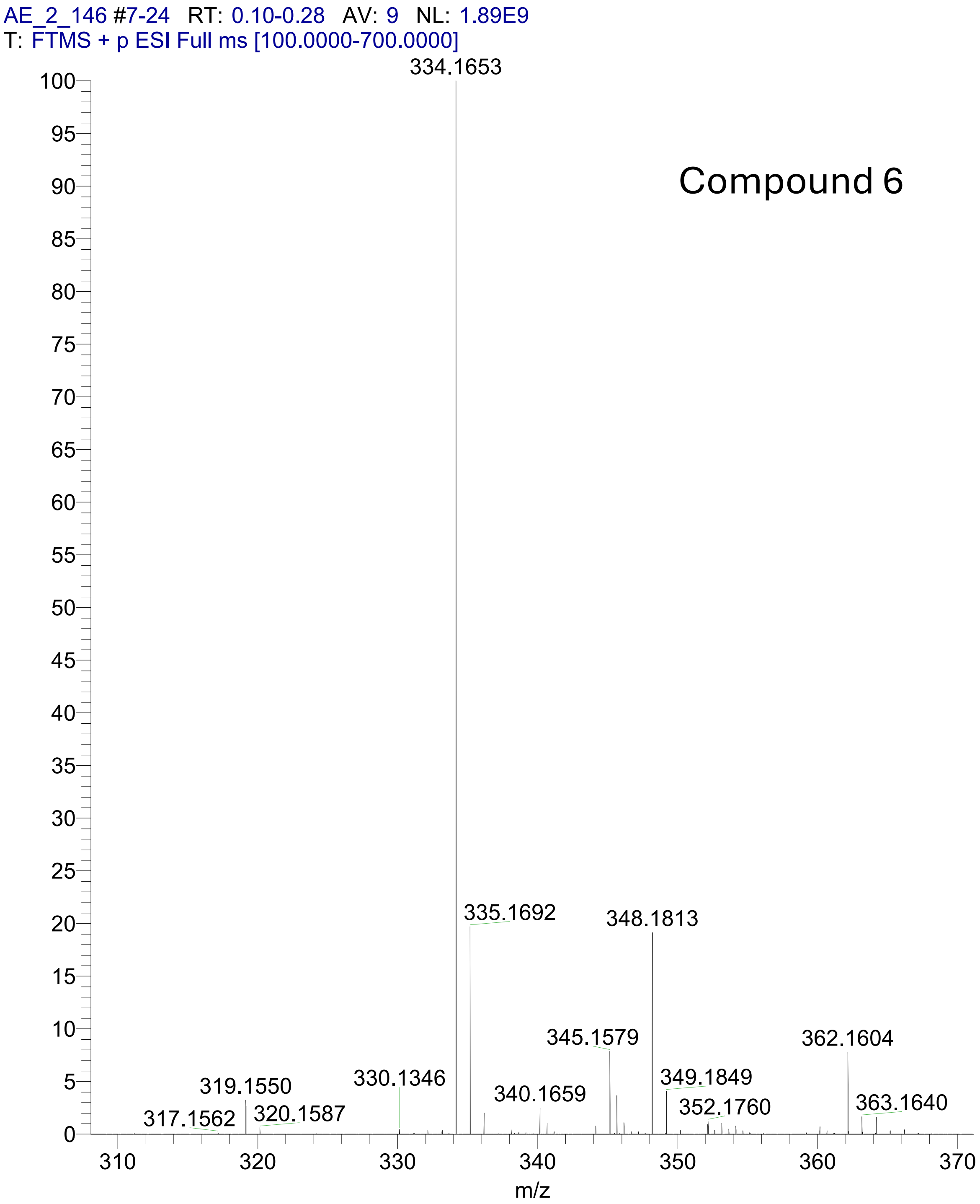

AE_2_146 #7-24 RT: 0.10-0.28 AV: 9 NL: 1.89E9
T: FTMS + p ESI Full ms [100.0000-700.0000]
Compound 6
334.1653
335.1692
348.1813
345.1579
362.1604
349.1849
340.1659
330.1346
319.1550
352.1760
363.1640
317.1562
320.1587
m/z

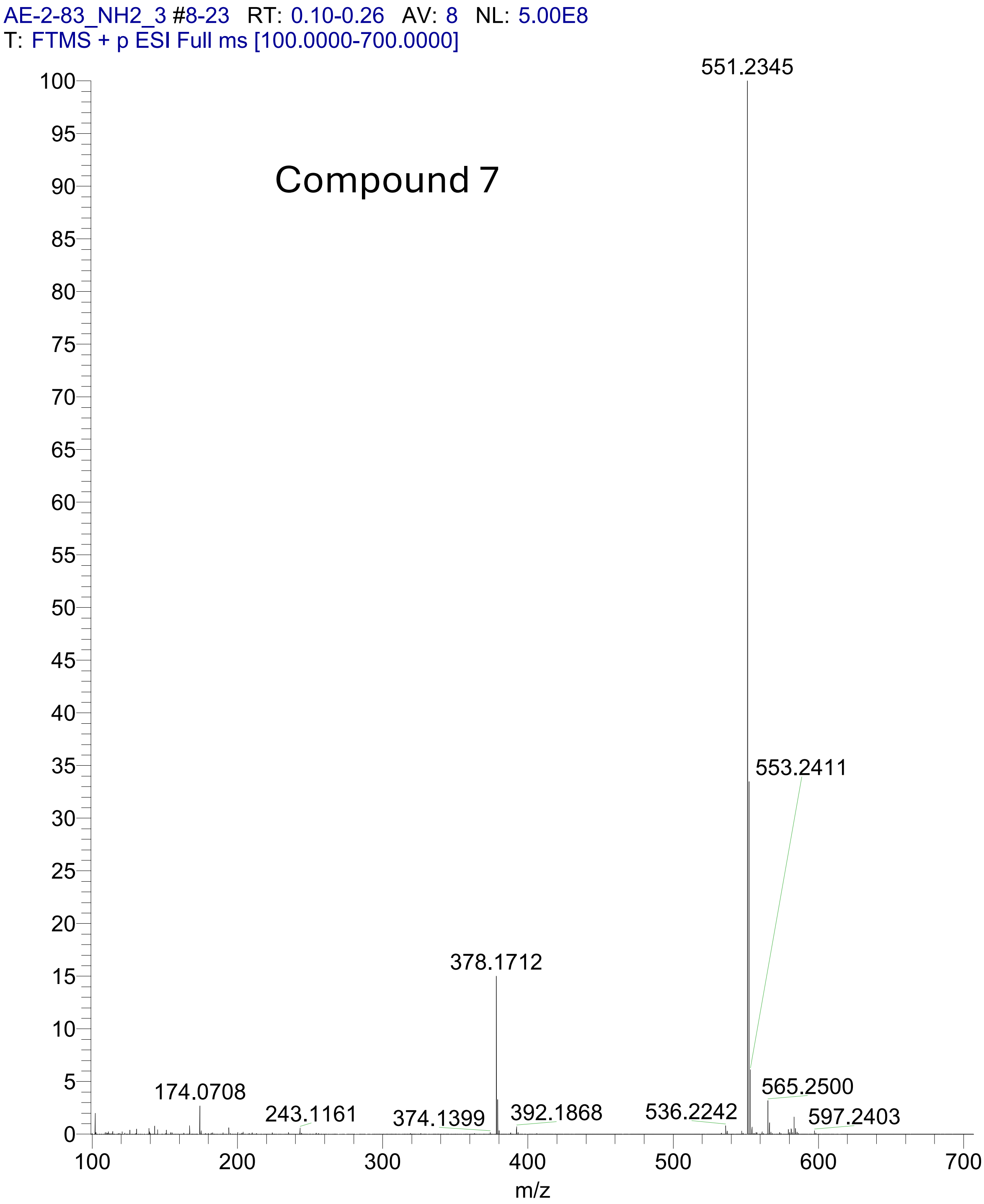
AE-2-83_NH2_3 #8-23 RT: 0.10-0.26 AV: 8 NL: 5.00E8
T: FTMS + p ESI Full ms [100.0000-700.0000]
Compound 7
551.2345
553.2411
378.1712
174.0708
243.1161
374.1399
392.1868
536.2242
565.2500
597.2403
m/z

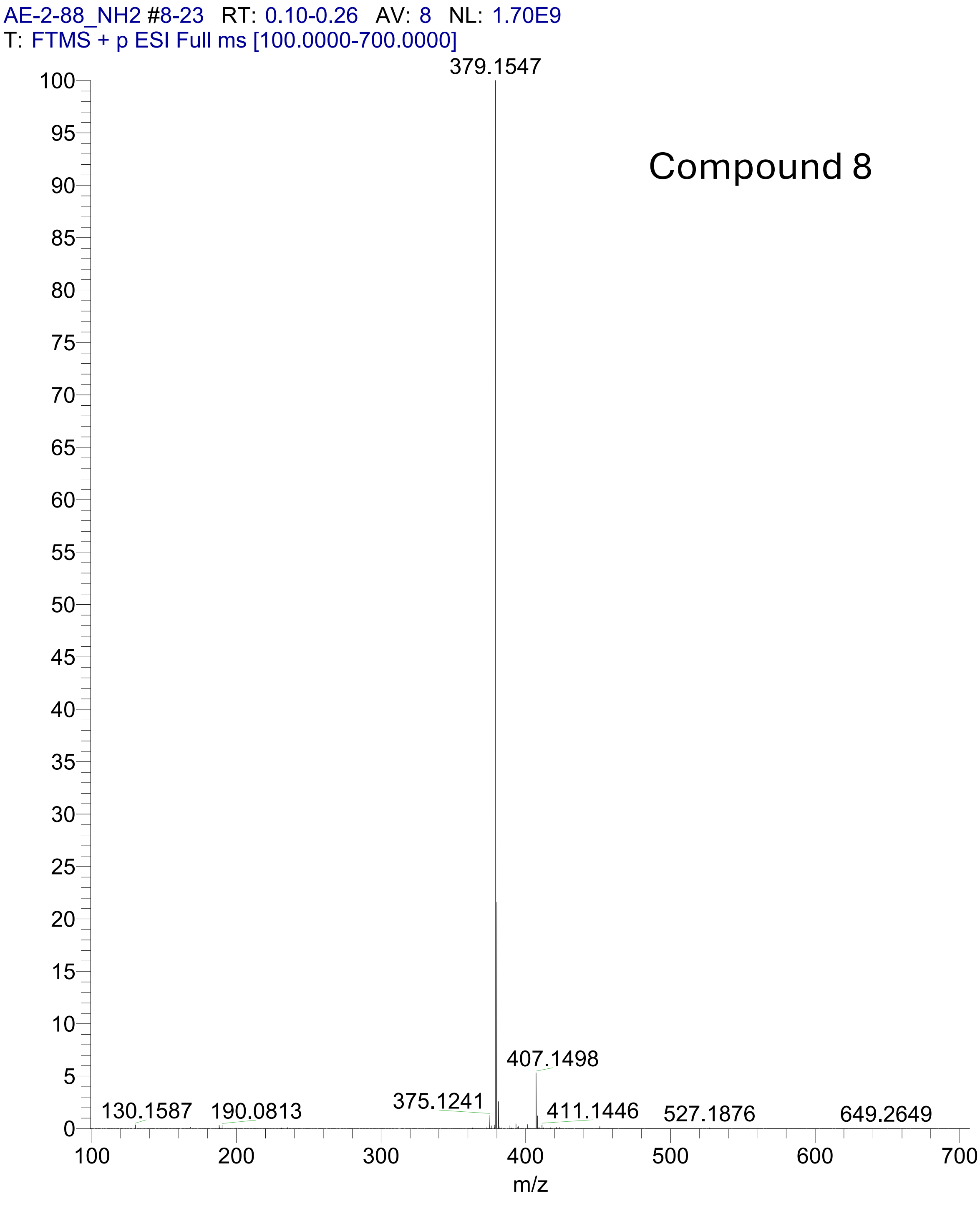
AE-2-88_NH2 #8-23 RT: 0.10-0.26 AV: 8 NL: 1.70E9
T: FTMS + p ESI Full ms [100.0000-700.0000]
379.1547
Compound 8
407.1498
375.1241
130.1587
190.0813
411.1446
527.1876
649.2649
m/z

^{1}H NMR (400 MHz, $CDCl_3$) δ 7.55 (s, 1H), 6.36 (s, 1H), 4.12 (s, 2H), 3.88 – 3.85 (m, 4H), 3.21 (s, 2H), 3.02 – 2.97 (m, 4H).

Int 1

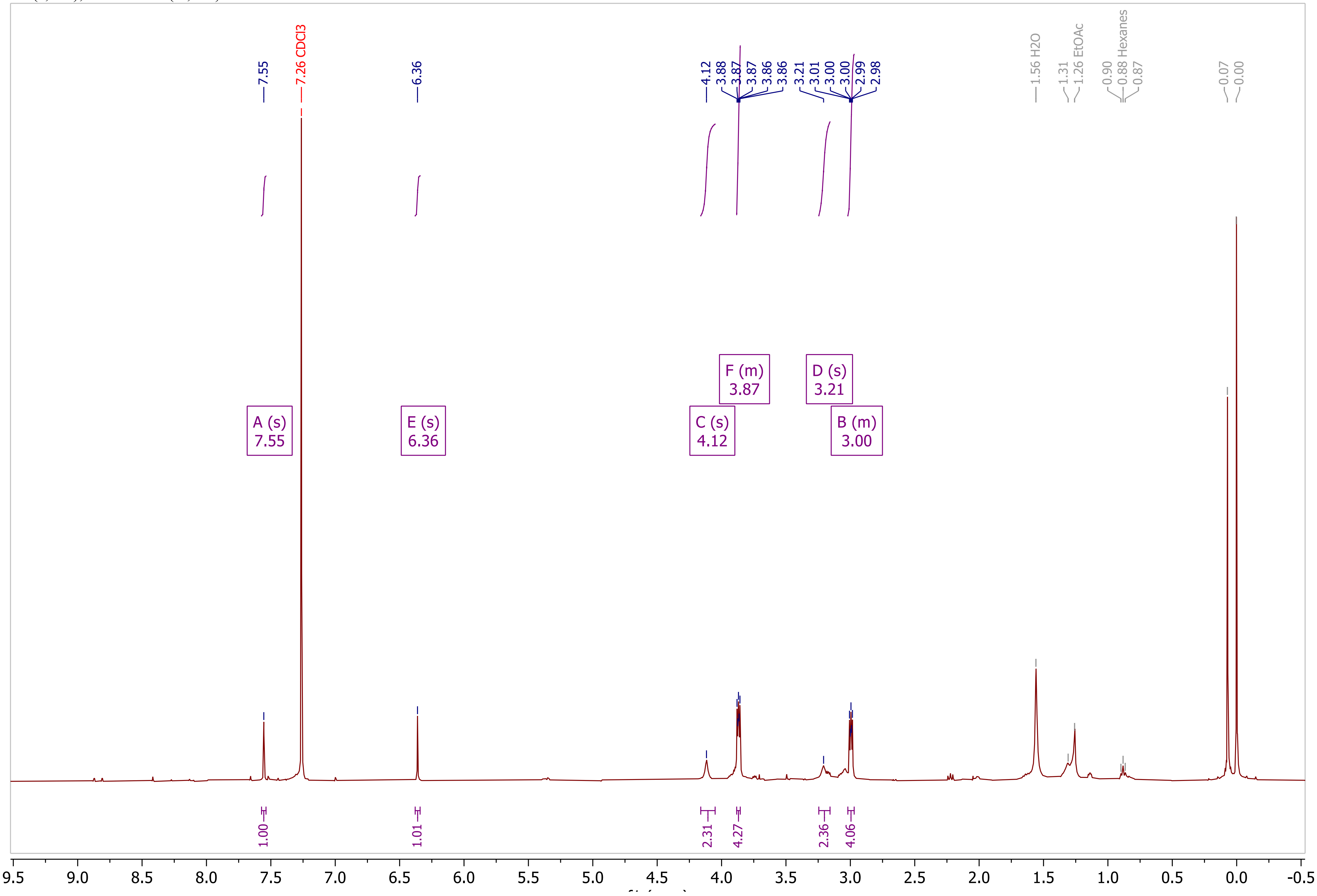

^{13}C NMR (101 MHz, $CDCl_3$) δ 143.62, 135.36, 125.18, 116.48, 105.87, 67.26, 53.07, 32.35, 26.54.

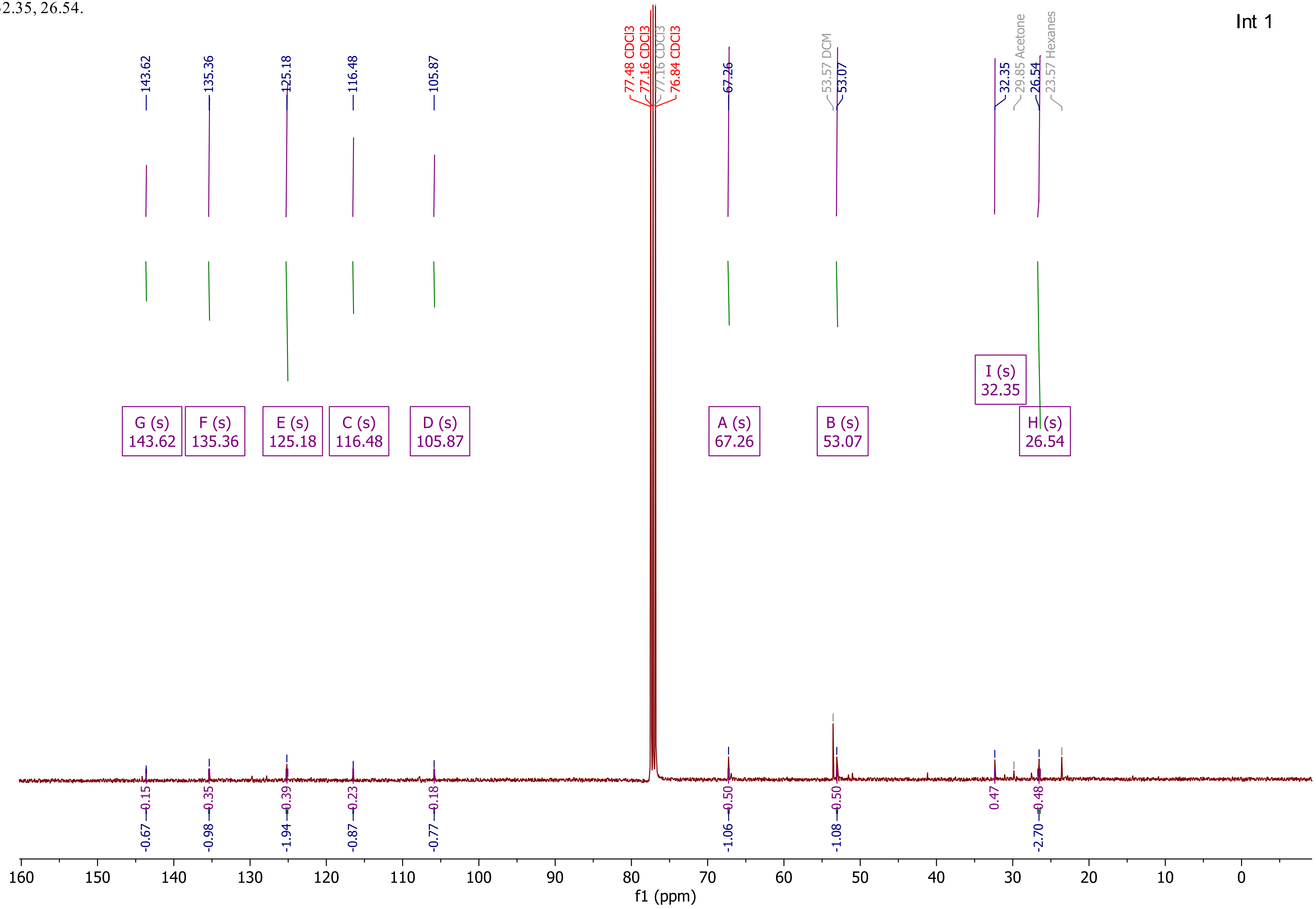

^{1}H NMR (400 MHz, $CDCl_3$) δ 8.26 (s, 1H), 7.98 (s, 1H), 7.63 (s, 1H), 7.45 (t, J = 7.9 Hz, 1H), 7.08 (d, J = 8.2 Hz, 1H), 3.92 (s, 3H), 3.90 – 3.84 (m, 4H), 3.11 – 3.00 (m, 4H).

Int 2a

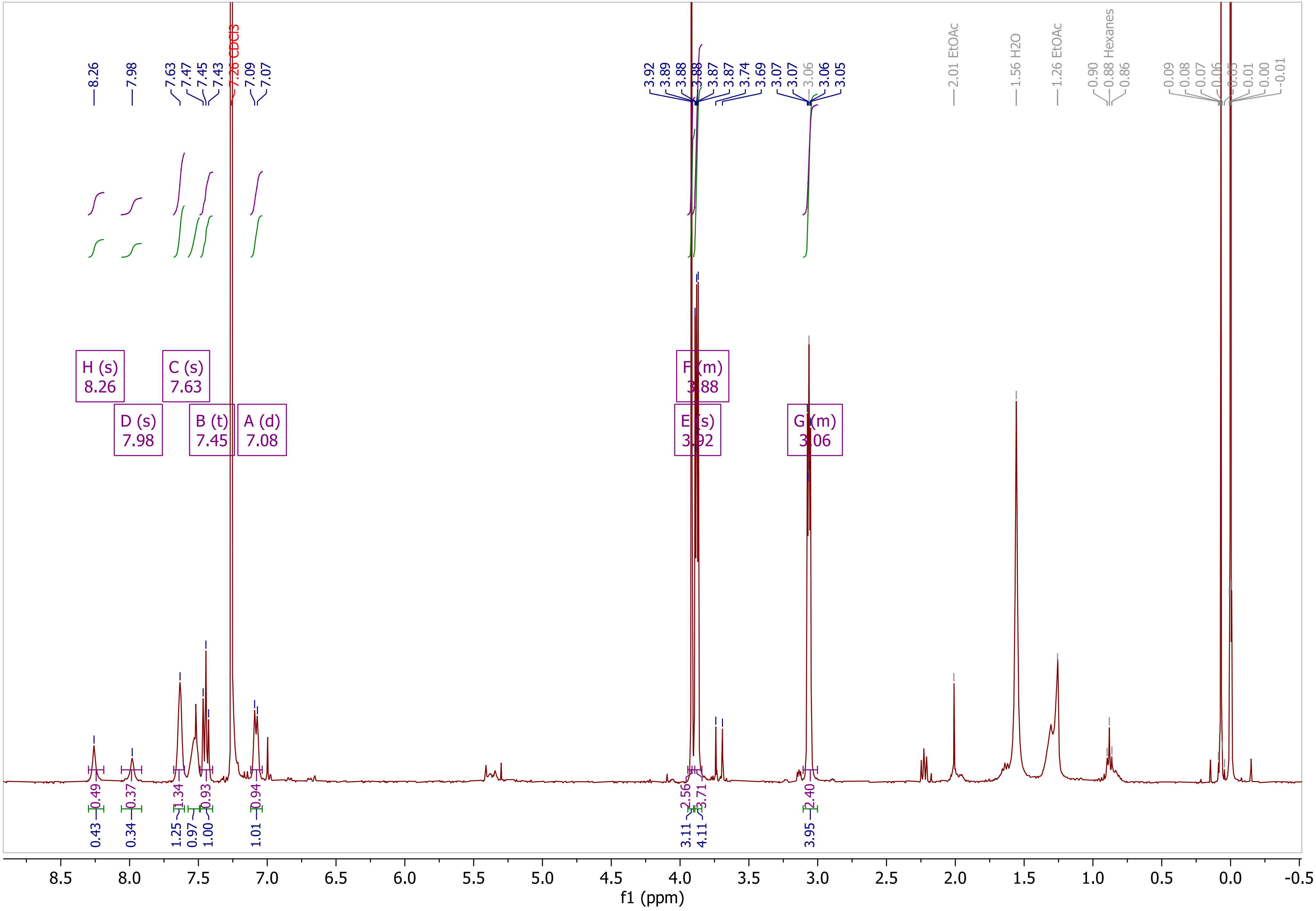

^{1}H NMR (400 MHz, $CDCl_3$) δ 8.22 (s, 1H), 7.95 (d, *J* = 11.0 Hz, 2H), 7.59 (s, 1H), 7.04 (d, *J* = 8.5 Hz, 2H), 4.13 (q, *J* = 7.0 Hz, 2H), 3.94 – 3.82 (m, 4H), 3.13 – 2.99 (m, 4H), 1.47 (t, *J* = 7.0 Hz, 3H).

Int 2b

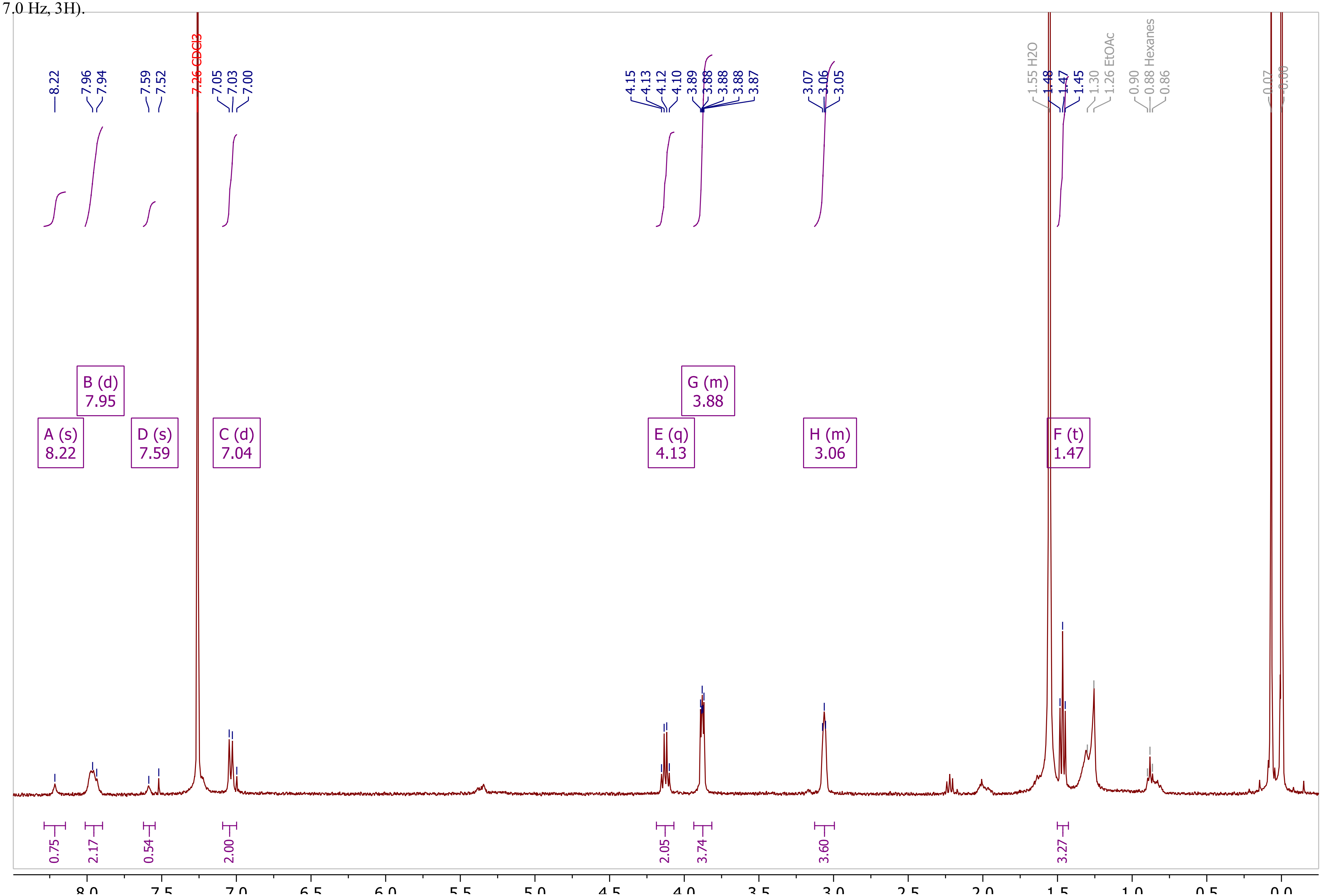

^{1}H NMR (400 MHz, $CDCl_3$) δ 10.31 – 10.26 (m, 1H), 7.82 (d, *J* = 8.7 Hz, 1H), 6.55 (dd, *J* = 8.7, 2.2 Hz, 1H), 6.45 (d, *J* = 2.3 Hz, 1H), 4.11 (dd, *J* = 12.0, 6.4 Hz, 1H), 3.93 – 3.64 (m, 10H), 3.10 – 3.01 (m, 1H).

Int 2c

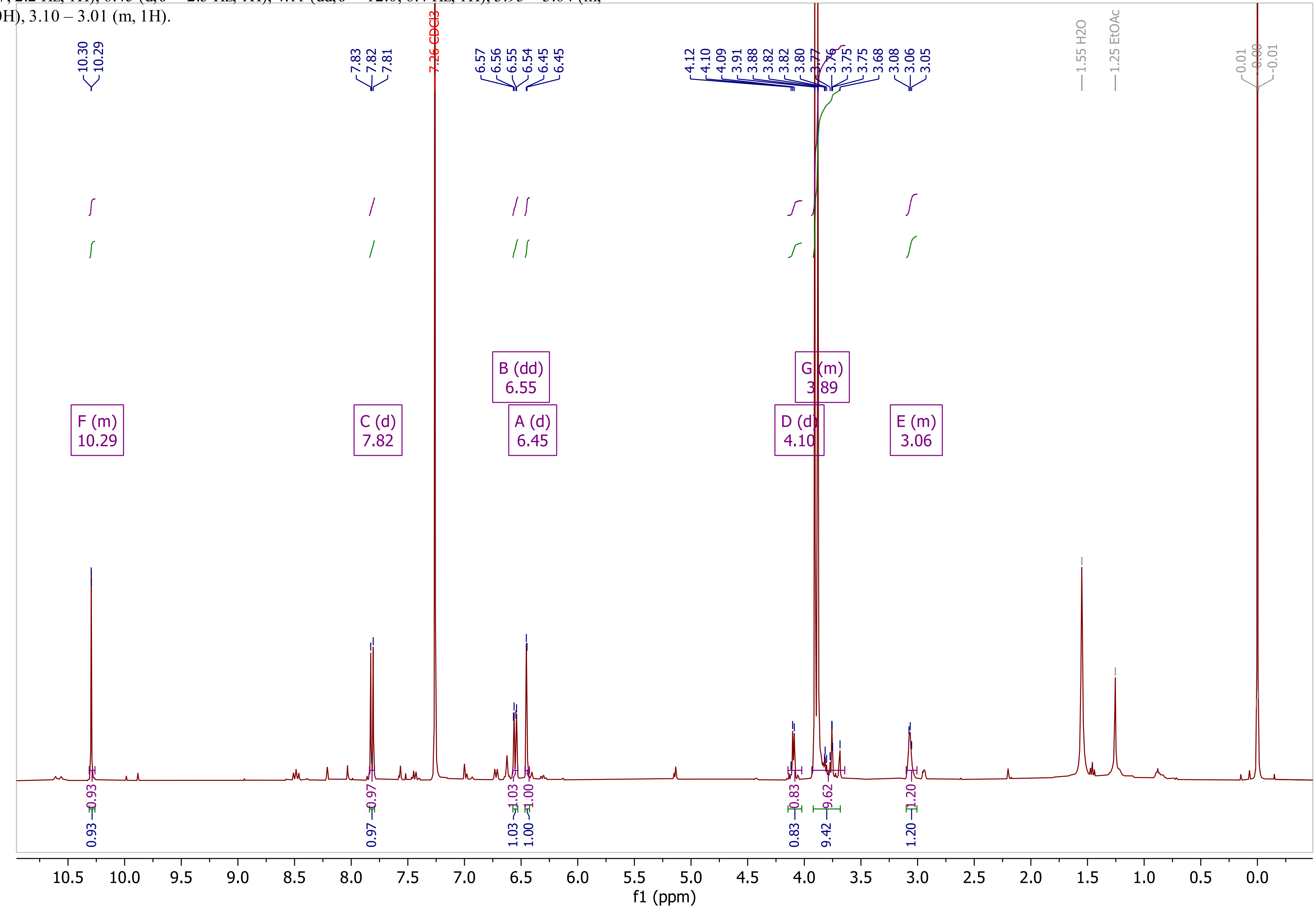

H NMR (400 MHz, $CDCl_3$) δ 8.55 (s, 1H), 8.00 (d, J = 6.2 Hz, 1H), 7.87 (d, J = 8.5 Hz, H), 7.71 (s, 1H), 7.64 (d, J = 8.2 Hz, 1H), 7.52 (s, 1H), 7.00 (s, 1H), 6.30 (s, 1H), 4.97 (s, H), 3.96 – 3.81 (m, 4H), 3.16 – 2.97 (m, 4H), 2.35 (t, J = 6.8 Hz, 1H), 2.24 (d, J = 6.9 Hz, H).

Int 2d

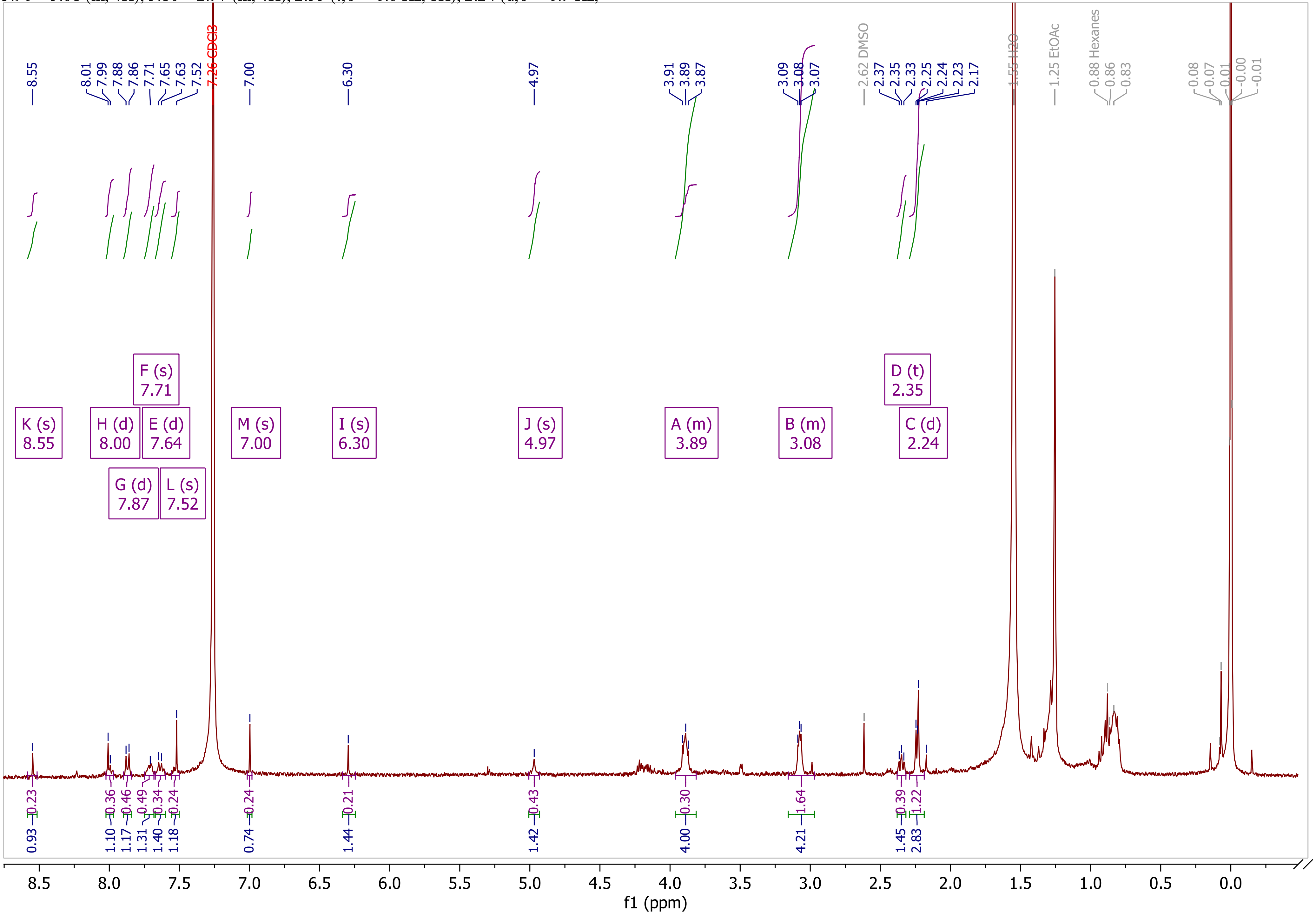

^{1}H NMR (400 MHz, $CDCl_3$) δ 8.23 (s, 1H), 7.99 – 7.90 (m, 2H), 7.61 (s, 1H), 7.37 (d, *J* = 7.9 Hz, 2H), 3.93 – 3.82 (m, 4H), 3.07 (d, *J* = 5.4 Hz, 4H), 2.56 (s, 3H).

Int 2e

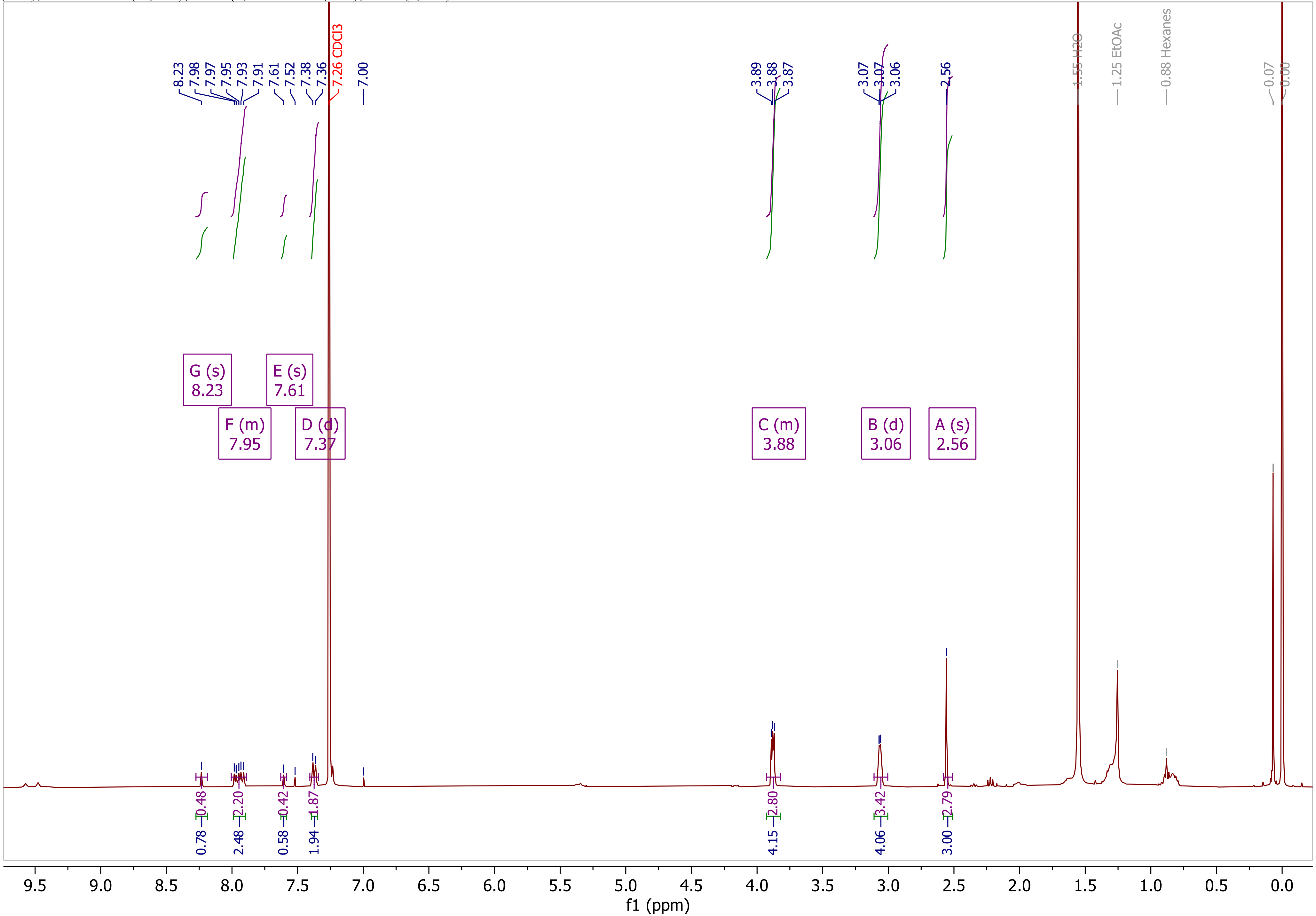

^{1}H NMR (400 MHz, $CDCl_3$) δ 8.79 (s, 1H), 8.01 (d, *J* = 2.4 Hz, 1H), 7.85 (d, *J* = 3.1 Hz, 1H), 7.71 (d, *J* = 2.9 Hz, 1H), 7.55 (s, 1H), 7.51 (d, *J* = 3.3 Hz, 1H), 7.45 (dd, *J* = 8.1, 6.1 Hz, 1H), 7.38 – 7.32 (m, 2H), 6.35 (d, *J* = 9.5 Hz, 1H), 3.93 – 3.85 (m, 6H), 3.09 (t, *J* = 4.7 Hz, 4H), 3.01 – 2.97 (m, 2H).

Int 2f

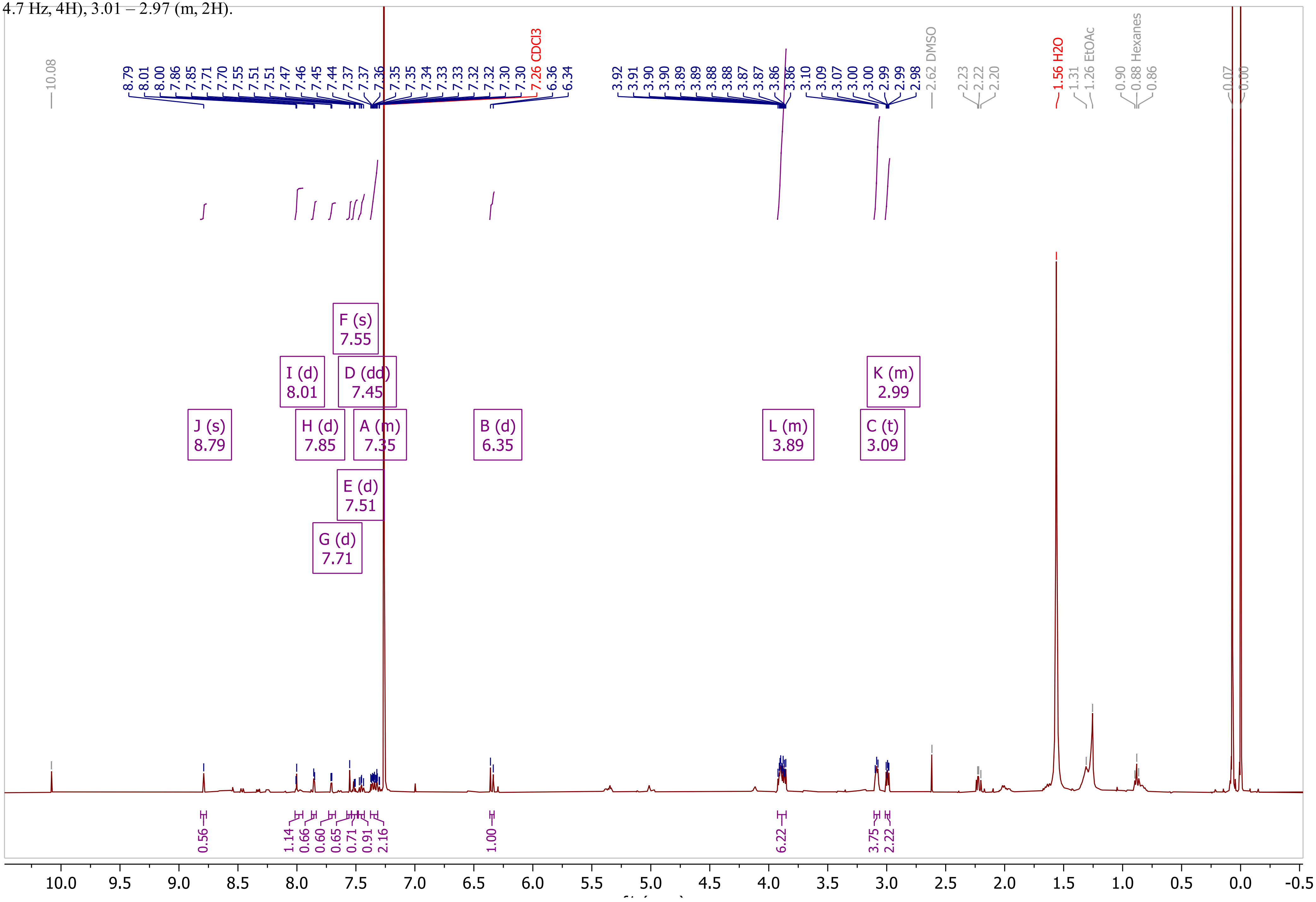

^{1}H NMR (400 MHz, $CDCl_3$) δ 7.93 (s, 1H), 7.65 (s, 1H), 7.59 – 7.50 (m, 2H), 7.17 – 7.08 (m, 4H), 6.71 (s, 1H), 6.52 (s, 1H), 5.50 (d, *J* = 5.5 Hz, 2H), 3.88 (d, *J* = 4.2 Hz, 4H), 3.11 – 2.99 (m, 4H).

Int 2g

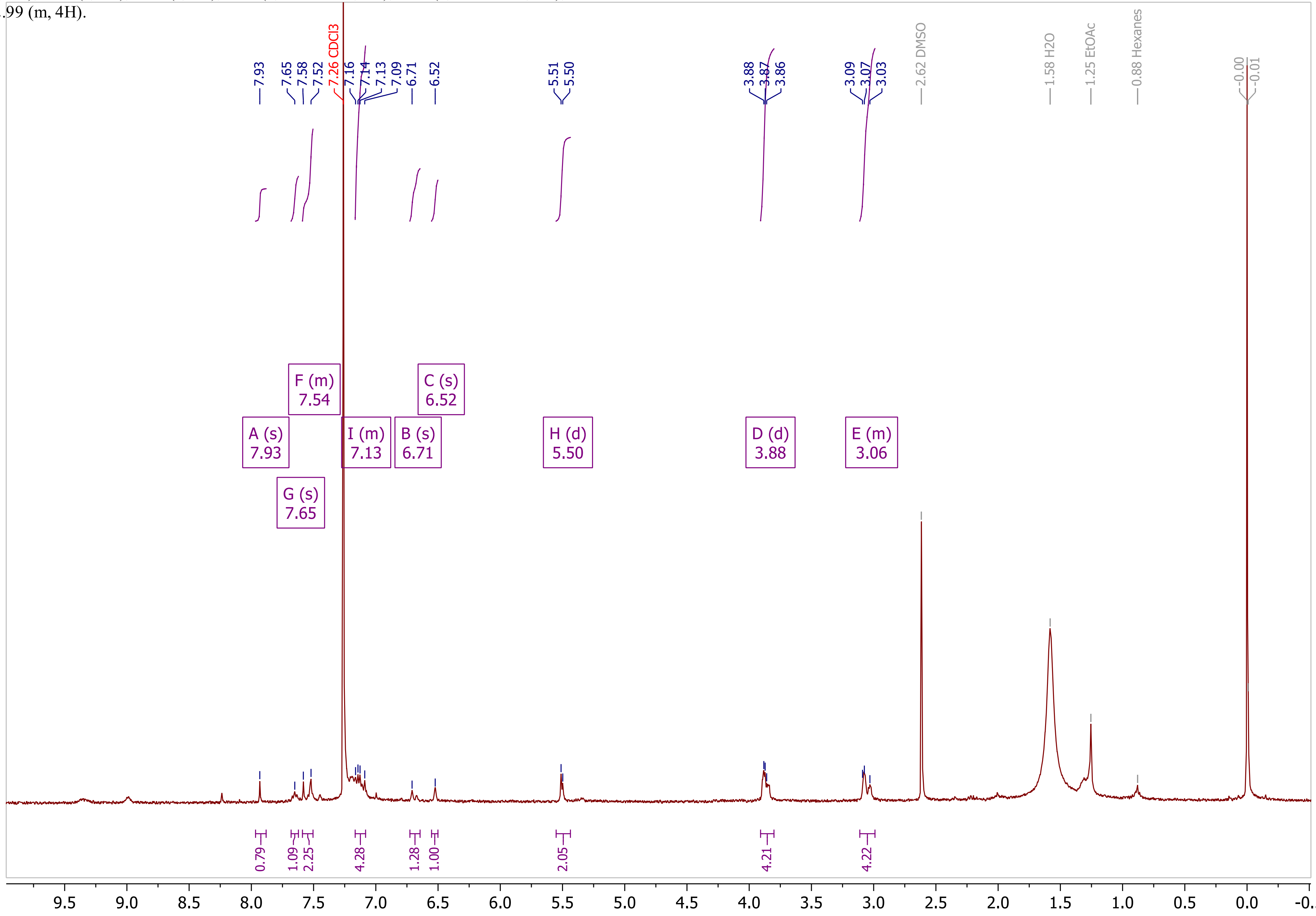

^{1}H NMR (400 MHz, $CDCl_3$) δ 8.22 (s, 1H), 8.01 (s, 1H), 7.97 – 7.89 (m, 1H), 7.42 (d, *J* = 3.7 Hz, 1H), 7.37 (d, *J* = 3.6 Hz, 1H), 7.22 – 7.16 (m, 1H), 7.08 – 7.04 (m, 1H), 3.93 – 3.84 (m, 4H), 3.07 (t, *J* = 4.2 Hz, 4H).

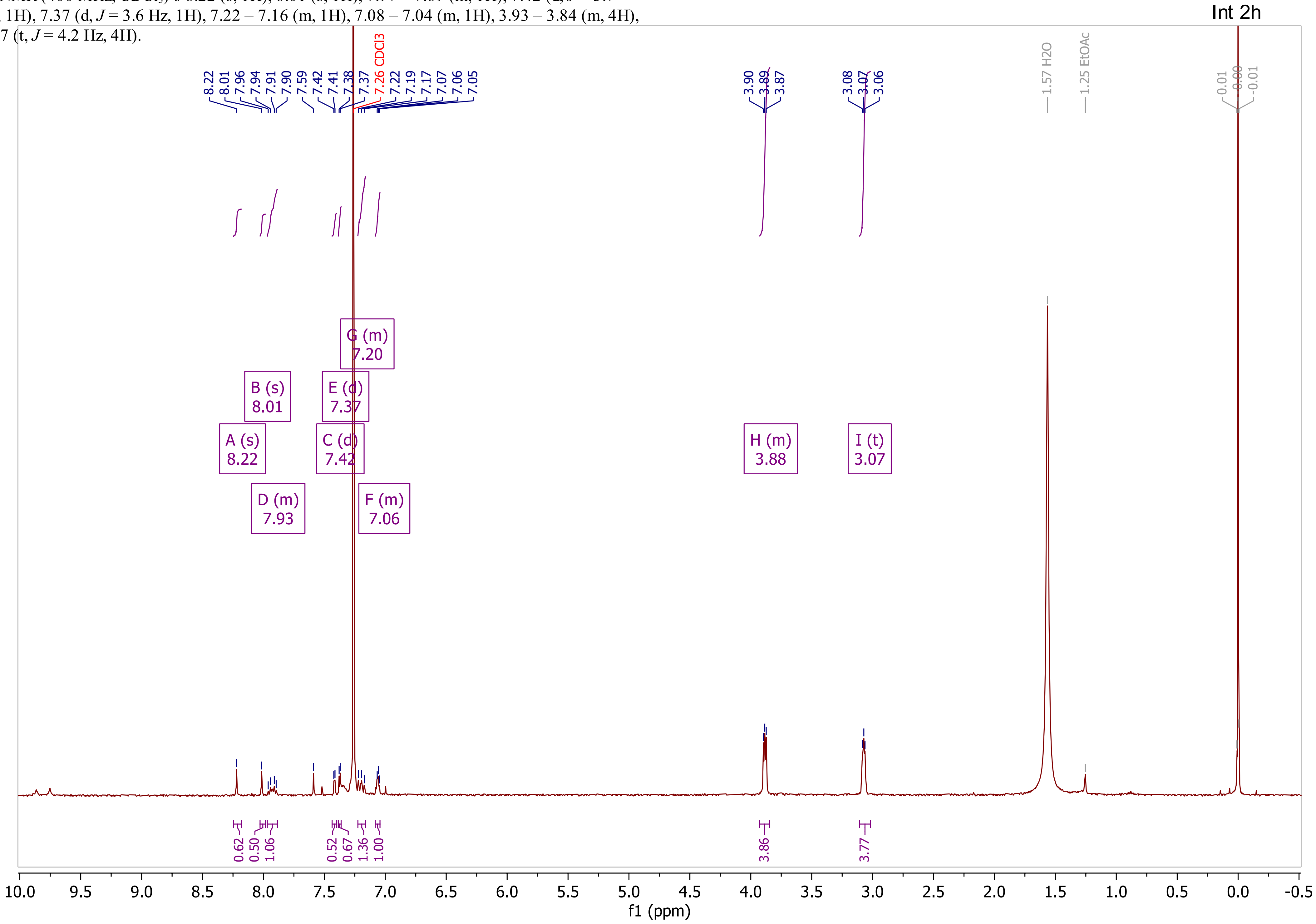

^{1}H NMR (400 MHz, $CDCl_3$) δ 8.57 (s, 1H), 7.57 (dt, J = 8.7, 2.2 Hz, 1H), 7.47 (t, J = 7.2 Hz, 1H), 7.29 (s, 1H), 7.08 (d, J = 6.9 Hz, 1H), 6.90 – 6.80 (m, 2H), 3.92 – 3.85 (m, 8H), 3.15 (s, 2H), 2.95 (t, J = 4.6 Hz, 4H), 2.86 (s, 3H).

Compound 1

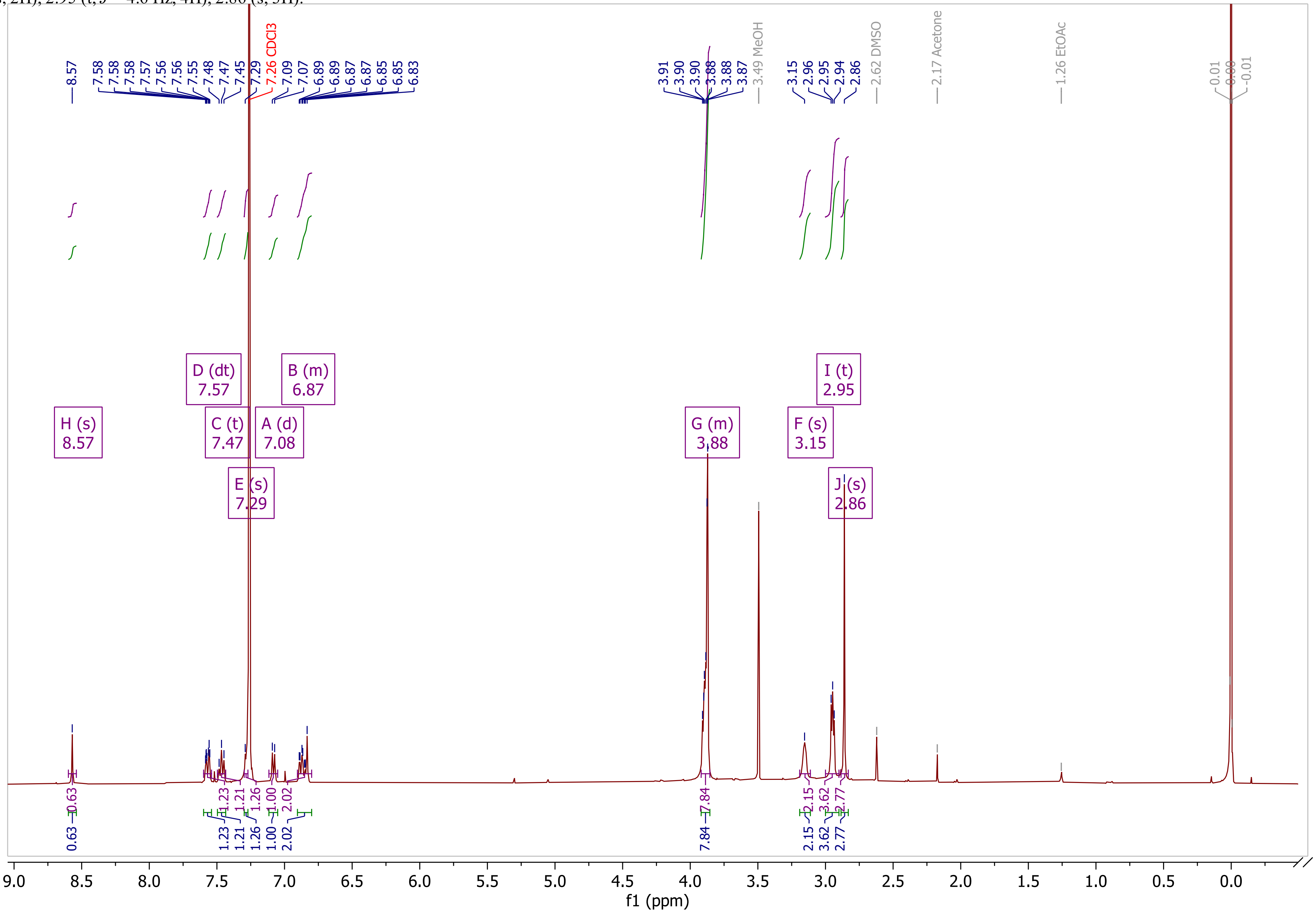

^{13}C NMR (101 MHz, $CDCl_3$) δ 160.04, 129.99, 118.24, 116.81, 110.76, 67.71, 55.46, 52.25, 50.81, 42.77.

Compound 1

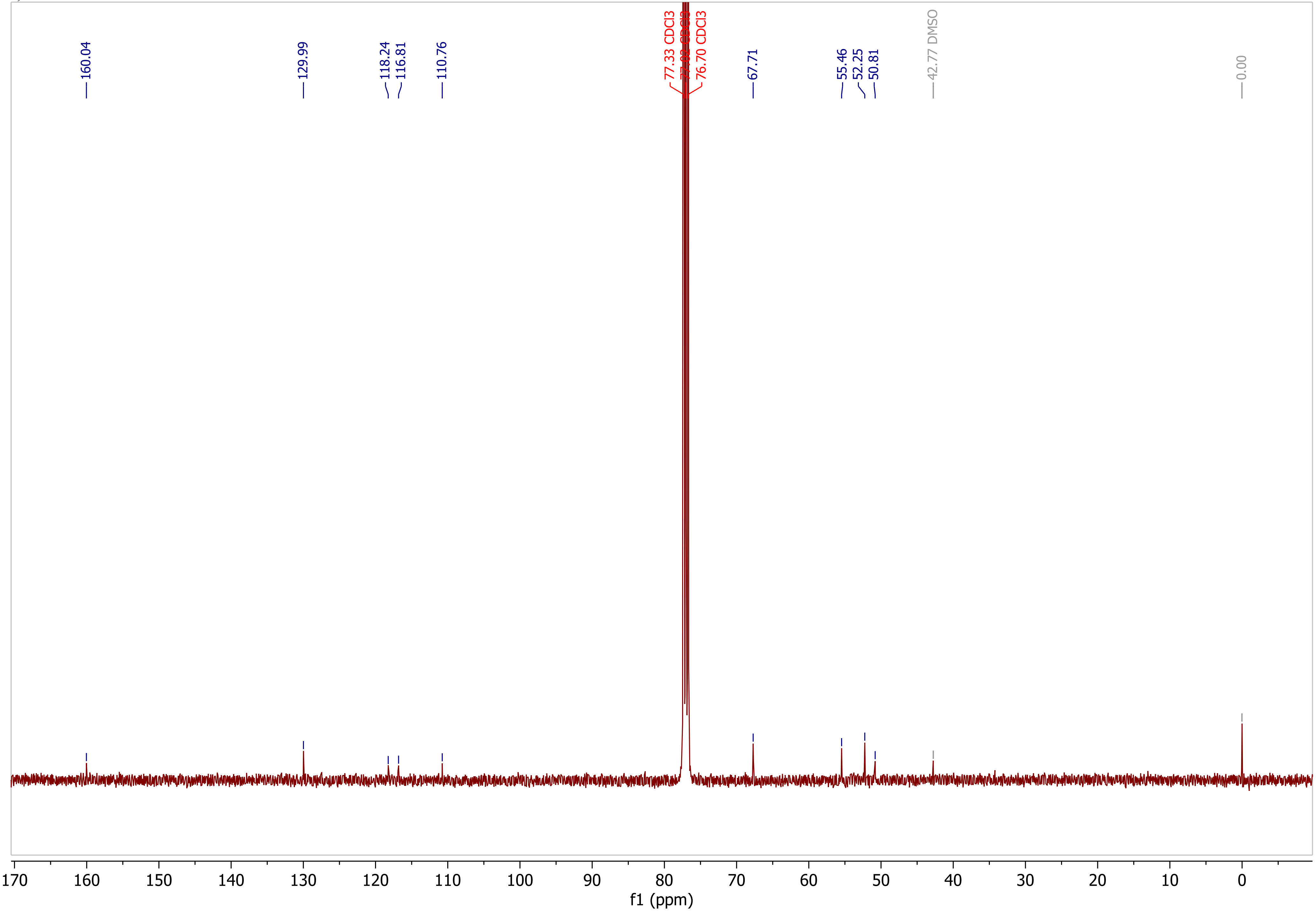

^{1}H NMR (400 MHz, $CDCl_3$) δ 8.85 (s, 1H), 7.88 (t, *J* = 9.0 Hz, 2H), 7.18 (s, 1H), 7.07 (d, *J* = 14.1 Hz, 1H), 6.89 – 6.77 (m, 3H), 4.00 (q, *J* = 6.9 Hz, 3H), 3.86 (s, 4H), 3.11 (s, 2H), 2.90 (t, *J* = 4.7 Hz, 4H), 2.83 (s, 3H).

Compound 2

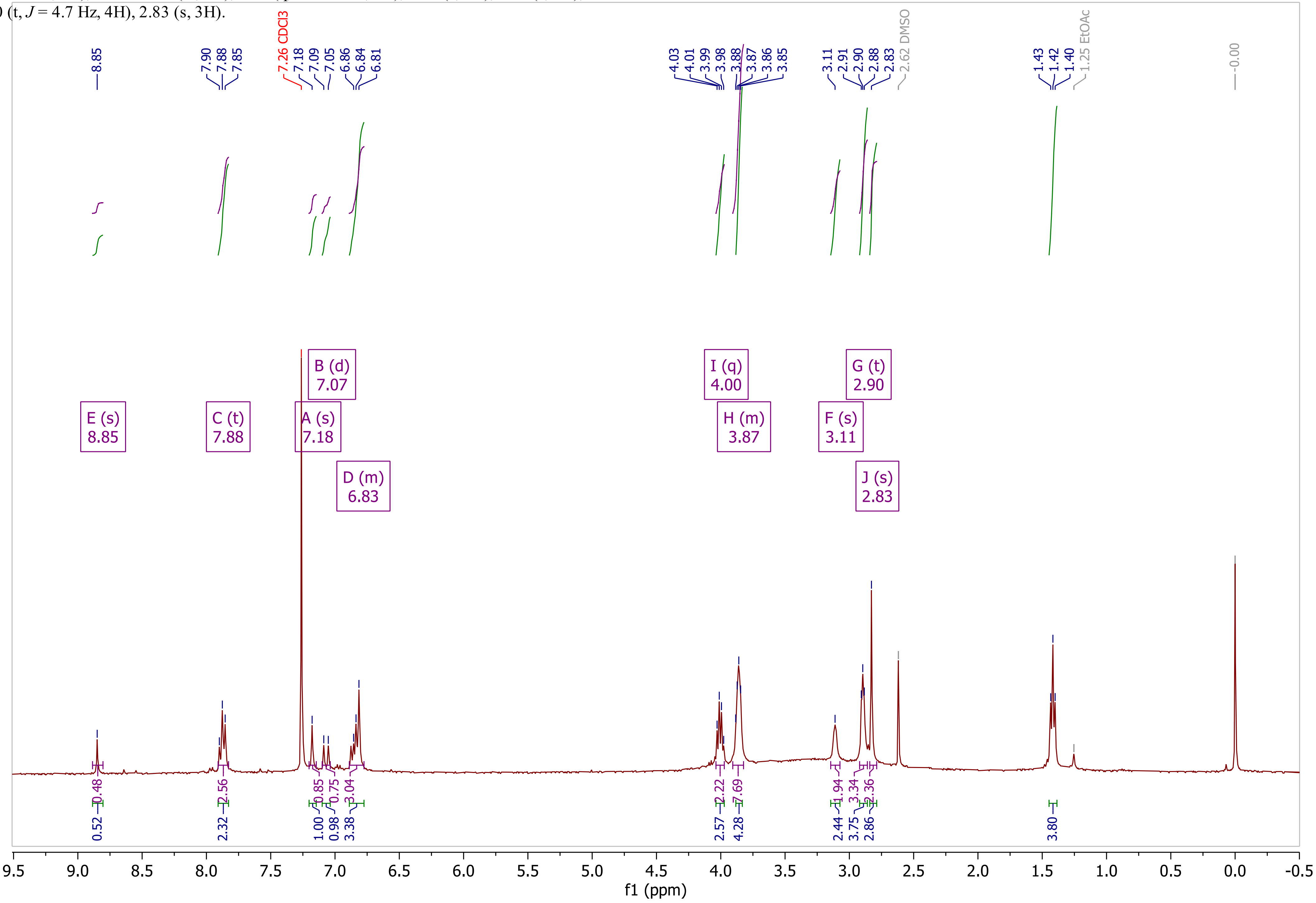

^{13}C NMR (101 MHz, $CDCl_3$) δ 168.55, 160.98, 139.87, 138.12, 128.31, 114.83, 114.81, 105.90, 67.60, 63.57, 52.02, 50.68, 42.64.

Compound 2

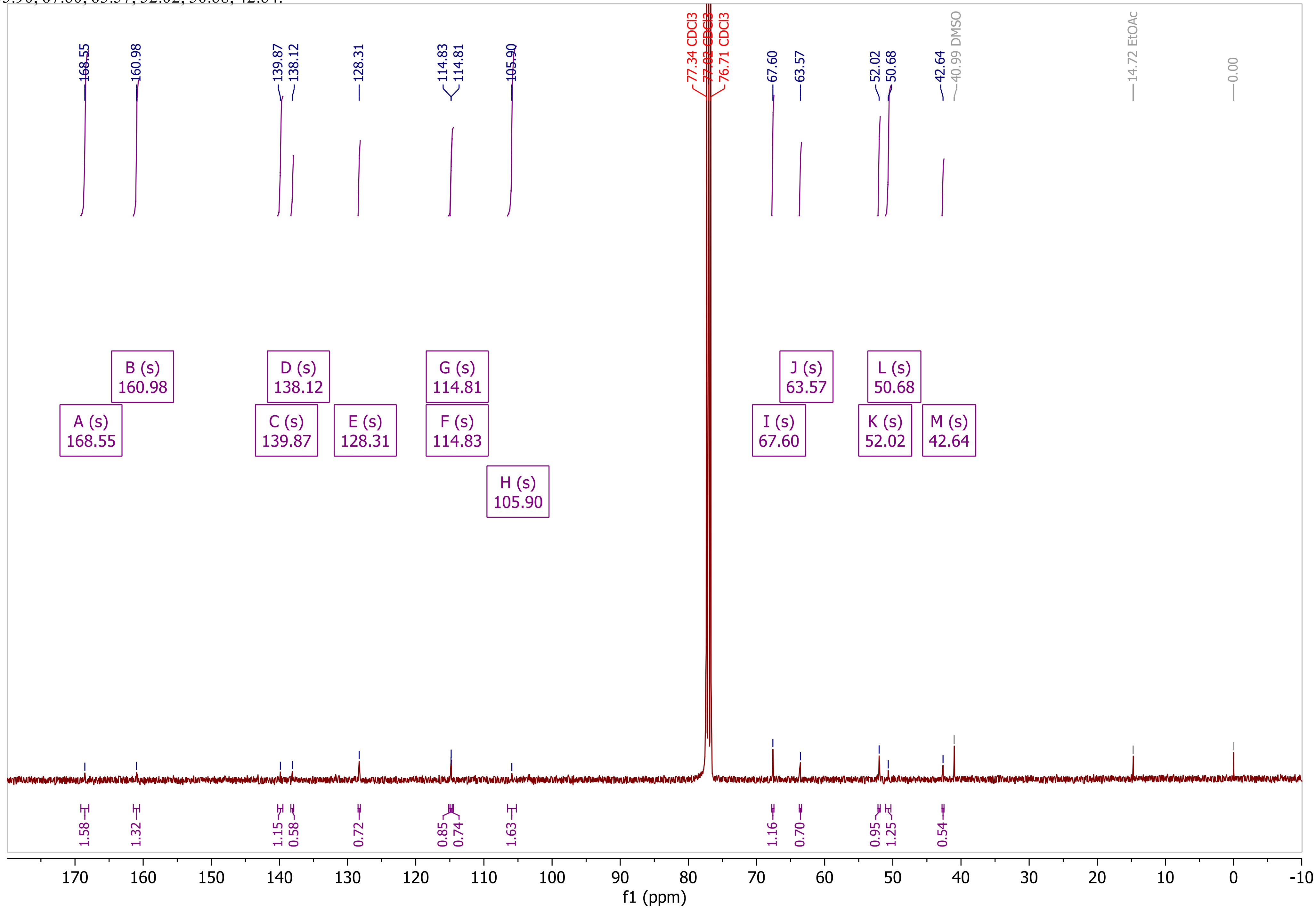

^{1}H NMR (400 MHz, $CDCl_3$) δ 8.44 (d, J = 8.8 Hz, 1H), 7.35 (s, 1H), 6.94 (s, 1H), 6.68 (dd, J = 8.8, 2.3 Hz, 1H), 6.58 (d, J = 2.3 Hz, 1H), 4.05 (s, 3H), 3.91 – 3.86 (m, 7H), 2.97 (t, J = 4.6 Hz, 4H).

Compound 3

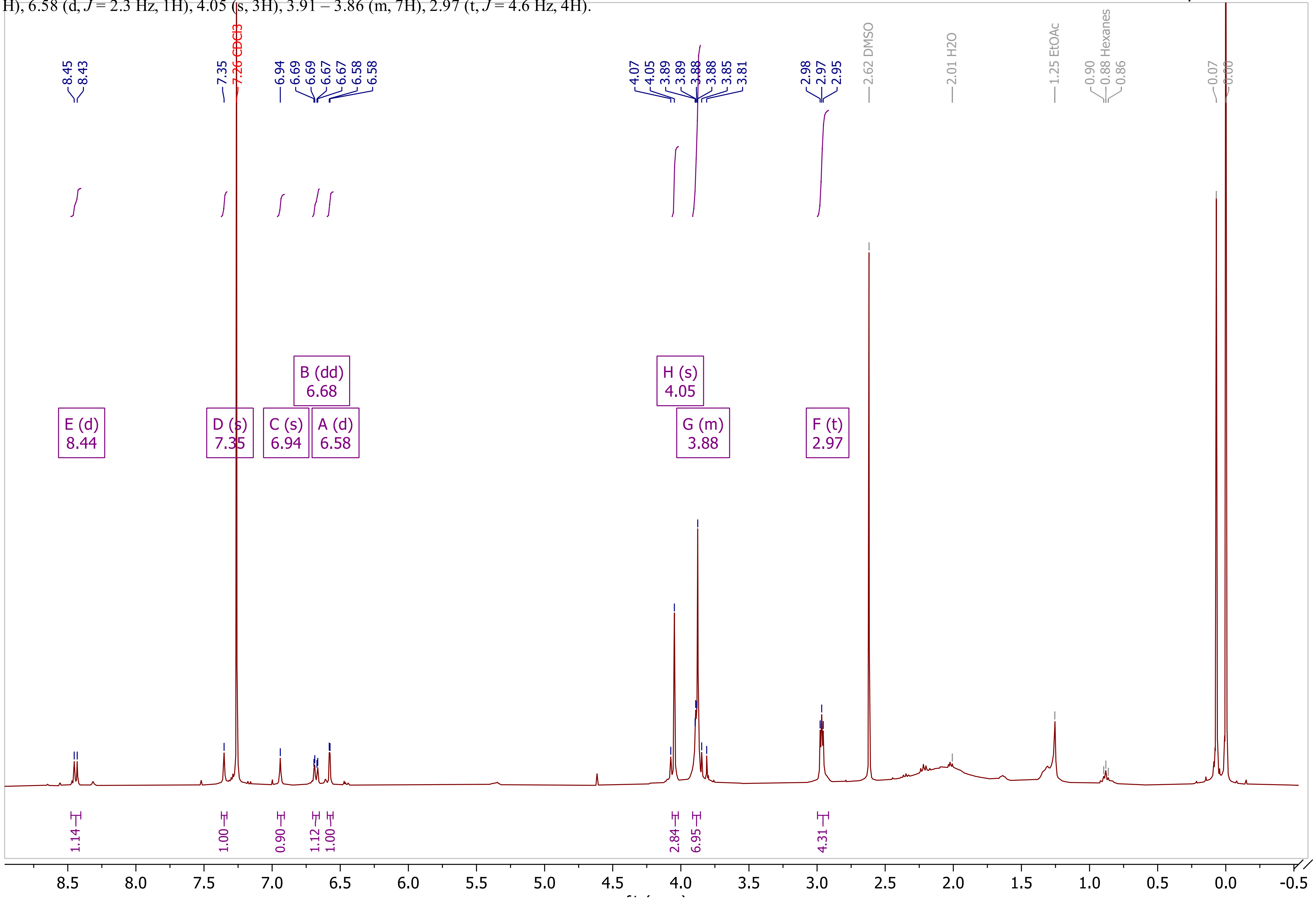

^{13}C NMR (101 MHz, $CDCl_3$) δ 131.09, 106.15, 99.14, 83.56, 67.93, 56.14, 55.74, 52.56.

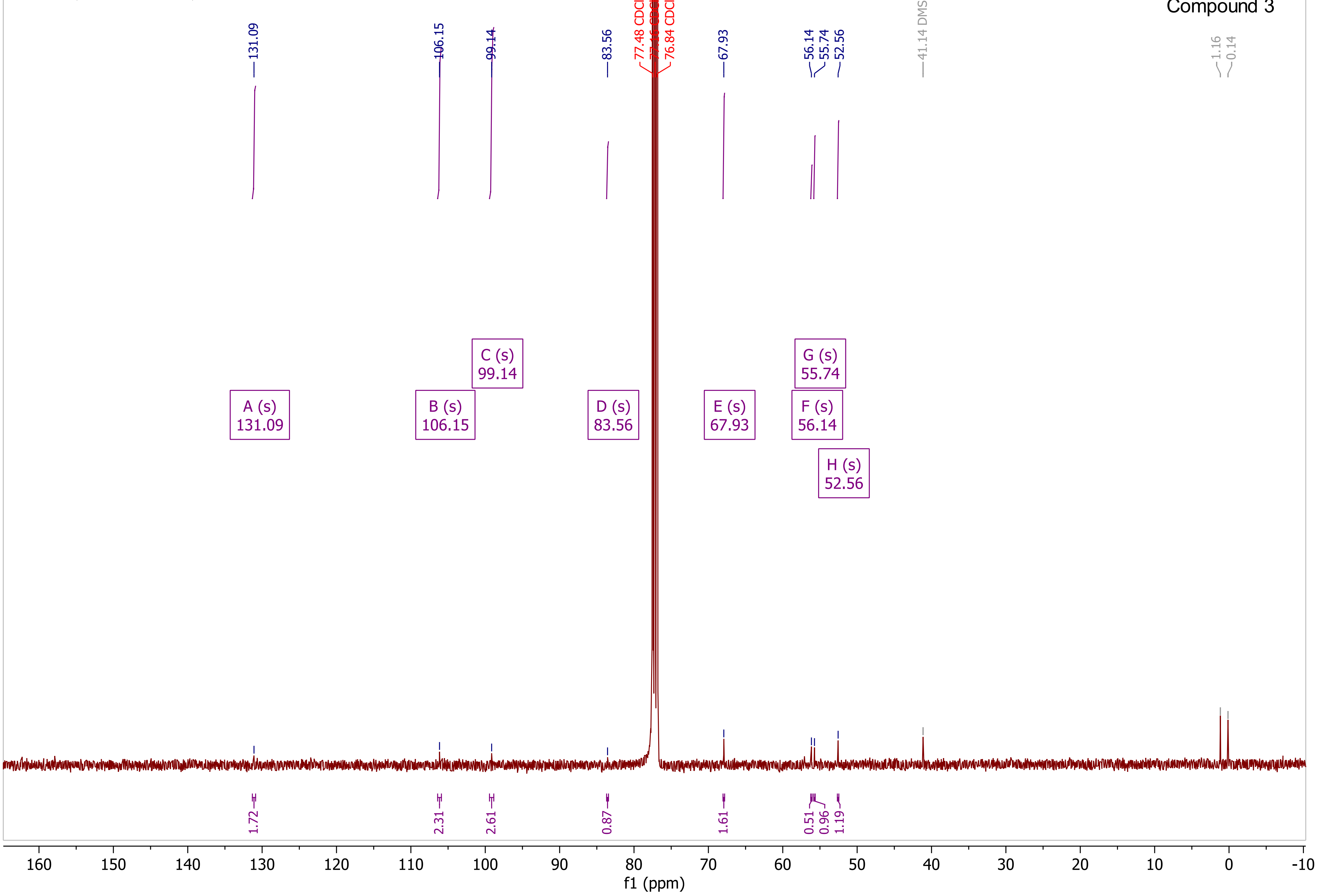

NMR (400 MHz, MeOD) δ 7.85 (d, J = 8.8 Hz, 2H), 7.66 (d, J = 8.9 Hz, 2H), 7.29 (d, J = 8.5 Hz, 1H), 7.18 (s, 1H), 7.00 (d, = 8.5 Hz, 1H), 6.90 (d, J = 8.1 Hz, 1H), 3.78 (t, J = 4.5 Hz, 7H), 2.89 – 2.77 (m, 6H), 2.18 (s, 2H), 2.07 (s, 3H).

Compound 4

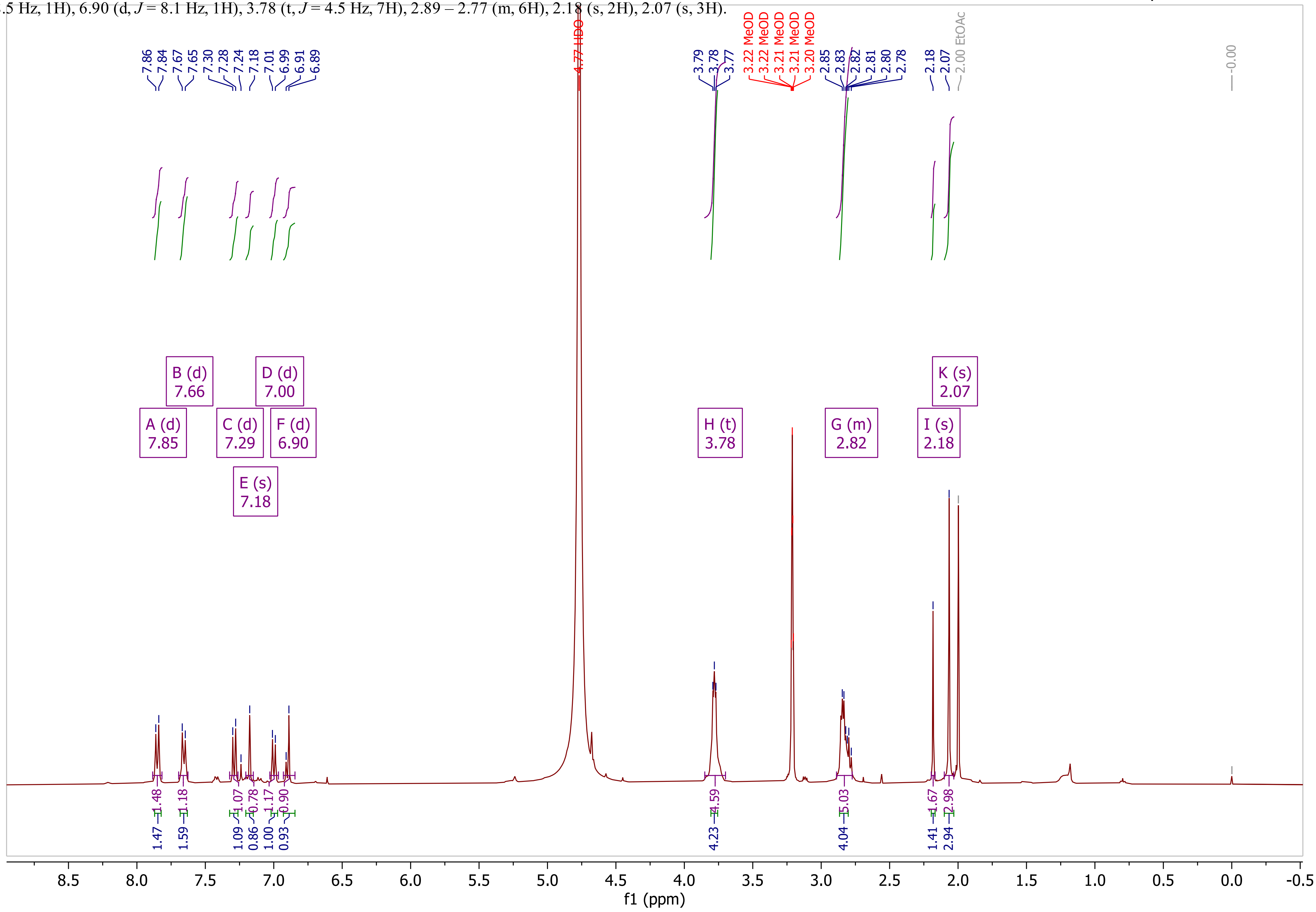

^{13}C NMR (101 MHz, MeOD) δ 149.07, 140.82, 140.21, 138.42, 135.86, 133.76, 133.49, 128.80, 126.74, 119.92, 119.71, 105.71, 97.83, 67.24, 67.20, 52.56, 52.12, 22.59, 22.30, 19.48.

Compound 4

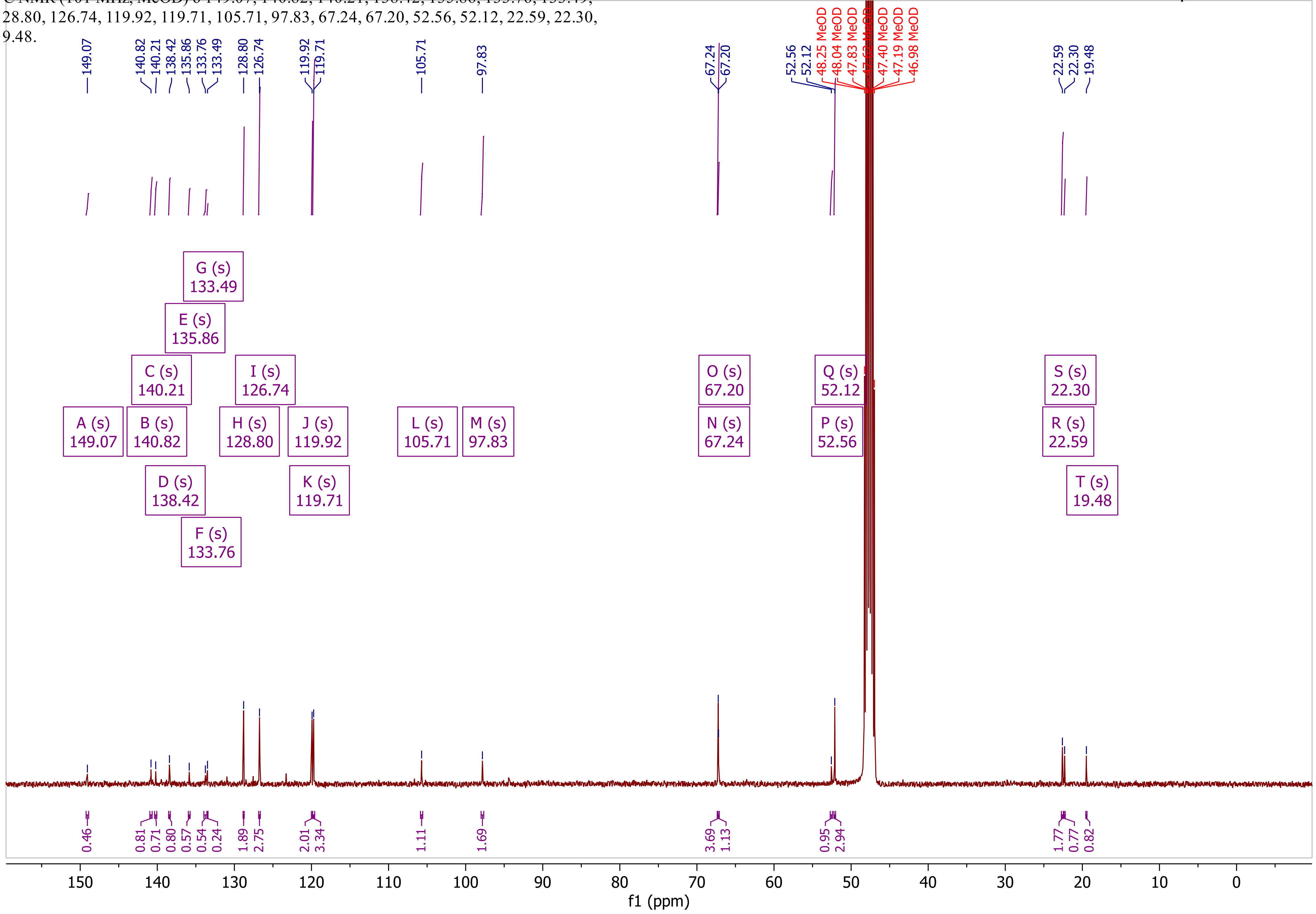

Compound 5

^{1}H NMR (400 MHz, $CDCl_3$) δ 8.74 (s, 1H), 7.75 (d, J = 8.0 Hz, 2H), 7.06 (d, J = 6.3 Hz, 3H), 6.73 (s, 1H), 3.86 (t, J = 4.4 Hz, 4H), 2.87 (t, J = 4.6 Hz, 4H), 2.45 (s, 3H).

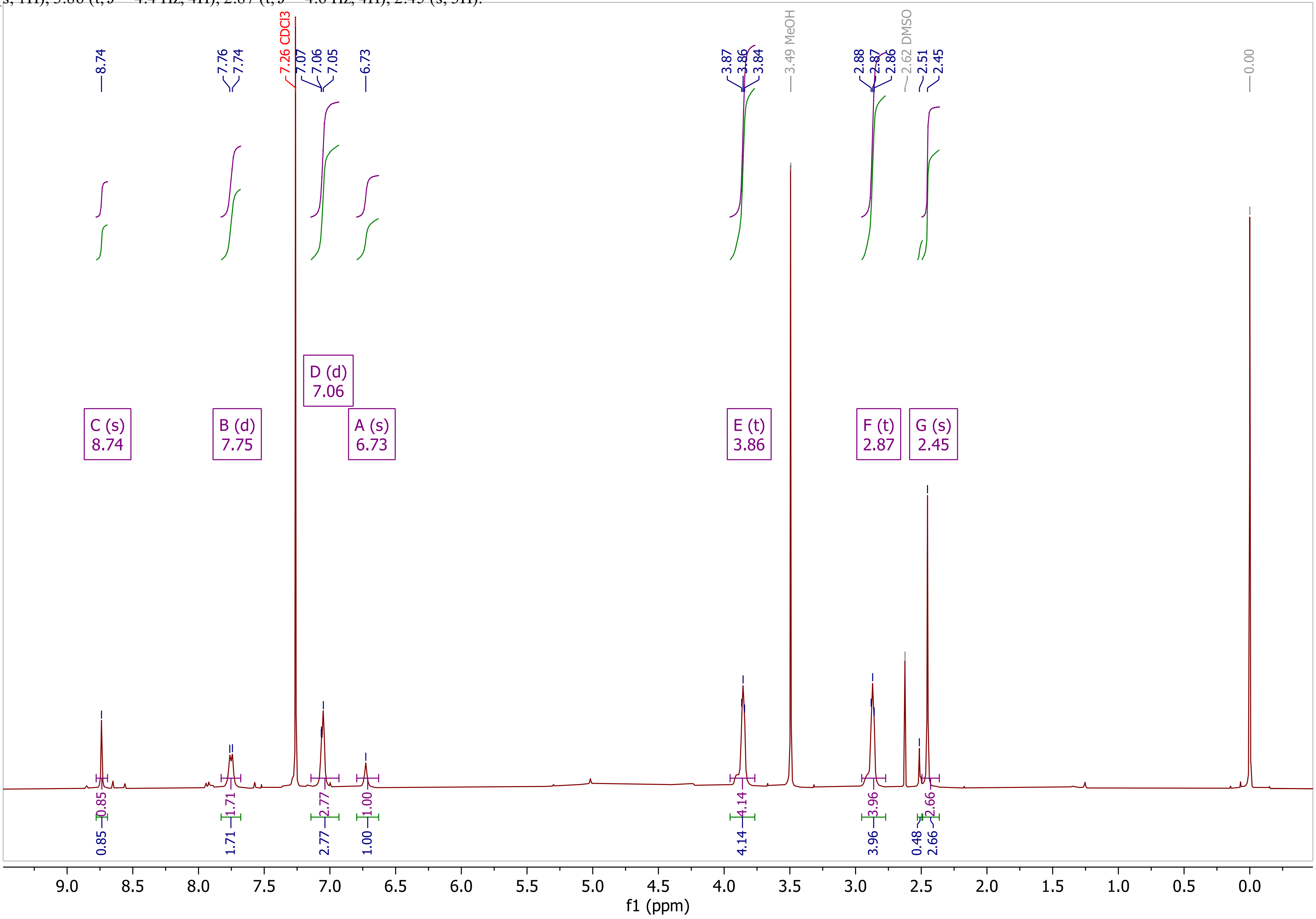

^{13}C NMR (101 MHz, $CDCl_3$) δ 168.19, 147.15, 143.04, 140.28, 138.60, 126.68, 125.55, 105.63, 97.54, 67.58, 51.96, 50.89, 14.82.

Compound 5

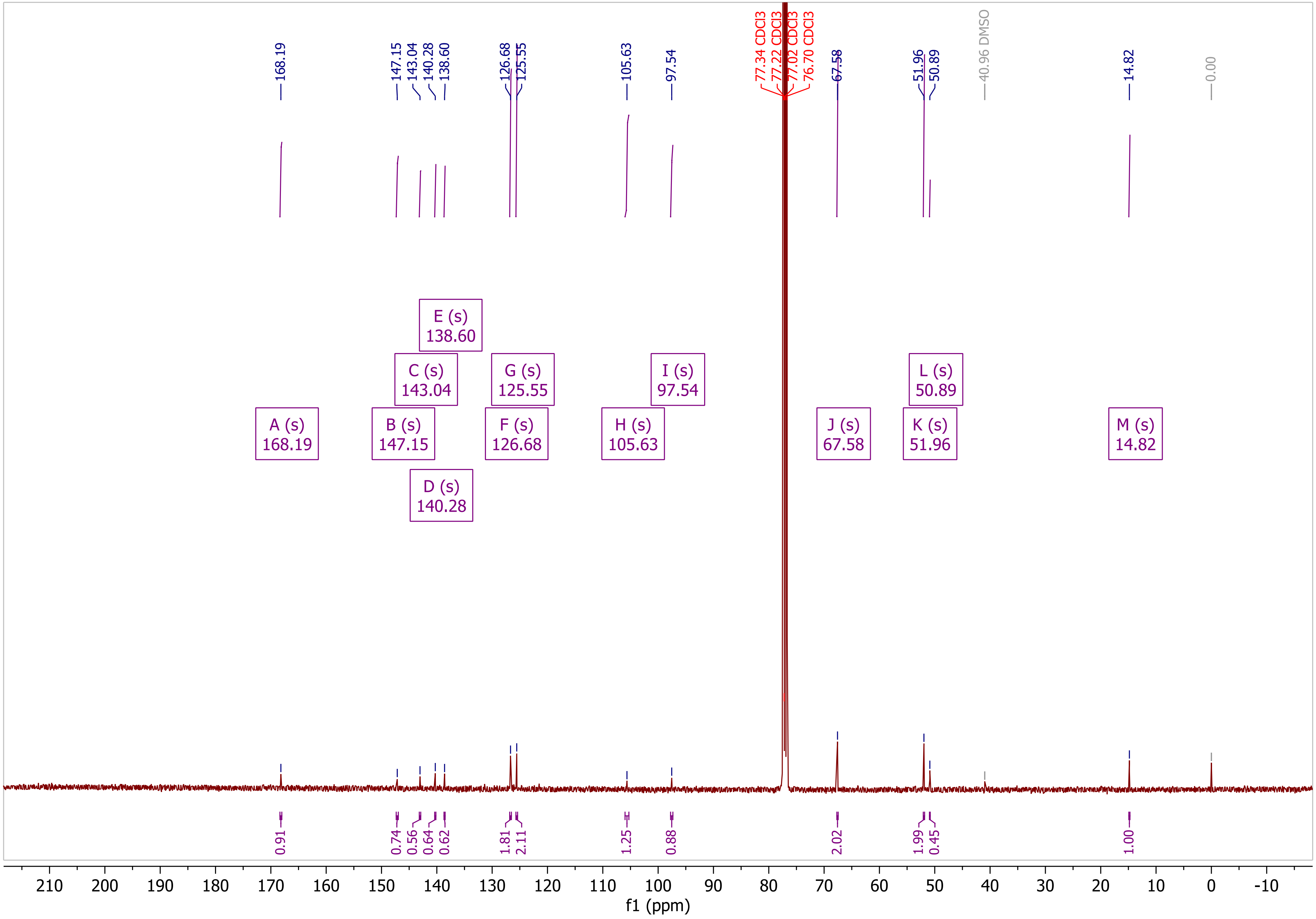

^{1}H NMR (400 MHz, $CDCl_3$) δ 8.65 (s, 1H), 8.23 – 8.16 (m, 1H), 7.80 (d, J = 2.5 Hz, 1H), 7.48 – 7.41 (m, 1H), 7.32 – 7.27 (m, 2H), 6.91 (s, 1H), 4.12 (q, J = 7.1 Hz, 1H), 3.88 (s, 4H), 3.40 – 3.29 (m, 1H), 2.95 (dt, J = 14.3, 6.6 Hz, 4H).

Compound 6

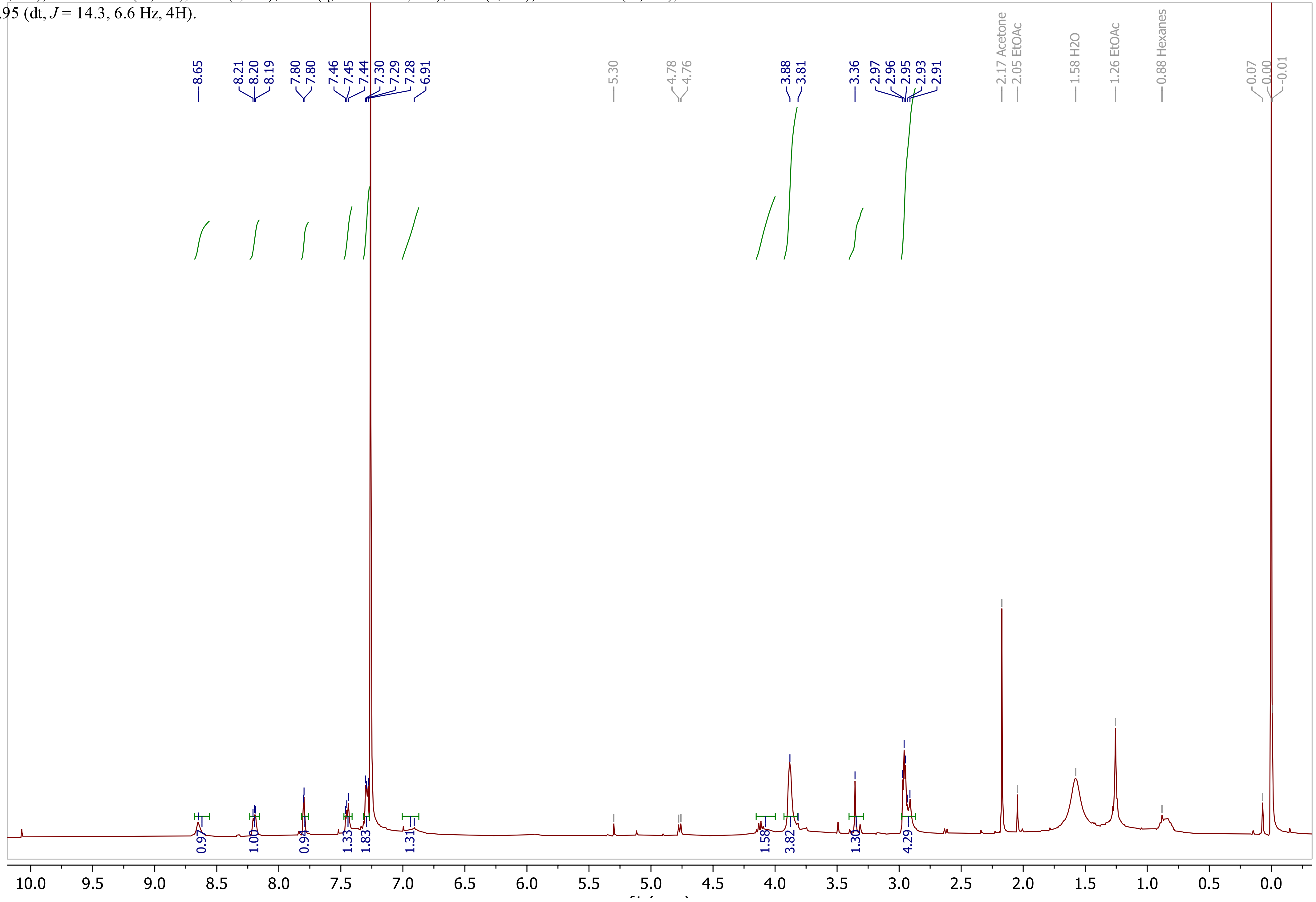

C NMR (101 MHz, $CDCl_3$) δ 153.42, 153.04, 142.92, 142.73, 141.55, 140.64, 139.62, 138.76, 130.34,
29.97, 129.92, 127.02, 126.01, 125.18, 125.14, 116.67, 112.09, 111.79, 102.87, 72.39, 72.05, 57.77,
7.38, 57.20.

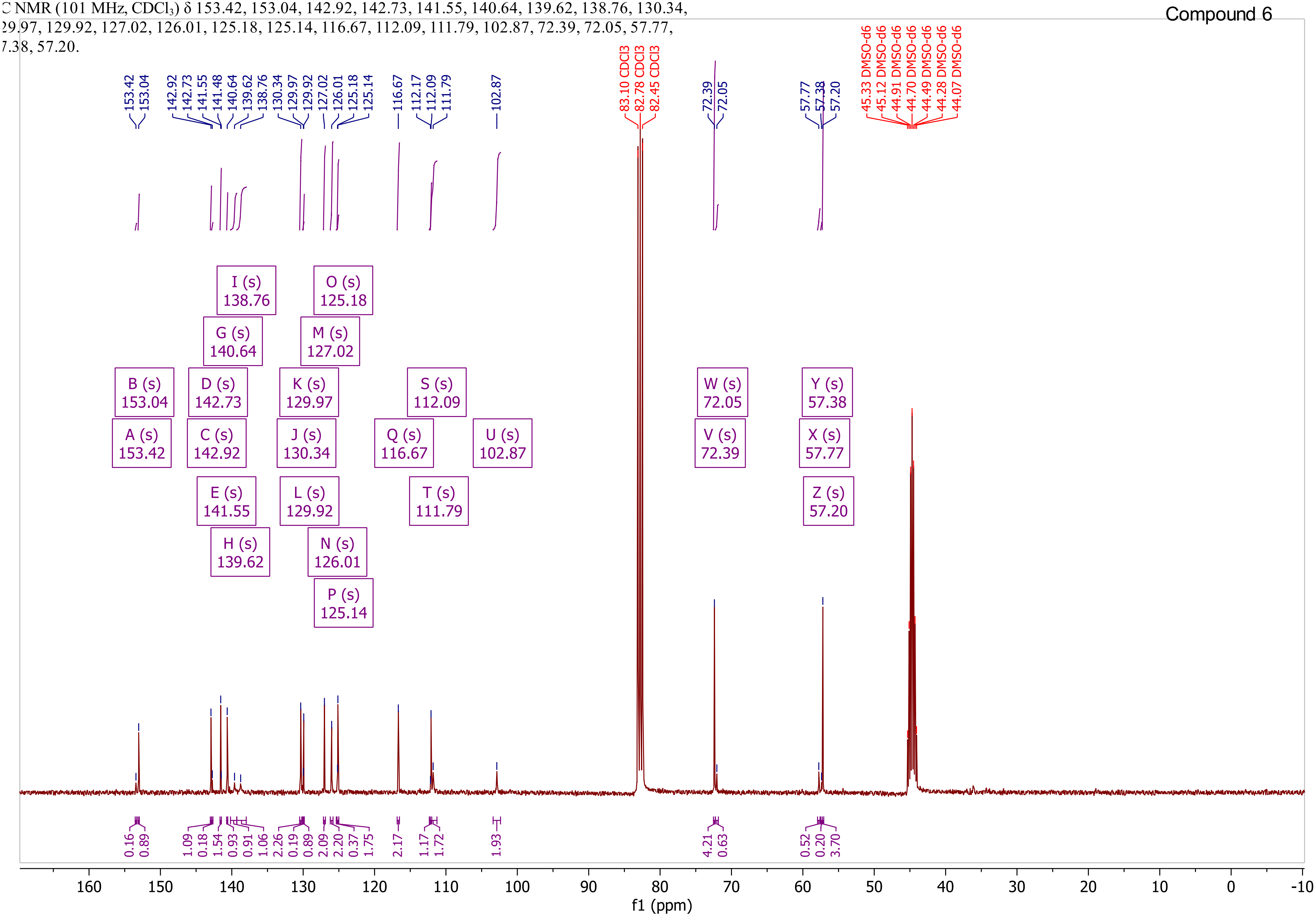

^{1}H NMR (400 MHz, $CDCl_3$) δ 7.69 – 7.50 (m, 2H), 7.45 (d, J = 11.1 Hz, 1H), 7.10 (d, J = 14.1 Hz, 4H), 6.70 (s, 1H), 6.62 – 6.47 (m, 2H), 3.82 (d, J = 17.9 Hz, 4H), 2.97 – 2.78 (m, 4H).

Compound 7

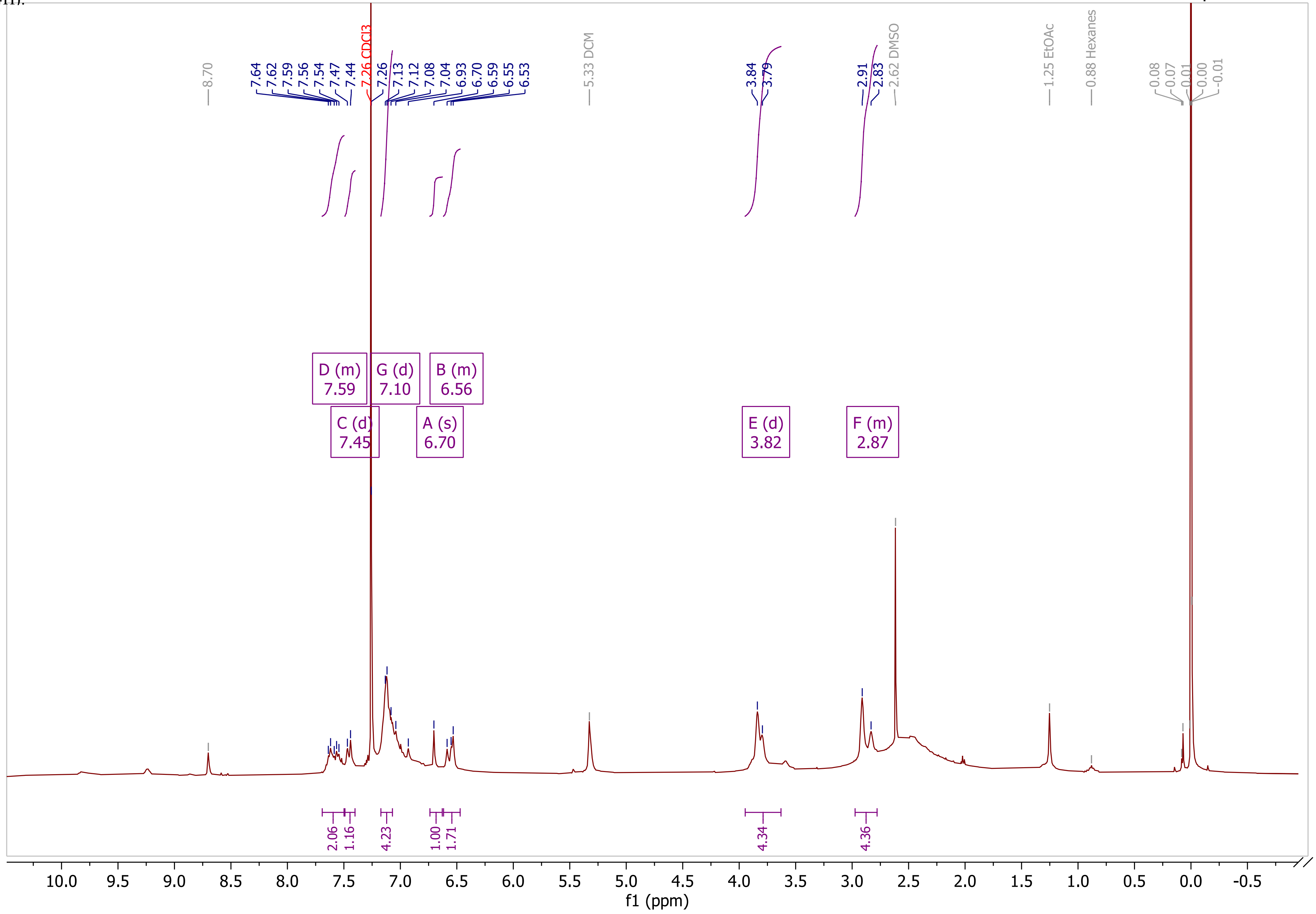

^{13}C NMR (101 MHz, $CDCl_3$) δ 157.35, 127.94, 126.86, 124.73, 116.30, 106.45, 95.51, 67.42, 53.59, 51.77, 32.41.

Compound 7

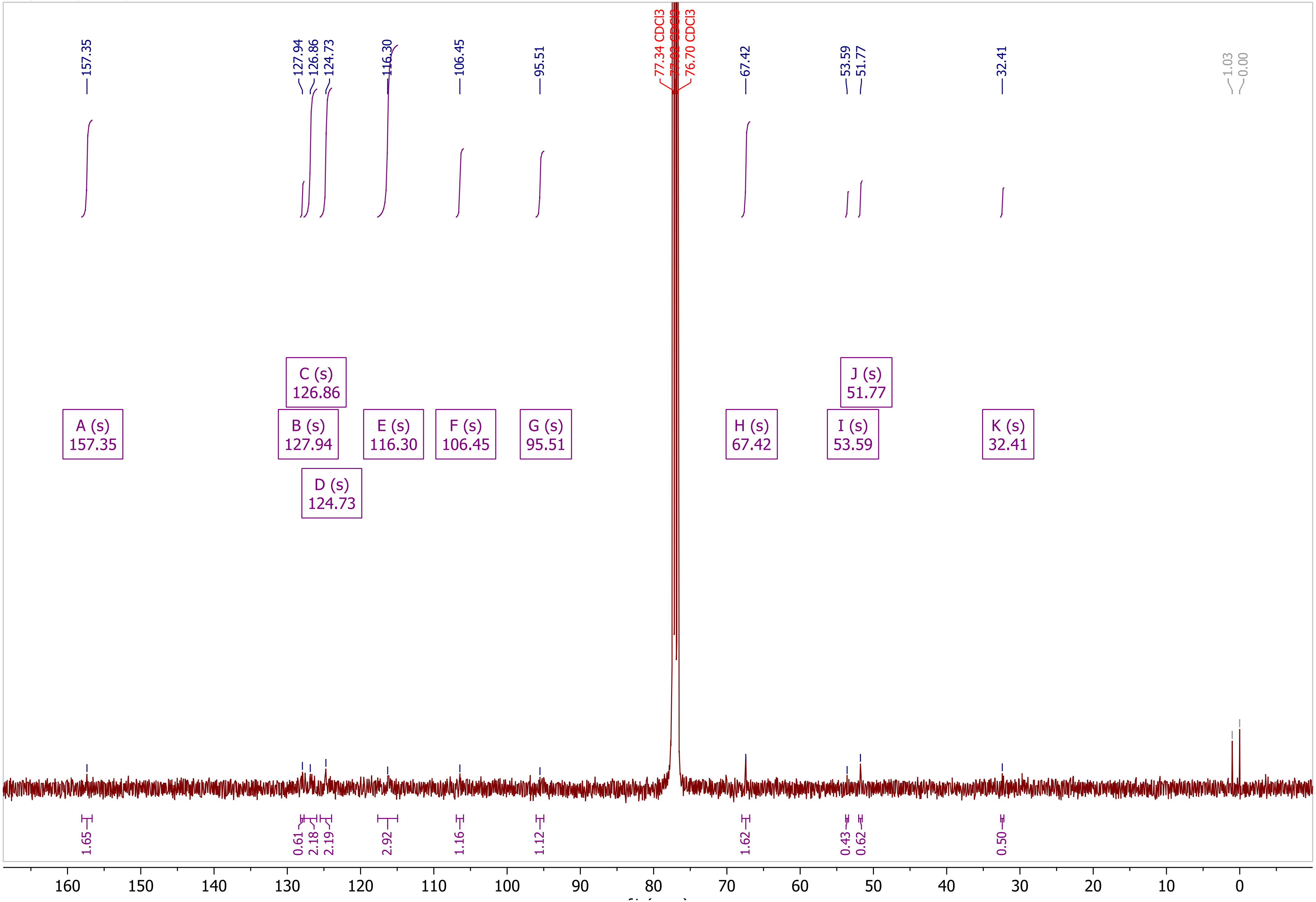

^{1}H NMR (400 MHz, $CDCl_3$) δ 8.37 (s, 1H), 7.95 (td, *J* = 7.6, 1.9 Hz, 1H), 7.34 – 7.29 (m, 1H), 7.25 – 7.21 (m, 1H), 7.14 (ddd, *J* = 11.4, 8.0, 1.3 Hz, 1H), 6.86 (t, *J* = 3.5 Hz, 1H), 6.83 (s, 1H), 3.91 – 3.80 (m, 4H), 2.91 (t, *J* = 4.5 Hz, 4H).

Compound 8

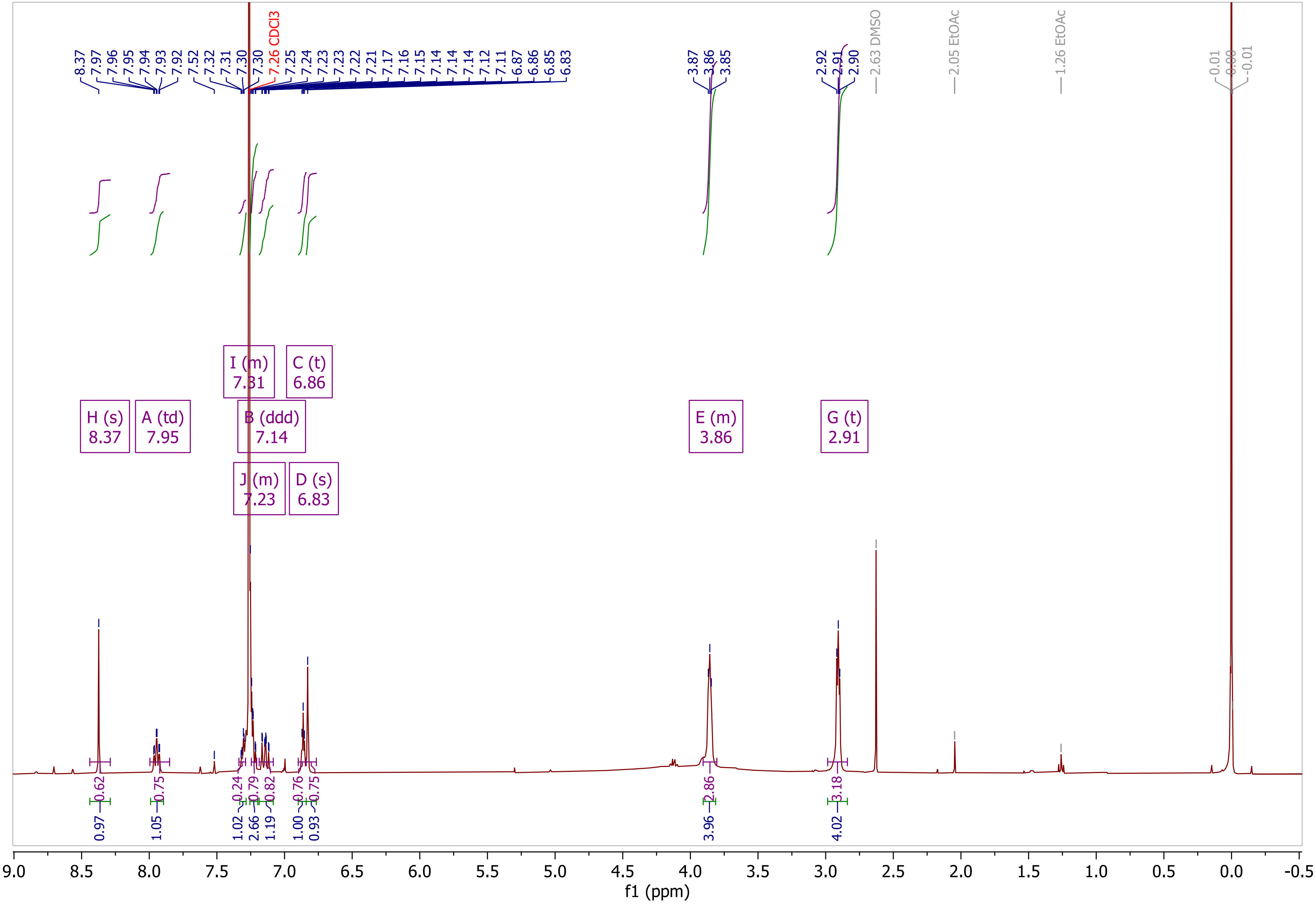

Compound 8

^{13}C NMR (101 MHz, $CDCl_3$) δ 160.02, 157.52, 148.95, 148.92, 144.78, 142.45, 139.38, 137.70, 128.93, 128.85, 126.09, 126.07, 124.24, 124.21, 118.25, 118.13, 116.02, 115.81, 112.64, 112.52, 112.02, 107.76, 98.22, 67.68, 52.29.

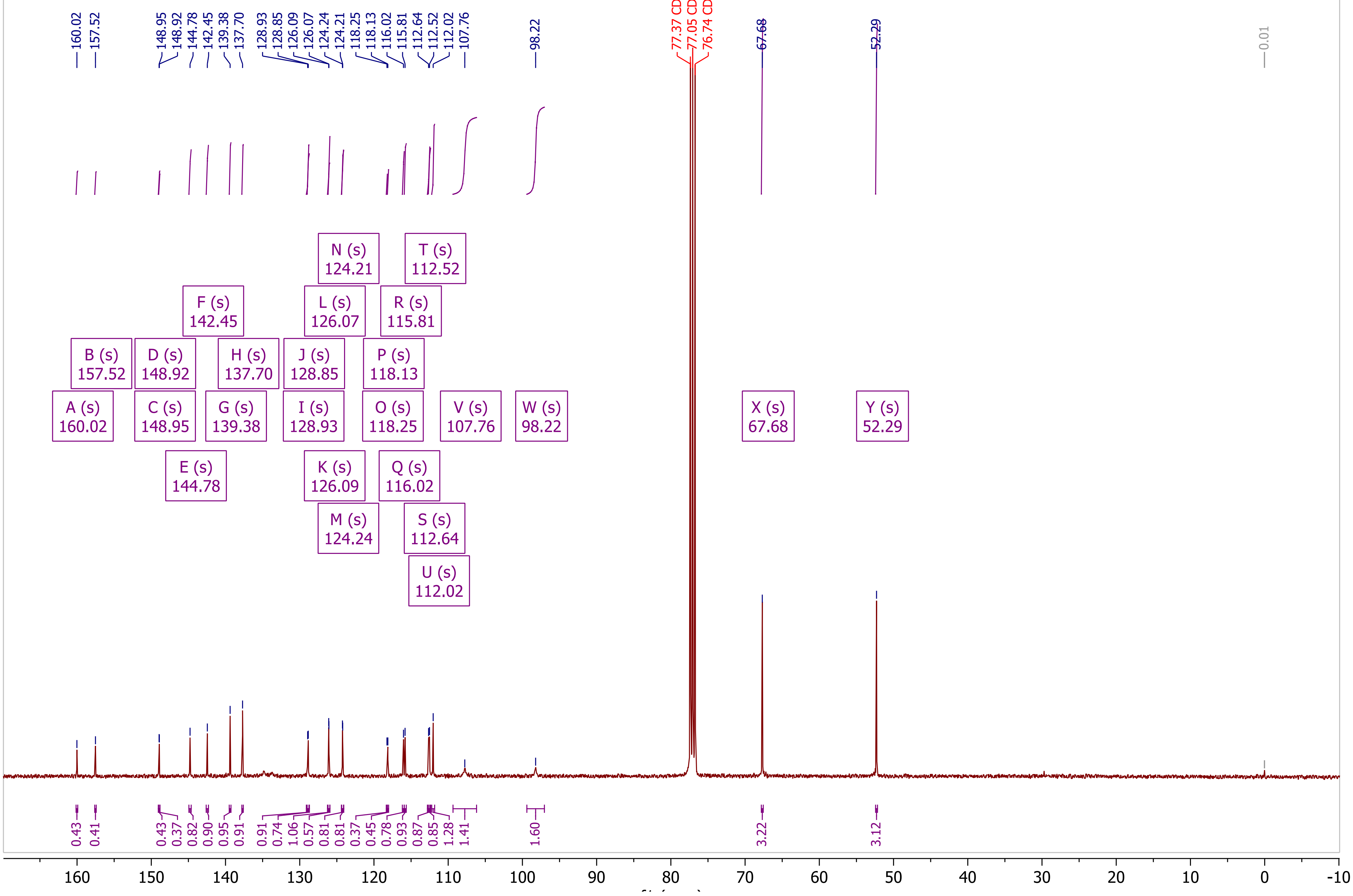

^{1}H NMR (400 MHz, DMSO) δ 8.17 – 8.10 (m, 1H), 8.10 (d, *J* = 2.1 Hz, 0H), 7.52 – 7.42 (m, 1H), 7.46 – 7.39 (m, 1H), 7.40 (t, *J* = 1.6 Hz, 0H), 7.37 (d, *J* = 1.6 Hz, 0H), 7.33 (s, 0H), 7.10 (t, *J* = 3.6 Hz, 1H), 3.77 – 3.69 (m, 2H), 3.03 – 2.96 (m, 2H).

Compound 8 2D reference spectrum

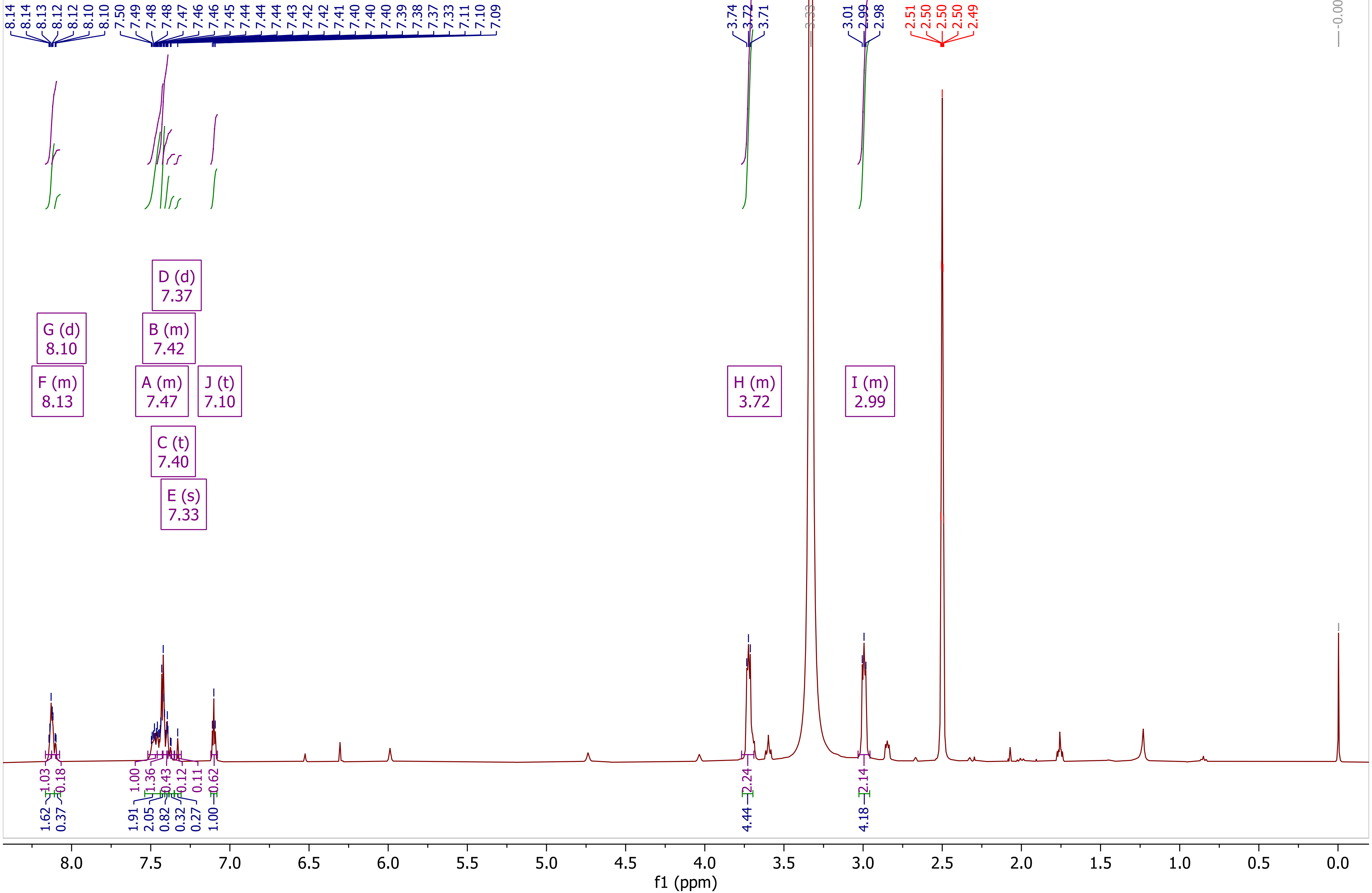

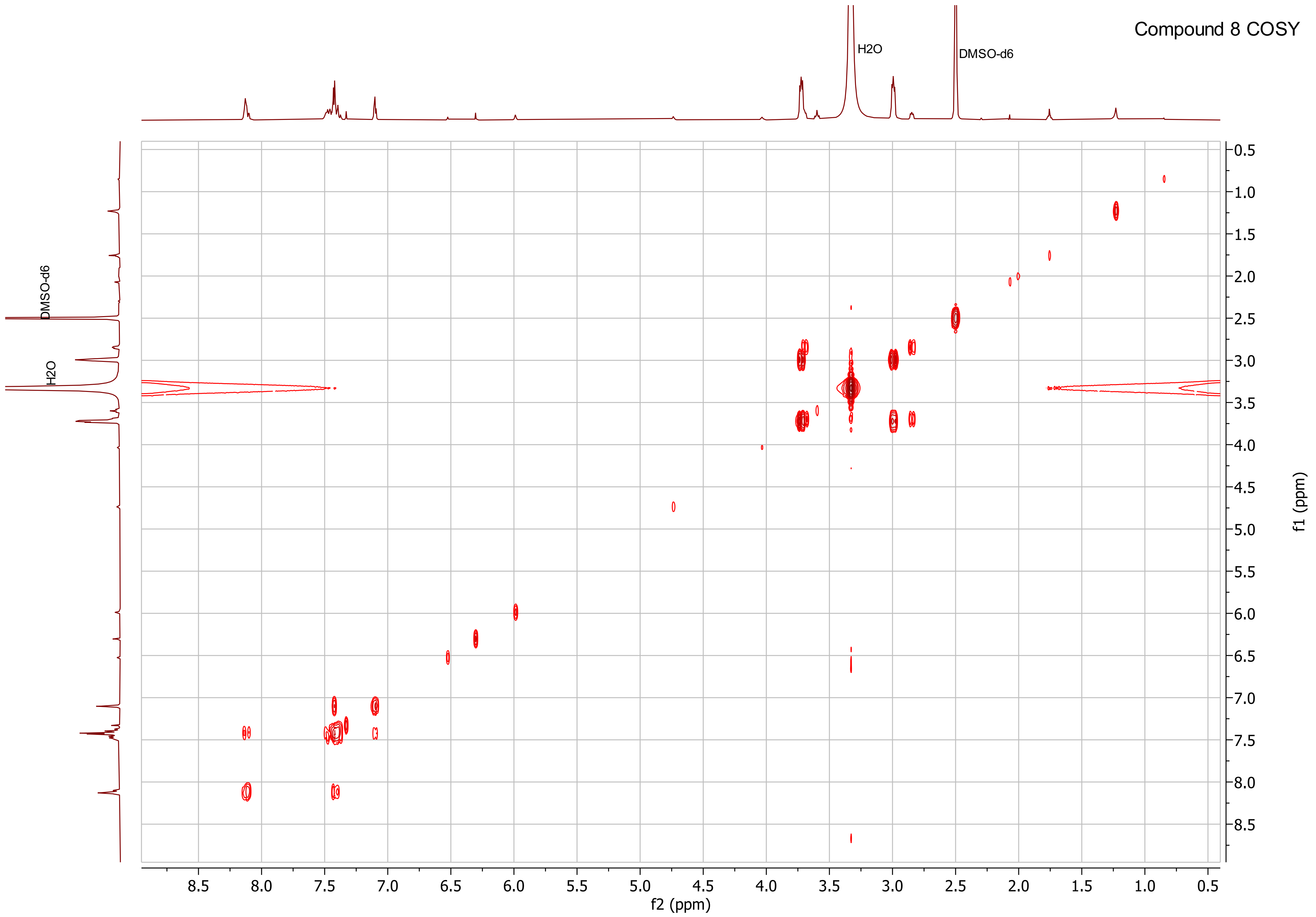

Compound 8 COSY
H2O
DMSO-d6
f2 (ppm)
f1 (ppm)

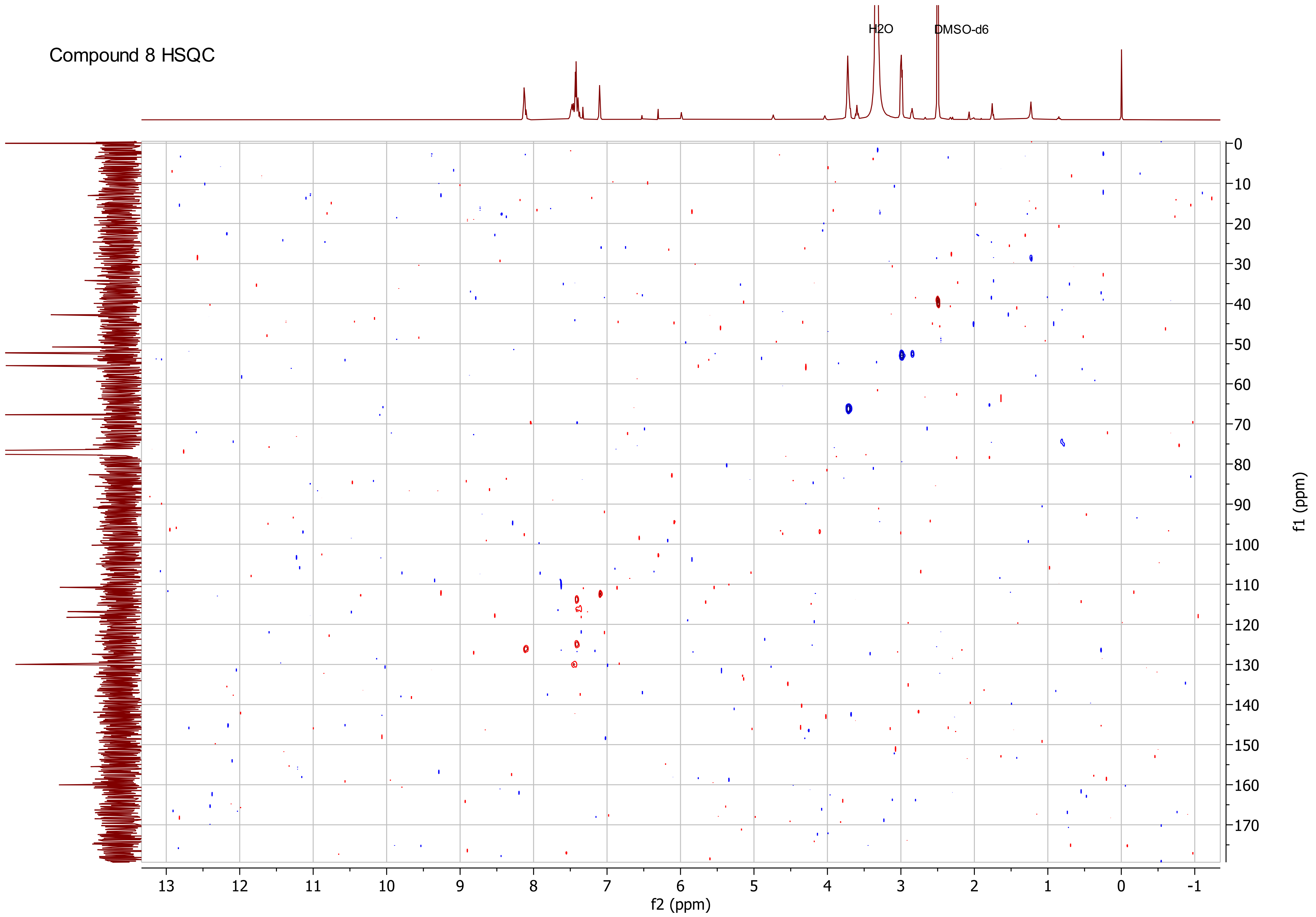

Compound 8 HSQC
H2O
DMSO-d6
13 12 11 10 9 8 7 6 5 4 3 2 1 0 -1
f2 (ppm)
0 10 20 30 40 50 60 70 80 90 100 110 120 130 140 150 160 170
f1 (ppm)
